# A549 in-silico 1.0: A first computational model to simulate cell cycle dependent ion current modulation in the human lung adenocarcinoma

**DOI:** 10.1371/journal.pcbi.1009091

**Published:** 2021-06-22

**Authors:** Sonja Langthaler, Theresa Rienmüller, Susanne Scheruebel, Brigitte Pelzmann, Niroj Shrestha, Klaus Zorn-Pauly, Wolfgang Schreibmayer, Andrew Koff, Christian Baumgartner

**Affiliations:** 1 Institute of Health Care Engineering with European Testing Center for Medical Devices, Graz University of Technology, Graz, Austria; 2 Research Unit on Ion Channels and Cancer Biology, Gottfried Schatz Research Center for Cell Signaling, Metabolism and Aging, Medical University of Graz, Graz, Austria; 3 Molecular Biology Program, Memorial Sloan Kettering Cancer Center, New York City, New York, United States of America; Virginia Polytechnic Institute and State University, UNITED STATES

## Abstract

Lung cancer is still a leading cause of death worldwide. In recent years, knowledge has been obtained of the mechanisms modulating ion channel kinetics and thus of cell bioelectric properties, which is promising for oncological biomarkers and targets. The complex interplay of channel expression and its consequences on malignant processes, however, is still insufficiently understood. We here introduce the first approach of an in-silico whole-cell ion current model of a cancer cell, in particular of the A549 human lung adenocarcinoma, including the main functionally expressed ion channels in the plasma membrane as so far known. This hidden Markov-based model represents the electrophysiology behind proliferation of the A549 cell, describing its rhythmic oscillation of the membrane potential able to trigger the transition between cell cycle phases, and it predicts membrane potential changes over the cell cycle provoked by targeted ion channel modulation. This first A549 in-silico cell model opens up a deeper insight and understanding of possible ion channel interactions in tumor development and progression, and is a valuable tool for simulating altered ion channel function in lung cancer electrophysiology.

## Author summary

Advances in the understanding of functional alterations at genetic, epigenetic or protein expression and the expanding knowledge in mechanisms modulating ion channel kinetics and thus the cells’ bioelectric properties have arisen as promising cancer biomarkers and oncological targets. Our hidden Markov-based in-silico cell model represents the electrophysiology behind proliferation of the A549 cell line, explaining the cell’s rhythmic oscillation from hyperpolarized to depolarized states of the membrane potential, able to trigger the transition between cell cycle phases. The model enables the prediction of membrane potential changes over the cell cycle provoked by targeted modulation of specific ion channels, leading to cell cycle promotion or interruption. We are encouraged that the availability of this first cancer cell model will provide profound insight into possible roles and interactions of ion channels in tumor development and progression, and may aid in the testing of research hypotheses in lung cancer electrophysiology.

## Introduction

Lung cancer is one of the most prevalent forms of tumor and the leading cause of cancer death worldwide.[1–3] Increasing knowledge of molecular cancer biology and the identification of key and potentially targetable genetic and molecular aberrations that drive tumor growth provide efficient diagnostic and therapeutic approaches for lung cancer. Somatic mutations of oncogenes and tumor suppressor genes in the lung adenocarcinoma appear to be promising therapeutic oncogenic targets.[4,5] Nevertheless, despite substantial advances in early diagnosis and innovative treatment strategies survival rates still remain poor.[6] Thus, a profound understanding of cancer biology at multiple functional levels will afford novel therapeutic agents to effectively fight and cure this disease.[7] In particular, advances in our understanding of molecular alterations at genetic, epigenetic or protein expression levels together with their functional significance, and in recent years, expanding knowledge in the mechanisms and modulation of ion channel function in cancer biology lead to the development of promising cancer biomarkers and oncological targets.[8,9]

Cells are characterized by a unique composition of ion channels responsible for the bioelectric properties of the cell, playing a fundamental role in almost all cellular functions. Cancer cells, compared to their differentiated benign counterparts, typically exhibit an altered ion channel expression or activity [8,10–12] associated with tumor development and progression [10–15]. Nevertheless, no unifying pattern could as yet be identified and expression levels appear to be diverse across different cancer types.[10] The expression profile of ion channels is decisive for the membrane potential *V*_m_, a key bioelectrical signal in the regulation of basic cellular activities such as proliferation, apoptosis, migration and differentiation.[10,14,16–18] In turn, voltage-gated ion channels (VGIC) respond to changes in the membrane potential through altered ion channel activation and conductivity, modulating the membrane potential for their part accordingly.[15,19] As a general principle, cancer cells tend to be more depolarized [19,20], whereby depolarization is assumed to initiate DNA-synthesis and mitosis.[10,16,21] During cell cycle progression, the membrane potential undergoes rhythmic oscillations starting with a further depolarization during the transition from the resting G0 to G1 phase, followed by a hyperpolarization during S phase initiation and subsequent depolarization while entering the M-G2 phase.[17,19] The exact thresholds required for driving the cells through the distinct phases have not been extensively studied and are likely to vary between different cell types.[17] It is well known, however, that the rhythmic oscillation occurs by means of a complex interplay between different predominantly hyperpolarizing, mainly voltage-dependent K^+^ channels (KCa, EAG, Kv, K_ATP_ K2P) and depolarizing ion channels such as the voltage-gated chloride channels (ClC) with cell cycle dependent expression levels.[16]

The crucial role of potassium conductance in governing the membrane potential and controlling the cell cycle has been supported in a number of studies.[22] It has been confirmed that blocking of potassium channels with selective inhibitors reduces proliferation in different cells.[23–29] For instance, inhibition of Kv channels reduces proliferation in prostate cancer cells [25] or analogously inhibition of Kv10.1 expression leads to reduced proliferation in diverse cancer cell lines [26]. However, it has still not been fully clarified to what extent the expression and activity of individual channels or channel-mediated changes of the membrane potential contribute to cell cycle progression, since inhibition of cell proliferation by channel blockage does not necessarily also lead to changes in the membrane potential.[10,23] Besides enhanced proliferation, expression levels of specific ion channels also correlate with other hallmarks of tumor progression such as evasion of apoptosis, sustained angiogenesis and invasion.[10,30,31] For example, Kv10.1 (EAG1) and Kv11.1 (hERG1) channels are linked to trigger angiogenesis [32–34], while inhibition of BK channels reduces migration of glioma cells [29] and the voltage-gated sodium channels Nav1.5 and Nav1.7 are generally associated with increased migration and metastasis.[10,35] By implication, targeted inhibition and activation of specific ion channels provides potential novel strategies for cancer therapy.[19,34] However, the complex interplay of channel expression, ion current dynamics together with their consequences on malignant processes are still insufficiently understood, in particular in the human lung adenocarcinoma.

In-silico modeling of whole-cell electrophysiology is a well-established tool for the description of the membrane potential in excitable cells. A range of models with different levels of complexity were introduced for simulating ion current kinetics and action potential alterations in neural or cardiac cells (e.g. neuronal model initially described by Hodgkin Huxley [36], or advanced cardiac cell models by Ten Tusscher [37] and Luo Rudy [38]). However, only a few approaches exist for non-excitable cells, e.g. the modulation of the membrane potential and cell secretion by single ion channels, calcium dynamics or activation of T-lymphocytes.[39–42]

The A549 cell line, derived from non-small cell lung cancer (NSCLC), is a widely used model for studying lung cancer and cancer drug development.[43–45] In this work we introduce for the first time an ion current model of the A549 human lung adenocarcinoma cell, representing an initial description of a cell model as a whole in cancer electrophysiology. The model takes into account the kinetics of the most relevant ion channels that contribute to the cell’s total membrane current and resting membrane potential. Based on our experimental data using the whole-cell patch-clamp technique and a comprehensive literature review (S1 Text), single channel kinetics were modeled by a hidden Markov model (HMM) approach and the number of represented ion channels estimated by fitting the macroscopic currents to the recorded whole-cell currents. The model was parameterized, taking into account the specific ion channel activities in the A549 cell obtained from the literature data and involves the main functionally expressed ion channels in the plasma membrane of the A549 cell known so far, also considering the respective voltage and calcium dependencies. This approach now allows for the first time the simulation of channel interaction, activation and inhibition, and, importantly, the prediction of membrane potential changes for parts of the cell cycle. The availability of this initial A549 in-silico model 1.0 provides a deeper understanding of the possible roles and interactions of ion channels in tumor development and progression and may aid in the testing, verification and validation of research hypotheses in lung cancer electrophysiology.

## Results

### Ion channels present in the A549 cell line

**Fig 1:**
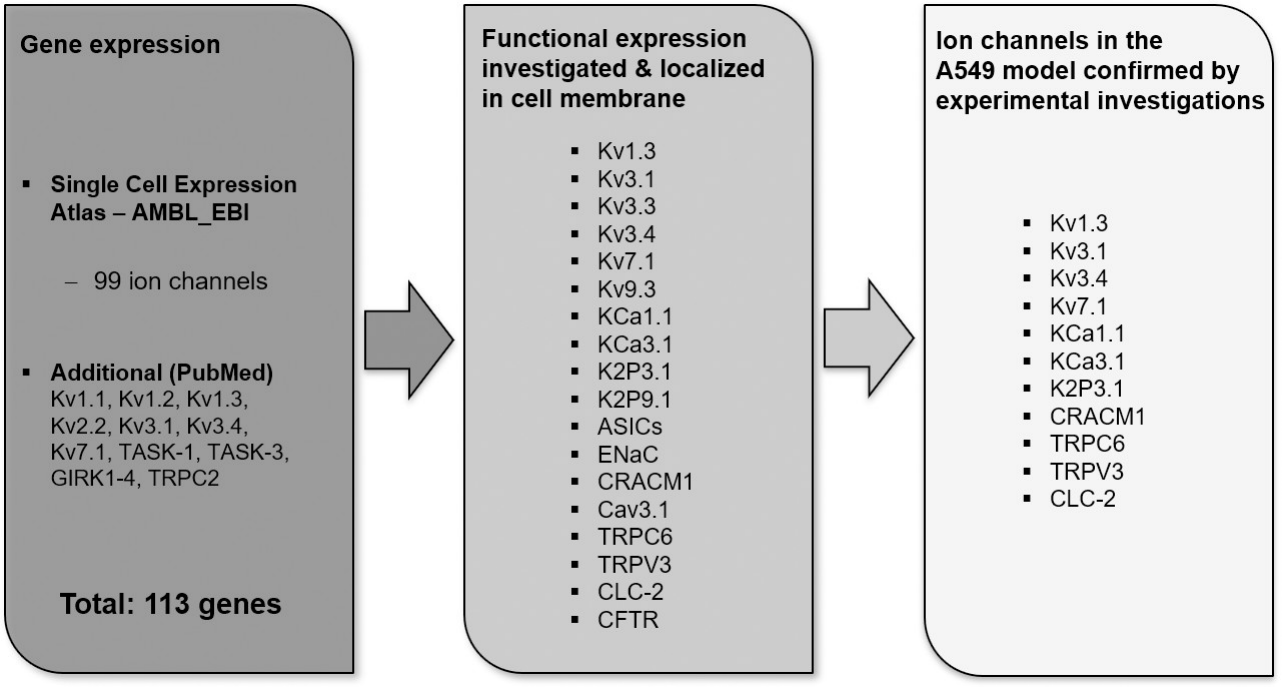
Reported ion channels in the A549 cell line. For detailed review see S1 Text.

A review of the gene database Single Cell Expression Atlas–AMBL_EBI revealed expression of 99 ion channel genes in A549 cells. Thereunder are 52 voltage-gated ion channels, 25 ligand-gated and 22, denoted as other ion channels according to the classification of IUPHAR/BPS (guide to pharmacology) including e.g. aquaporins, sodium leak channels or the CLC family.[46] Expression of 14 additional channel genes, not listed in the AMBL_EBI, were found in an additional PubMed literature search, leading in total to 113 ion channel genes expressed in A549 cells. Fig 1 provides a summary of reported ion channels in A549 cells reviewed in this work, building the fundamental basis for the A549 in-silico model (see S1 Text for review). However, the functional expression and localization of merely 18 channels in the cell membrane of A549 was confirmed by Western Blot and patch-clamp experiments in previous studies. 11 channels were finally included in this initial cell model, comprising the fast activating and inactivating channels Kv1.3 and Kv3.4 and channels with slower, but constant activation such as TASK-1 (K2P3.1) and KCa3.1 (hIK). These channels together are decisive for the instantaneous activating current whereas Kv7.1, Kv3.1 and also KCa1.1 account for the time-dependent increase of the membrane current. The calcium entry caused by CRACM1, TRPC6 and TRPV3, and the CLC-2 mediated chloride current provoke a cumulative negative inward current, which balances the positive potassium outward current. The remaining seven to 18 channels, i.e. Kv3.3, Kv9.3, K2P9.1, ASICs, ENaC, Cav3.1 and CTFR, were not considered in the model, because the kinetics of these channels are not fully known and experimental data on them is not yet available. This level of abstraction and simplification can scarcely be regarded as representing a limitation to this “whole-cell” model, since the implemented ion channels appear to be sufficient for a valid characterization of the physiological cell function over the cell cycle.

### The HMM based A549 whole-cell current model

**Fig 2:**
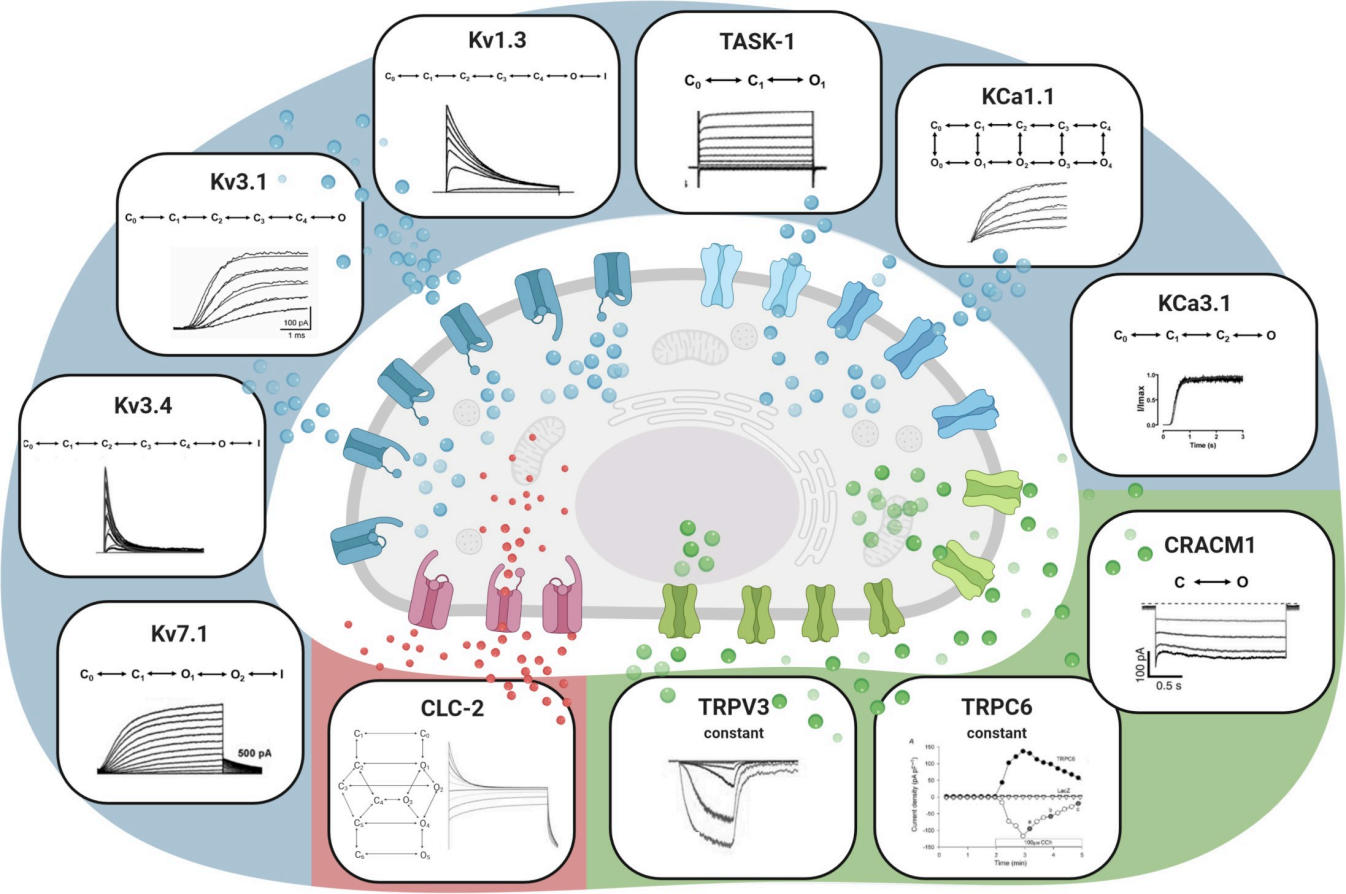
A549 whole-cell ion current model. The model illustrates the different ion channel types, macroscopic currents and kinetic schemes of the used hidden Markov models (HMM). Potassium channels, including Kv1.3, Kv3.1, Kv3.4, Kv7.1, TASK-1, KCA1.1 and KCa3.1 are represented in blue, green denotes the included calcium channels CRACM1, TRPV3 and TRPC6 and red represents the considered chloride channel CLC-2. See also S1 Fig. Created with BioRender.com.

Fig 2 illustrates the A549 whole-cell model, indicating the different ion channel types, macroscopic currents and their kinetic schemes using a hidden Markov modeling (HMM) approach. HMMs represent the gating of an ion channel through a series of conformational changes of the channel protein, assuming that the transition probability between these states depends on the present state only. Exemplarily, for the ion channel Kv7.1 a five state HMM consisting of two closed (C), two open (O) and one inactivated state (I) was implemented in accordance with the kinetic model described by Pusch et al. [47]:

$$C_{1}\overset{\alpha }{\underset{\beta }{⇌}}C_{2}\overset{a}{\underset{b}{⇌}}O_{1}\overset{c}{\underset{d}{⇌}}O_{2}\overset{\eta }{\underset{\lambda }{⇌}}I$$

The forward transition rates *α*, *a* and *c*, and backward transition rates *β*, *b* and *d* for the transitions between open and closed states (C_1_⇌C_2_⇌O_1_⇌O_2_) are voltage dependent and given in the form:

$\alpha =\alpha_{1}∙{exp}^{\left(\frac{\alpha_{2}∙V∙F}{R∙T}\right)}$ and $\beta =\beta_{1}∙{exp}^{\left(\frac{\beta_{2}∙V∙F}{R∙T}\right)}$, where *α*_*i*_ and *β*_i_ represent specific gating parameters, *V* the applied voltage, *F* the Faraday constant, *R* the gas constant and *T* the absolute temperature; while *η* and *λ* describe transition rates for the transition between the open and the inactivated state (O_2_⇌I).

Defining $P_{S_{i}}(t)$ as the probability of being in a specific state *S*_*i*_ at time *t* leads to the equation for the time evolution of the channels’ open probability $P_{O_{1}}(t):\frac{{dP}_{O1}}{dt}=P_{C_{2}}(t)∙a+P_{O_{2}}(t)∙d-P_{O_{1}}(t)∙(b+c)$, where the first two terms represent all transitions entering state O_1_ and the rightmost term all transitions leaving state O_1_. For sufficiently large numbers of the same channel, the quantities in this equation can be replaced by their macroscopic interpretation and the probability of being in a state *S*_*i*_ can be interpreted as the fraction of channels in *S*_*i*_. The transition probabilities (transition rates times dt) can be described as rate constants, *r*_*i*,*j*_, defining the number of channels changing from *S*_*i*_ to *S*_*j*_ in a given time period.[48] The total open probability ($P_{O}=\sum_{k}P_{O_{k}},k=1\ldots$ number of open states), the ion channel number (*N*_c_*)*, the single channel conductance (*g*) and reversal potential of the ion (*E*_ion_) allow the calculation of the channels’ macroscopic current *I*(*t*,*V*) = *N*_c_∙*P*_O_(*t*)∙*g*∙(*V*−*E*_ion_). The total membrane current results from the sum of the individual macroscopic currents considered and can be denoted as:

$$I_{whole\_cell}=I_{Kv1.3}+I_{Kv3.1}+I_{Kv3.4}+I_{Kv7.1}+I_{KCa3.1}+I_{KCa1.1}+I_{TASK-1}+I_{CRACM1}+I_{TRPC6}+I_{TRPV3}+I_{CLC-2}$$ (1)

Since various electrophysiological phenotypes of the A549 cell could be predicted with high accuracy during the cell cycle, no additional leak current, summarizing all remaining channel activities, needed to be introduced.

In detail, HMMs were introduced in various studies [39,47,49–51] for the voltage-gated ion channels Kv1.3, Kv3.1, Kv3.4, Kv7.1 and CLC-2. The voltage-sensitive, but ligand-gated channel KCa1.1, pH and voltage-sensitive TASK-1 channels as well as the solely ligand-gated channel KCa3.1 were implemented according to Wang et al. [52], Limberg et al. [53] and Bailey et al. [54]. For CRACM1, a two state HMM (C⇌O) was defined and the corresponding rate constants for determining the open probability *P*_O_ were derived from the measured, voltage-dependent closed and open lifetimes of this channel.[55] A constant current under consideration of the voltage-dependent conductivity was however implemented for the ligand-gated channels TRPC6 and TRPV3. The individual HMMs and corresponding rate constants of all ion channels are provided in S1 Fig and S1 Table.

The global cytosolic calcium concentration measured in A549 cells is about 64.7 ± 2.5 nM [56]. KCa3.1 (hIK) channels are activated at calcium concentrations greater than 200 nM and reach maximal activity at 1 μM.[57] Such high calcium levels required for activation indicate a close proximity of KCa3.1 to CRAC channels, where local calcium concentrations are much higher.[58,59] In the context of the calcium dependent gating of KCa3.1 and KCa1.1, the calcium inflow of CRACM1 channels is converted to a local calcium concentration by a formalism to depict the interaction between those channels in the model [39,60,61]:

$$\frac{d[{Ca}^{2+}{]}_{i}}{dt}=e_{trans}∙(-I_{CRAC})-e_{diff}∙[{Ca}^{2+}{]}_{i}$$ (2)

with

$$e_{trans}=\frac{1}{z∙F∙{Vol}_{compart}}$$

where [*Ca*^*2+*^]_*i*_ is the intracellular calcium concentration, *e*_trans_ a transfer coefficient, scaling the calcium influx within a certain intracellular subspace, in which the ion channels reside, to the calcium concentration, and *e*_diff_ represents a calcium diffusion coefficient.[61]

Following Hou et al.[39], *e*_diff_ was set to 3∙10^−3^ ms^-1^. The transfer coefficient *e*_trans_ was estimated based on the cell volume, taking into account the mean measured cell capacitance *C*_measured_ = 33.4 pF and the specific A549 cell capacitance *C*_specific_ = 2.45 μFcm^-1^ [62]. We assumed a cell compartment of 5% of the total cell volume (*Vol*_compart_ = 2.336∙10^−14^ L), resulting in a transfer coefficient *e*_trans_ of 21.9∙10^−3^ μMpA^-1^ms^-1^L^-1^.

CRAC channels are highly active at negative voltages, whereas their outward current, carried by Na^+^ at positive voltages, is negligible.[63] Changes in the calcium concentration provoked by CRAC channels occur slowly over time and thus take much longer than the applied test pulses. Hence, in order to reach the calcium concentration needed to trigger KCa3.1 and KCa1.1 activity, the steady state value after 10 seconds at a holding potential of -100 mV was taken as the input parameter for optimization and simulation. Nevertheless, the CRAC current evoked at negative voltages is considered in a time-dependent manner for the estimation of individual channel numbers and simulation of the initial and post pulse of performed patch clamp measurements. The time dependent evolution of the local calcium concentration is illustrated in S2 Fig.

### Patch-clamp measurements of whole-cell membrane currents for model parametrization

A total of *n* = 16 of originally *n* = 50 A549 cell preparations, which met our internal lab standards (see Methods section), were considered for this study. A voltage-step protocol was applied consisting of an initial and re-pulse of -80 mV for 100 ms and a series of voltage pulses from -40 mV to +40 mV (increment 10 mV) of 800 ms duration. For model optimization, averaged whole-cell current curves were selected and used for fitting the whole-cell current simulated by the model. In addition, voltage-ramp measurements for determining the reversal potential and model verification were performed. The holding potential was set to -100 mV between all recording modalities. For detailed information on electrophysiological recordings, quality criteria and data pre-processing see “Methods” section.

**Fig 3:**
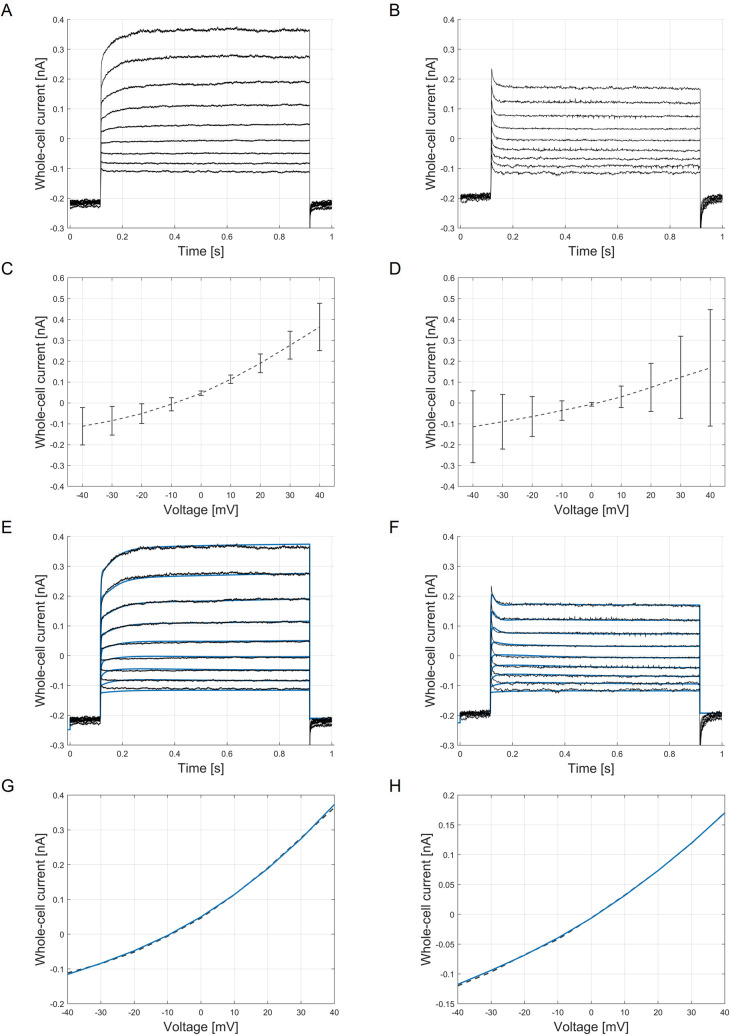
Measured and simulated whole-cell current response. Averaged whole-cell current curves measured at voltage-steps from -40 mV to +40 mV for (A) cells with negative resting membrane potential, G0 phase (*n* = 11) and (B) positive resting membrane potential, G1 phase (*n* = 5). (C) Averaged current-voltage curve of cells in G0 phase (mean ± SD): -40 mV: -0.1114 ± 0.0850 nA, -30 mV: -0.0849 ± 0.0671 nA, -20 mV: -0.0514 ± 0.0454 nA, -10 mV: -0.0059 ± 0.0292 nA, 0 mV: 0.0464 ± 0.0113 nA, +10 mV: 0.1137 ± 0.0286 nA, +20 mV: 0.1903 ± 0.0560 nA, +30 mV: 0.2770 ± 0.0920 nA, +40 mV: 0.3644 ± 0.1327 nA. (D) Averaged current-voltage curve of cells in G1 phase: -40 mV: -0.1140 ± 0.1721 nA, -30 mV: -0.0900 ± 0.1310 nA, -20 mV: -0.0649 ± 0.0960 nA, -10 mV: -0.0364 ± 0.0467 nA, 0 mV: -0.0065 ± 0.0088 nA, +10 mV: 0.0294 ± 0.0516 nA, +20 mV: 0.0744 ± 0.1150 nA, +30 mV: 0.1233 ± 0.1968 nA, +40 mV: 0.1685 ± 0.2794 nA. Comparison of simulated whole-cell current kinetics (blue lines) and experimental data (black lines) for the applied voltage-step protocol, and the corresponding current-voltage curves for cells in the G0 phase (E, G) and for cells in the G1 phase (F, H). *RMSE* values for simulation of activation curves are for the (E) G0 phase: *RMSE* = 0.0083 and (F) G1 phase: *RMSE* = 0.0074. *RMSE* values for current-voltage curves are for the (G) G0 phase: *RMSE* = 0.0031 and (H) G1 phase: *RMSE* = 0.0016. Averaged measured and simulated reversal potentials from derived current-voltage curves in the G0 phase are: *V*_rev_measured_ = -8.9 mV, *V*_rev_simulated_ = -9.3 mV (G) and in the G1 phase: *V*_rev_measured_ = 1.8 mV, *V*_rev_simulated_ = 0.95 mV (H). A comparison of the macroscopic ion currents in G0 phase and G1 phase can be found in S3–S7 Figs.

Depending on the measured resting membrane potential and whole-cell current kinetics we were able to subdivide the cells into two groups, indicating the resting G0 and G1 phase of the A549 cell cycle. In detail, cells with a negative potential (*V*_rest_ = -40 mV to 0 mV, *n* = 11) showed a high instant activating current followed by a more slowly, time-dependent increase of the current (G0 phase). By contrast, cells with a highly depolarized, positive membrane potential (*V*_rest_ = 0 mV to +20 mV, *n* = 5) exhibited a comparable instantaneous current, but a lack of the time-dependent current, which resulted in lower steady currents (G1 phase) (see Fig 3A and 3B). The corresponding current-voltage curves were generated by selecting the steady state currents at 99% of the test pulse length. Averaged current-voltage curves with the corresponding standard deviations ($\bar{x}\pm \sigma$) for both cell groups are shown in Fig 3C and 3D. These variations can be explained by the expected heterogeneity in the morphology and membrane conductivity of the cell population.

**Fig 4:**
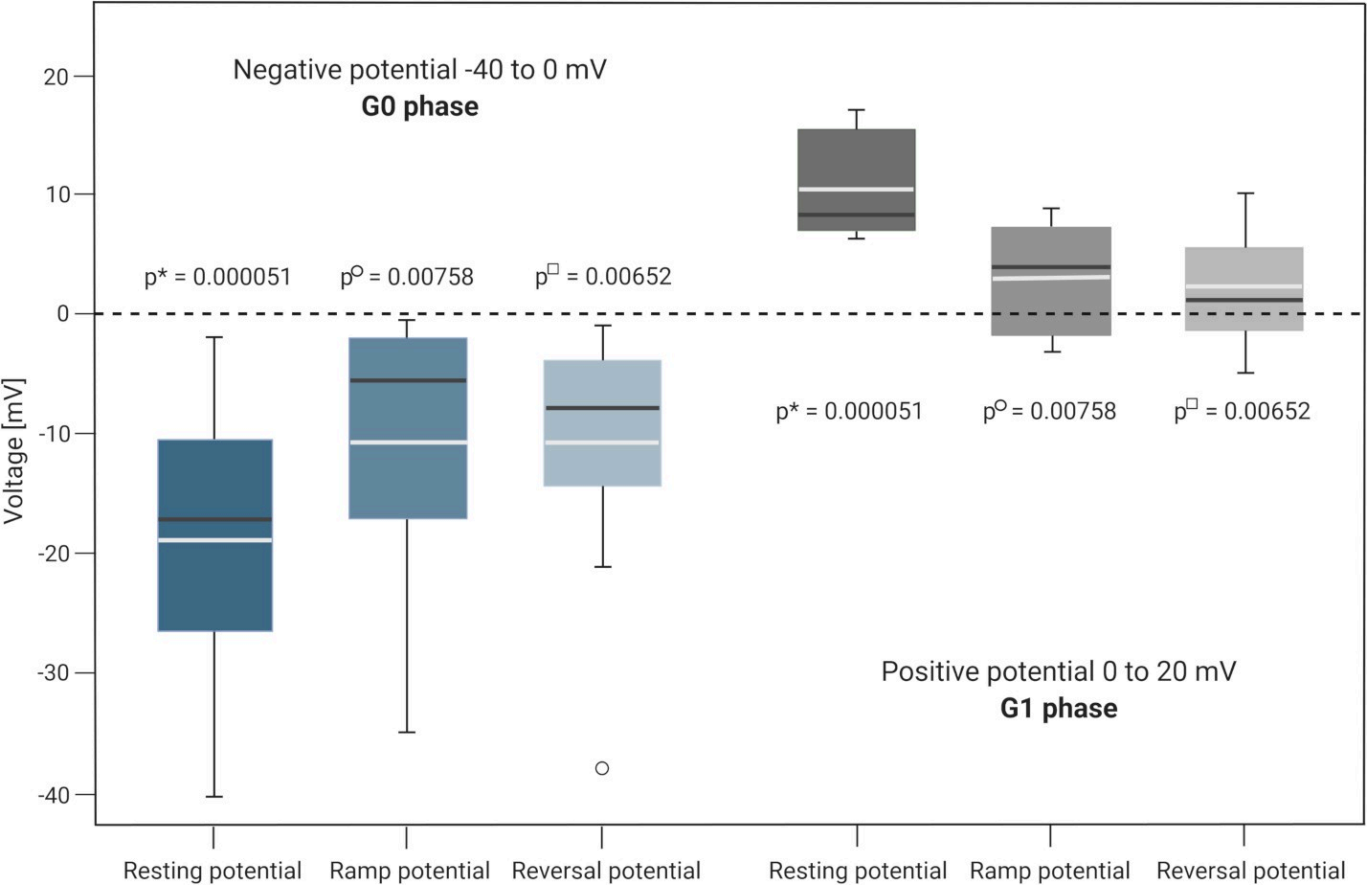
Resting potential, ramp potential and reversal potential in G0 and G1 cell cycle phase. Resting membrane potentials measured in current-clamp mode, reversal potentials from voltage-ramp measurements and derived reversal potentials from current-voltage curves in negative (G0 phase) and highly depolarized (G1 phase) A549 cells. Black lines in the boxplot indicate the median, white lines the mean value. Whiskers denote 10% and 90% percentiles. Single scores outside the 10% and 90% percentile are depicted as black circles. P-values demonstrate statistical significance between cells in G0 and G1 phase. Detailed measurement results are provided in S2 Table. Created with BioRender.com.

Fig 4 illustrates the measured resting membrane potentials, recorded reversal potentials from voltage-ramp measurements and reversal potentials from the generated current-voltage curves of the cells within the respective phases G0 and G1. Resting membrane potentials *V*_rest_, reversal potentials *V*_rev_ determined by voltage-ramp measurements and derived from current-voltage curves differed statistically significantly between cells in G0 vs G1 phase. For detailed information on membrane potential measurement and statistics see “Methods” section and S2 Table.

### Model optimization and parametrization for simulation of the current-time characteristics based on experimental data

The HMM-based whole-cell model was implemented in the simulation environment MATLAB (MathWorks Inc.). Since HMMs model the open and closing probabilities of single ion channels stochastically, we instead used the macroscopic interpretation of the open probabilities *P*_O_ to simplify model optimization in a deterministic manner and thereby reduce computational cost. Based on these open probabilities (*P*_O_), the macroscopic currents of all selected ion channel types were optimized by estimating the channel numbers ($N_{c_{x}}$) for the measured whole-cell currents using a particle swarm optimization (PSO) based approach:

$$I_{simulated}=\sum_{x=1}^{m}N_{c_{x}}∙P_{Ox}(t)∙g_{x}∙(V-E_{ion})x:\;individual\;ion\;channels$$ (3)

This leads to an ordinary bounded integer least squares problem defined as [64]:

$$\underset{N\in Z^{m}}{\min}{\left\|y-AN\right\|}_{2}^{2},B=\{N\in Z^{m}:L\leq N\leq U\}$$ (4)

where $y\in R^{n\times 1}$ is the average measured total membrane current, $A\in R^{n\times m}$ is the matrix of the individual open probabilities and single channel conductivities $P_{Ox}(t)∙g_{x}∙(V-E_{ion}),\;N\in Z^{m}$, a vector containing the number of channels $N_{c_{x}}$ per channel type *x* and **L** and **U** are the lower and upper bounds of the individual channel numbers, respectively.

The model parameters are denoted in Table 1, showing the channel conductance (*g*), reversal potential of ions (*E*_ion_), bounds on the channel number for optimization and the estimated channel numbers for each single ion channel. The reversal potentials were calculated by the Nernst equation from the internal and external solutions used. Based on our experimental data and the available literature data, constraints for an adequate fitting of the measured whole-cell current curves were taken into account and set as follows: The minimal number of channels was set to 5 for Kv3.1, Kv3.4, Kv7.1, KCa1.1 and 10 for TASK-1, TRPC6, TRPV3 and CLC-2. Upper bounds were set to 150 for Kv3.1, 100 for Kv3.4 and CLC-2, 1350 for Kv7.1, 50 for KCa1.1 and 20 for TRPC6 and TRPV3. For Kv1.3 an amount of 20% and 80% [65] of the instant current were assumed as lower and upper bounds. The activity of KCa3.1 is strongly related to the cell cycle with which the current varies between 30% and 90% of the instant current depending on the membrane potential [66]. Cells with a hyperpolarized membrane potential exhibit a higher activity compared to cells with a depolarized membrane potential.[66] To account for the cell cycle dependent hIK activity in A549 cells, we set the lower bound for model optimization in the G0 phase to 50% of the instant current. For the G1 phase, 30% and 90% of the instant current were assumed as lower and upper bounds. In Leithner et al. [67], the TASK-1 current was determined to be about 80 pA (voltage level +40 mV) in hyperpolarized cells, estimated by 50 channels in the TASK-1 model. Thus, for TASK-1 the lower and upper bounds were set to 10 and 100 channels. The number of CRAC channels was set to 200 [68].

**Table 1:** A549 model parameters and estimated ion channel numbers from model optimization.

Ion channel	Single channel conductance	E_ion_	Constraints		Estimated channel number	
			Lower	Upper	Negative cells G0—phase	Positive cells G1—phase
Kv1.3	15 pS [39,70]	-77.4 mV	20% of instant current^a^: 15 (G0), 20 (G1)	80% of instant current^a^: 59 (G0), 80 (G1)	22	20
Kv3.1	40 pS [49]		5	150	78	90
Kv3.4	14 pS [71]		5	100	5	54
Kv7.1	3.2 pS [72]		5	1350	1350	558
KCa1.1	250 pS [52]		5	50	40	15
KCa3.1	11 pS [39,57]		50% and 30% of instant current^a^: 77 (G0), 63 (G1)	90% of instant current^a^: 139 (G0), 188 (G1)	77	63
TASK-1	16 pS [73]		10	100	19	10
CRACM1	24 fS [68,74]	+95.6 mV	200		200	200
TRPC6	35 pS [75]		10	20	17	12
TRPV3	48 pS (negative voltages), 197 pS (+40 mV to +80 mV) [76,77]		10	20	12	13
CLC-2	2.8 pS [78]	-7.9 mV	10	100	13	11

a) At voltage-level +40 mV.

The measured whole-cell current curves (Fig 3A and 3B) obtained from voltage-step protocols at all voltage steps (-40 mV to +40 mV) were used for optimization. To optimize the number of ion channels instead of the least square error (see Eq 4), the relative root-mean square error (*RRMSE*) between the simulated (*I*_model_) and measured current (*I*_data_), divided by the root mean square distance of *I*_data_ to a zero current trace, was minimized using particle swarm optimization [69]:

$$RRMSE=\sqrt{\sum \left(I_{model}(t\right)-I_{data}(t){)}^{2}/\sum I_{data}(t{)}^{2}}$$ (5)

where

$$I_{model}(t)=\sum {(N}_{x}∙P_{Ox}(t)∙g_{x}∙(V-E_{ion}))$$

Optimization by fitting the simulated curves to the experimental data was estimated using the averaged root mean square error values (*RMSE)* over all voltage steps:

$$RMSE=\sqrt{\sum \left(I_{model}(t\right)-I_{data}(t){)}^{2}/N}$$ (6)

Optimization results of the current time series data of cells assumed to be in the G0 and G1 cell cycle phase are shown in Fig 3E and 3F. The specified constraints (lower and upper bounds of the channel numbers) allowed a precise simulation of the current-time characteristics of the two phases, demonstrating an almost perfect fit (*RMSE*_G0_ = 0.0083, *RMSE*_G1_ = 0.0074) of the simulated whole-cell current kinetics (blue lines) to the experimental data (dashed black lines). In particular, the steady-state currents coincide well with the measured current amplitudes as shown in the derived current-voltage curves (Fig 3G and 3H). The source code of the A549 model for optimization of ion channel numbers and simulation of whole-cell currents is provided in S2 Text. Individual macroscopic currents of estimated channel numbers in G0 and G1 phase are illustrated in S3–S7 Figs.

When comparing the estimated ion channel numbers between the hyperpolarized and depolarized group the main changes are given in a decrease of ion channels generally responsible for hyperpolarization of the A549 membrane potential, including KCa1.1, KCa3.1 and TASK-1.[66,67,79,80] These changes coincide with the known alterations of ion channel activity and their influence on the membrane potential, and thus confirm the plausibility of the model optimization for both cycle phases. In addition, also the Kv7.1 current is lowered due to the lack of the time-dependent increase of the current, which equally provokes a slight depolarization of the cell membrane. By contrast, the number of Kv3.1 channels and the instantaneous activating current of Kv3.4 channels is rated higher by the model for depolarized than for the hyperpolarized cells, whereby the number of Kv1.3 is marginally decreased by 2 channels and TRPC6 by 5 channels, corresponding to a 34% decrease of the TRPC6 current.

### Model verification based on experimental and synthetic data

**Fig 5:**
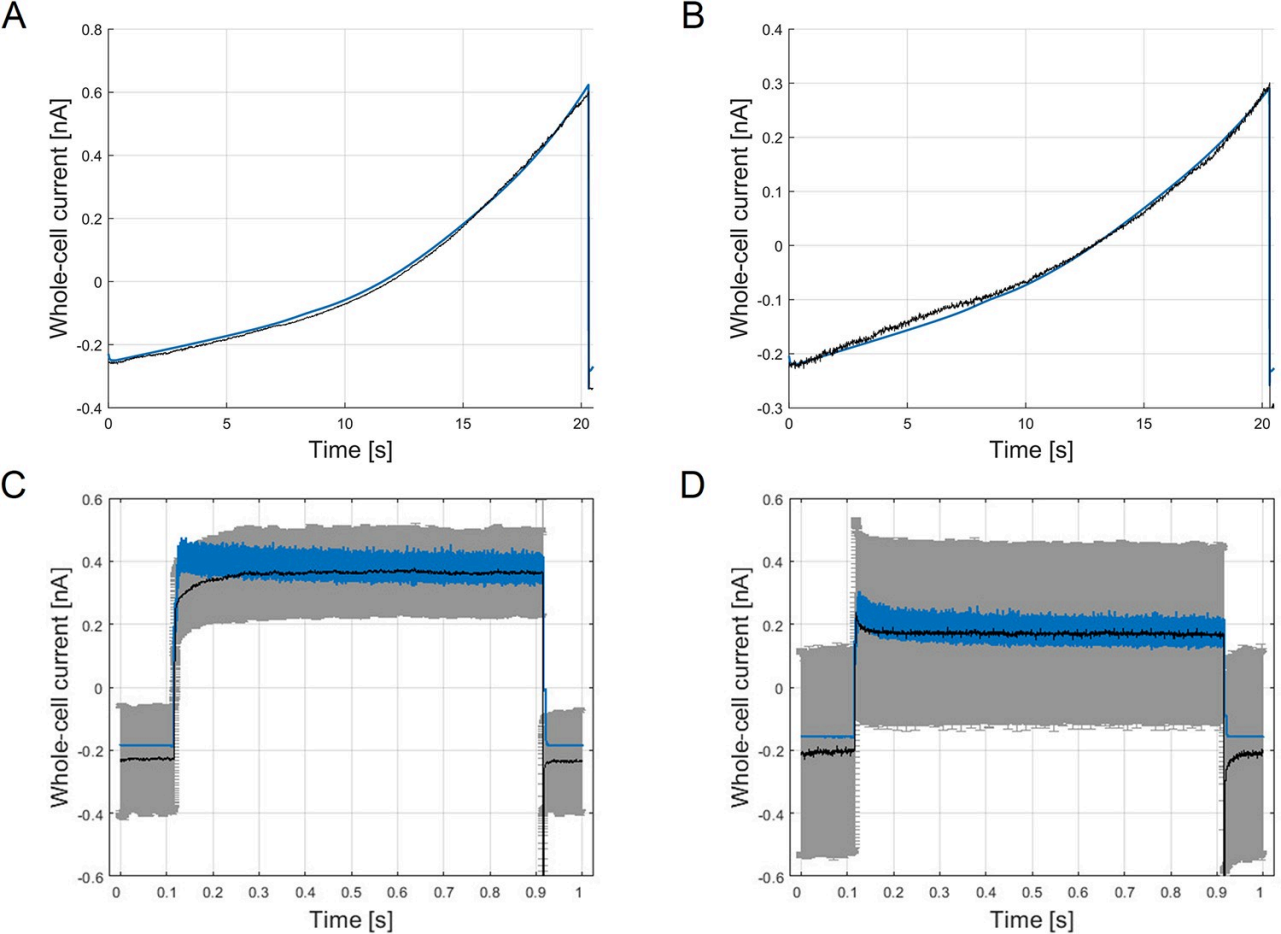
Model verification by experimental and synthetic data. Voltage-ramp currents measured at -100 mV to -40 mV (black lines) for (A) cells in G0 (*n* = 9) and (B) G1 (*n* = 4) phase, and corresponding model simulations (blue lines, *RMSE*_G0_ = 0.0314 and *RMSE*_G1_ = 0.0211). (C, D) Comparison of the whole-cell current of simulated sample cells (blue lines, *n* = 100) with averaged whole-cell current curves from activation protocols (black line, mean ± SD) in G0 and G1 phase.

For model verification voltage-ramp protocols from patch-clamp experiments were simulated and compared with the measured whole-cell currents for cells in G0 (*n* = 9) and G1 phase (*n* = 4). As shown in Fig 5A and 5B, the simulated ramp currents approximate well with the measured currents (*RMSE*_G0_ = 0.0314, *RMSE*_G1_ = 0.0211), confirming the accurate and reliable estimation of ion channel numbers by model optimization. Measured deactivation protocols were also simulated, but not considered further for model verification because of the limited quantity and low quality of experimental data (see S11 Fig).

As an additional verification step, we generated a synthetic data set of 1000 sample cells with the ion channel composition delivered from model optimization. In comparison, the macroscopic channel currents were simulated based on stochastic open probabilities of the individual Markov models (see Methods section) to test whether the expected open probability is a sufficient parameter for model parametrization and whether the model also represents the natural gating behavior in a large population of cells. The simulated whole-cell currents for all sample cells lay within the range of standard deviation of measurements, confirming the validity of this approximation for model optimization. Fig 5C and 5D exemplarily illustrates the simulated whole-cell current of 100 randomly selected sample cells at a voltage-level of +40 mV in G0 and G1 phase. Simulation results of all other voltage levels can be found in S8 and S9 Figs.

### In-silico simulation of the A549 cell cycle

Optical control in performed patch-clamp experiments enabled us to exclude all cells during cell division (G2/mitosis phase). In particular, cells already indicating two nuclei and cells that were greatly enlarged in the antecedent G2 phase could not be patched because of their very weak and unstable cell membranes. Thus, patch-clamp experiments for model parametrization and verification comprise only those cells which were unambiguously in the G0, G1 or S cell cycle phase. According to the rhythmic oscillation of the membrane potential during cell cycle progression, showing a characteristic depolarization in G1 phase and hyperpolarization in G0 and S phase we were able to characterize patched cells being depolarized in G1 phase and hyperpolarized either in G0 or S phase.[19]

**Fig 6:**
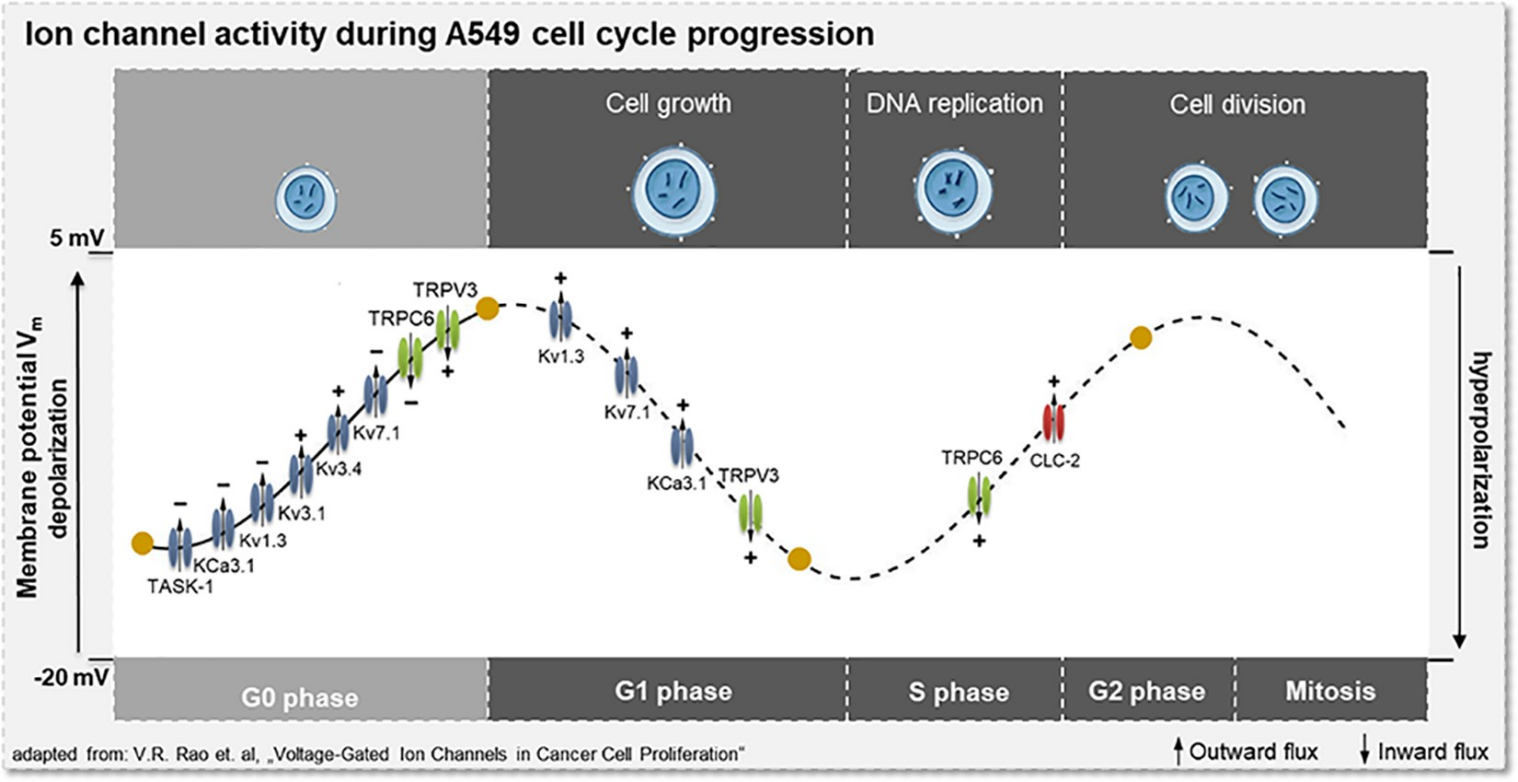
Schematic illustration of the simulated ion channel activity during the A549 cell cycle. G0/G1 transition: modulation of ion channel activity predicted by the model approach. G1/S and S/G2 transition: simulation of ion channel activation and inactivation, responsible for A549 cell cycle progression. Calculated membrane potentials for G0, G1, S and G2 phase are illustrated by yellow dots: *V*_m_G0_ = -10.4 mV, *V*_m_G1_ = -1.33 mV, *V*_m_S_ = -13.3 mV, *V*_m_G2/M_ = -5.49 mV. The symbols + and - indicate activation and inactivation of ion channels.

Since the optimized model fits well with the depolarization of the cell membrane by specific ion channel inactivation (c.f. [66,67,79,80]) demonstrating particularly a reduction of the TASK-1 and hIK current, we can distinguish hyperpolarized cells in the resting G0 phase from those in the further progressed S phase. Fig 6 shows all the channels involved and their changes in activity during the transition from G0 to G1 phase as predicted by the in-silico model.

To estimate and predict membrane potential changes during cell cycle progression, the membrane potential *V*_m_ was calculated by setting Eq 7 to zero:

$$C∙\frac{dV_{m}}{dt}=-I_{whole\_cell}$$ (7)

where

$$I_{\mathrm{whole}\_\mathrm{cell}}=\sum (N_{x}∙P_{Ox_{steady-state}}∙g_{x}∙(V-E_{ion}))$$

Thereby, the potential *V*, for which the whole cell current *I*_whole_cell_ equals zero in steady state condition, represents the membrane potential of the cell.

In addition, the membrane potential *V*_m_ was simulated by numerically solving the differential Eq 7 for *V*_m_, starting at +5 mV until steady state was reached (*t* = 10 s, d*t* = 5.10^−7^). Both approaches led to almost the same membrane potential values and comply with the extracted reversal potentials *V*_rev_ from derived current-voltage curves and performed voltage-ramp measurements as shown in Fig 4. The time course of simulated membrane potentials in the different cell cycle phases is provided in S12 Fig. A comparison of measured and calculated potentials is shown in S3 Table.

Known actuators in G1–S transition of A549 cells are Kv1.3, Kv7.1, hIK and TRPV3 (Fig 6, G1 phase).[66,81–83] In accordance with the literature, we increased the current of these four channels, leading to a hyperpolarization to -13.3 mV, conceivable to trigger S phase initiation. In turn, the transition from S to G2/M phase again requires a depolarization of the membrane potential which is linked to an activation of TRPC6 channels in A549 cells.[84] A further activation by increasing the number of TRPC6 channels in the model results in a depolarization of the membrane potential to -5.49 mV, enabling the triggering of S-G2/M transition in the A549 cell cycle. In addition, we increased the CLC-2 current because of a well-known contribution of the CLC channel activation in S-G2/M phase initiation, although this does not lead to a noticeable increase of the membrane potential to –5.495 mV (Fig 6, G2/M phase).[19] The activation and inactivation of all ion channels involved and so far confirmed from literature and our own experimental data clearly demonstrates the characteristic oscillation of the membrane potential during cell cycle progression by simulations of the A549 in-silico model. The individual channel numbers and membrane potentials calculated for the different cell cycle phases are summarized in Table 2.

**Table 2:** Ion channel numbers and simulated ion channel blockages during different cell cycle phases in the A549 in-silico model.

	G0 phase			G1 phase				S phase			G2/M phase	
A549 ion channels	optimized number of channels	simulated		optimized number of channels	simulated			Adjusted number of channels	simulated		Adjusted number of channels	simulated
		hIK block	TRPC6 block		Kv7.1 block	Kv1.3 block	TASK-1 block		hIK block	Kv1.3 block		TRPC6 block
Kv1.3	22	22	22	20	20	***blocked***	20	45^a^	45	***blocked***	45	45
Kv3.1	78	78	78	90	90	90	90	90	90	90	90	90
Kv3.4	5	5	5	54	54	54	54	54	54	54	54	54
Kv7.1	1350	1350	1350	558	***blocked***	558	558	650^a^	650	650	650	650
KCa1.1	40	40	40	15	15	15	15	15	15	15	15	15
KCa3.1	77	***blocked***	77	63	63	63	63	210^a^	***blocked***	210	210	210
TASK-1	19	19	19	10	10	10	***blocked***	10	10	10	10	10
CRAC	200	200	200	200	200	200	200	200	200	200	200	200
TRPC6	17	17	***blocked***	12	12	12	12	12	12	12	20^a^	***blocked***
TRPV3	12	12	12	13	13	13	13	20^a^	20	20	20	20
CLC-2	13	13	13	11	11	11	11	11	11	11	60^a^	60
***V***_**m**_ **[mV]**	**-10.4**	**-3.96**	**-34.0**	**-1.33**	**+8.7**	**-1.15**	**+1.42**	**-13.3**	**+12.4**	**-13.0**	**-5.49**	**-31.1**
**Δ*V***_**m**_ **[mV]**		**+6.44**	**-23.6**		**+10.0**	**+0.18**	**+2.75**		**+25.7**	**+0.30**		**-25.6**

a) Assumed changes of ion channel numbers correspond qualitatively to alterations of ion channel activity in the different cell cycle phases as described in the literature.

### Model simulation and validation of altered ion channel activity of the A549 cell cycle based on literature data

Modulation of the membrane potential in different cell cycle phases, leading to promotion or interruption of cell cycle progression due to significant hyper- or depolarization, can be provoked by targeted activation and inactivation of ion channels.[19] Model simulation therefore provides an excellent tool for predicting the effects of ion channel activity on cell cycle progression. As a first validation step, we simulated 5 different, experimentally confirmed scenarios of ion channel activation and inactivation over the A549 cell cycle and their impact on the membrane potential and cell cycle progression.

**Fig 7:**
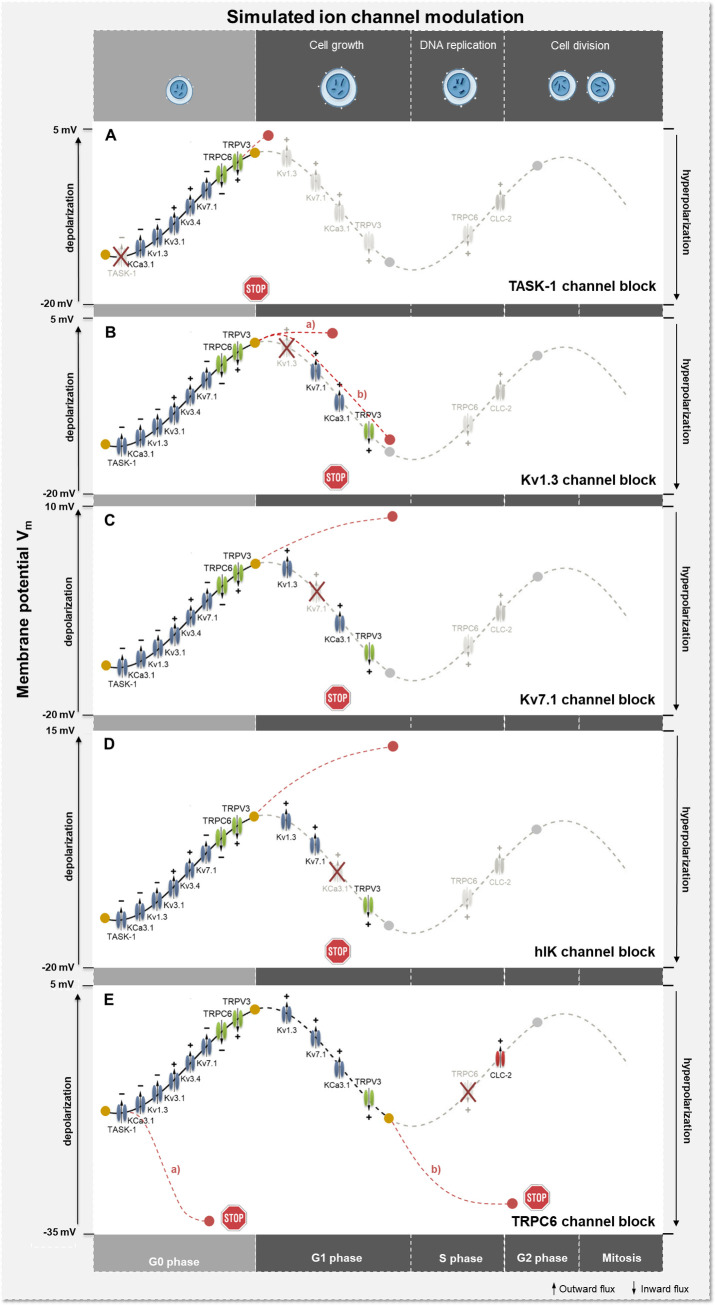
Schematic illustration of model simulations of ion channel activation and inactivation during cell cycle progression. (A) Simulation of TASK-1 channel blockage in G1 phase. (B) Simulation of Kv1.3 channel blockage in a) G1 phase and b) during transition from G1 to S phase. (C) Simulation of Kv7.1 channel blockage starting at G1 phase. (D) Simulation of KCa3.1 channel blockage during G1-S transition. (E) Simulation of TRPC6 channel blockage in a) G0 phase and b) during transition from S to G2/M phase. The symbol + means activation, - means inactivation of ion channels.

A proven A549 cell cycle modulator is the TASK-1 channel. Its inhibition leads to a depolarization of the cell membrane and is related to reduced proliferation, mitosis and enhanced apoptosis.[67] Reduction or even a block of TASK-1 channel activity in G1 phase, as illustrated in Fig 7A, induces a depolarization of the membrane potential up to +1.42 mV, conceivable to arrest cells in G1 phase and thus prevent proliferation.

Inhibition of Kv1.3 channels was shown to be associated with a depolarization of the cell membrane potential, accompanied by cell cycle arrest by impeding G1-S transition in A549 cells. Extending beyond this, recent studies have revealed reduced proliferation and suppressed tumor growth of A549 lung adenocarcinoma in vivo induced by Margatoxin (MgTX), a specific Kv1.3 channel blocker. The anti-proliferative effect of MgTX is related to proteins modulating the cell cycle, i.e. increased expression of the CDk1 inhibitor p21^WAF1/Cip1^ and a decrease of CDk4 and cyclin D3.[65,82] Simulating the inhibition of the Kv1.3 current in the G1 phase results in merely a small additional depolarization of the membrane potential from -1.33 mV to -1.15 mV (Δ*V*_m_ = +0.18 mV) (Fig 7B, course a). Additionally, the blocking of these channels also results in a less negative membrane potential of -13.0 mV (c.f. unblocked state of -13.3 mV, Δ*V*_m_ = +0.30 mV), while the KCa3.1, Kv7.1 and TRPV3 currents for G1-S transition remain unchanged (Fig 7B, course b). This lowered hyperpolarization is probably not sufficient to prevent G1-S transition itself, but the missing Kv1.3 current, respectively the changed membrane potential might affect the expression of cell cycle specific proteins, necessary for initiation of the G1-S transition.

Expression of Kv7.1 (KvLQT1) in A549 cells correlates with wound healing, motility and progression.[81] Blocking of the channels reduces wound healing rates by up to 31%, indicating a decrease of cell motility depending on the blocker concentration.[81] In addition, inhibition of Kv7.1 also significantly interferes with cell cycle progression by decreasing the proportion of cells in S and G2/M phases, resulting in a reduction of cell growth.[81] Simulation of a complete block of Kv7.1 channels in the G1 phase confirms these observations, leading to a significant depolarization up to +8.7 mV (Fig 7C), which is very likely to induce an overall cell cycle arrest.

Activation of hIK channels is known to promote G1/S transition in A549 cells, whereas inhibition increases the proportion of cells in G0/G1 phase. Model simulation of hIK channel inhibition in the G1 phase leads to a strong depolarization of *V*_m_ up to +12.4 mV (Fig 7D). This might suppress the transition from G1 to S phase and thus inhibit further cell cycle progression, resulting in a cell cycle arrest in G1 phase.[66,80,85] Similarly, the simulation of a knockdown or silencing of the channels by the model, this being equivalent to a total lack of hIK current starting in G0 phase, leads to a significant depolarization of the potential (*V*_m_ = -3.96 mV), possibly interrupting further progression. In addition, the depolarization in the G0 phase of Δ*V*_m_ = +6.44 mV correlates with recent experimental data, confirming the estimated ion channel numbers for hIK by model optimization.[66]

TRPC6 channels are highly expressed during S-G2/M transition. Inhibition of these channels results in arrest of the cell cycle and decreased mitosis, invasion and proliferation.[84,86] Simulation of TRPC6 channel block in the S phase leads to a strong hyperpolarization of the estimated membrane potential (*V*_m_ = -31.1 mV), enabling suppression or bypassing of the required depolarization for S-G2/M transition and feasibly interrupting cell cycle and inhibition of mitosis as shown in Fig 7E, course b. By contrast, the knockdown of TRPC6 channels causes a further severe hyperpolarization of the cells in G0 phase (*V*_m_ = -34.0 mV), possibly to prevent the cells from entering into the cell cycle (Fig 7E, course a). The ion channel numbers and corresponding ion channel blockage of each simulation are given in Table 2.

## Discussion

In this work we introduced for the first time an electrophysiological in-silico model of a cancer cell, in particular of the A549 human lung adenocarcinoma epithelial cell, taking into account the most relevant ion channels contributing to the membrane current and resting membrane potential of the cell. Single channel kinetics were modeled by a HMM approach and optimized by fitting the macroscopic currents of various ion channels functionally expressed in A549 cells, using whole-cell patch-clamp experiments. For model optimization, a linear optimization method *(lsqlin* Solver, Mathworks Inc.*)* and two non-linear, global optimization approaches (Simulated Annealing SA, *simulannealbnd* function Mathworks Inc. and Particle Swarm Optimization PSO, *particleswarm* function Mathworks Inc.*)* were used and the results compared. The PSO method showed, in comparison to the other algorithms, the most precise fit and highest stability and reproducibility over 1000 conducted simulation runs and was finally selected for model parametrization. For more information on the different optimization algorithms see S10 Fig.

The model takes into account the specific calcium and voltage dependencies of channel kinetics and enables the simulation and characterization of channel interaction, activation and inhibition, and prediction of membrane potential changes over parts of the cell cycle. The membrane potential of the cell is a fundamental physiological parameter, modulating various basic cellular functions, in particular proliferation of cells through rhythmic oscillation from hyperpolarized to depolarized states. These oscillations are caused by activation and inactivation of individual ion channels and trigger the transition between cell cycle phases [10,16,17,19].

The presented model facilitates a methodologically reliable and physiologically reasonable explanation of ion channel modulations and their impact on the whole-cell current and membrane potential as demonstrated in five cellular mechanisms published on the A549 cell line. The investigated mechanisms could be electrophysiologically explained and confirmed based on the simulated membrane potential changes. Specifically, inhibition of each of the channels Kv1.3, TASK1, KV7.1 and KCa3.1 induces a depolarization of the membrane potential and cell cycle arrest in G1 phase. Consistent with the literature, the cell cycle-specific block of each of these channels in the model in G1 phase provokes a depolarization of the membrane potential, plausibly representing the measured effect on the cell cycle. In comparison, inhibition of TRPC6 and activation of hIK channels results in a hyperpolarization of the cell membrane, impeding the S-G2/M phase transition and arresting the cells in the S phase. Simulation of both, TRPC6 channel block and increased hIK channel activity in S phase, led to a hyperpolarization of the membrane potential, also reliably reflecting the experimental findings from the literature. Beyond this, the absolute change in membrane potential when blocking the hIK channel activity in G0 phase is in accordance with experimental data, confirming the accuracy of optimization and validity of the model at this stage. The model therefore offers a first, valuable approach for investigating the effects of ion channel blockers or activators on the cell cycle and subsequently on cancer progression.

Nevertheless, a comprehensive experimental validation of this initial in-silico model of the A549 cell line is the next essential step. This includes a stepwise functional characterization of each single ion channel considered in the model by combined cell cycle analysis and patch-clamp experiments. Based on these extensive experimental investigations, the model can be further adapted and improved in order to finally provide a validated and powerful in-silico tool for research in cancer electrophysiology.

As in any electrophysiological cell model, the complexity of the biological system and limited experimental data require certain model assumptions and abstractions, which may restrict the accuracy and validity of such a model. In this approach, functionally expressed ion channels such as acid sensitive sodium channels (ASICs), amiloride sensitive sodium channels (ENaCs) and the CFTR chloride channels, regulated by cAMP, are not included in the current model. Also, Kv3.3, Kv9.3 and K2P9.1 channels were excluded because of a lack of available kinetic information. Nevertheless, the model contains all main functional ion current types and their specific kinetics present in A549 cells, comprising the large outward current sustained by potassium channels and, in comparison, the comparatively small inward current by chloride and calcium channels. Above all, however, the involvement of the main current types ensures a reliable and valid electrophysiological cell model, as demonstrated for a number of whole-cell ion-current models of excitable (cardiac or neural) cells at different levels of complexity in the past, allowing precise simulations of the action potential and its modulation, considering only a few summative ion currents [36–38,60].

As stated, current knowledge concerning ion channel expression and its function is limited for the modeled cell line. Indeed, the functional expression of more than 80% of ion channel genes found in A549 (95 of 113 channels) has not been proven yet. Thus, several ion channels were not considered in the current model state, limiting an accurate estimation of the remaining individual ion channel numbers by model optimization. The constraints for estimating the numbers of the respective ion channels in a realistic manner, however, were defined based on the experimental data from previous work and the literature data. Not only expression levels, but also the expression of a channel itself can basically be linked to distinct phases of the cell cycle, which represents a further uncertainty that cannot be assessed in the current state. For instance, a characteristic Cav3.1 current could only be found in 8% of A549 cells, which might be an indication of a cell cycle dependent expression of these channels in the plasma membrane.[87] Presently not considered ion channels as well as cell cycle specific expression levels can be attributed to deviations of the fitted whole-cell currents, in particular in the dynamics of the *instant current* at lower voltage levels (see Fig 3E and 3F), somewhat limiting the predictive power of the model in its current stage.

It is known that the kinetics of ion channels is strongly influenced by the biochemical environment and thus may differ between the different cell types.[88] Markov models available from other, non-excitable and transfected cell types, including HEK293, lymphocytes and oocytes, were used for the model approach which might affect the estimation of ion channel numbers in the model accordingly. In particular, the large number of Kv7.1 channels needed to simulate the time dependent increase of the whole-cell current in the G0 phase can be explained by alterations in the kinetics of the HMM model used, derived from Xenopus oocytes, assuming 70% of the channels in the inactivated state.[47] Similarly, the small deviations of simulated instant currents might be the result of slightly aberrant kinetics in the Markov models used. In addition, some of the parameters, such as single-channel conductivities, the calcium diffusion or transfer coefficient have not yet been quantified in this cell line and therefore needed to be adopted from other cell types respectively estimated for A549 cells. However, with regard to the calcium modeling it is important to note that the estimation of the local calcium concentration in this first model version is based on a simplified approach, assuming a certain steady state concentration which does not account for the complex local calcium dynamics.[61] Nevertheless, the local calcium distribution and time evolution in microdomains of the cell is highly significant for the activity of responding ion channels and, subsequently, affect the entire electric properties of the cell membrane.[89–92] Thus, to overcome this limitation and to further increase the model accuracy, a spatio-temporal modeling approach, addressing the heterogenous calcium profile and dynamics in the ER-PM junction provoked by local calcium release of CRAC channels from the edoplasmatic reticulum needs to be considered in future work of a more advanced model version.

The simulated and calculated membrane potentials *V*_m_ are based on the model parametrization of the measured activation curves, corresponding well to the measured reversal potentials *V*_rev_ from voltage-ramp protocols, but differ partly from the resting membrane potentials *V*_rest_. The latter were directly measured after establishing the whole-cell configuration in current-clamp mode. In general, the measured membrane potentials (e.g. *V*_rest_ and *V*_rev_) are strongly influenced by the experimental conditions. In whole-cell patch-clamp experiments the cytoplasm and the intracellular pipette solution are in an exchange process, which slightly alters the driving force over time and thus the membrane potential determined via the subsequently performed activation and ramp protocols.[93] Despite the convergence to zero, however, the reversal potentials derived from voltage-ramp measurements and the calculated membrane potentials demonstrate a significant difference between the hyperpolarized and depolarized cell groups in G0 vs. G1 phase. This difference is sufficient to confirm the possibility of membrane potential changes and their directions of change.

In summary, despite of these limitations, the introduced A549 model 1.0 allows a precise and reliable simulation of ion channel mediated alterations of the membrane potential in different cell cycle phases, serving as a first in-silico tool that supports the understanding of cancer electrophysiology in the human lung adenocarcinoma. Integration of continuously growing knowledge of ion channel expression and function in the A549 cell, together with further experimental investigations of ion channel expression, kinetics and function, and membrane potential changes over the A549 cell cycle will further increase the validity and predictive power of the model. We believe that this pioneering work may serve as a starting point for more advanced models in cancer electrophysiology taking into account additional biological, biochemical and electrophysiological information. Hodgkin-Huxley introduced the first mathematical whole-cell model that described the ionic mechanisms underlying the initiation and propagation of action potentials in an excitable nerve cell in 1952. Now almost 70 years later we have a first electrophysiological model of a cancer cell in hand, with the potential to usher in a new era of computational cancer electrophysiology.

## Methods

### A549 cell line

A549 human lung epithelial carcinoma cells, initiated from male lung carcinomatous tissue, were provided by the Center for Medical Research (ZMF) (Medical University Graz, Austria) obtained from the American Type Culture Collection (ATCC) and cultivated in Dulbecco’s Modified Eagle Medium (DMEM) supplemented with 10% fetal bovine serum (FBS) and 1% penicillin-streptomycin. Cells were maintained at standard conditions in an incubator, 37°C, 5% CO_2_ in humidified atmosphere. The cells were split using Trypsin/EDTA every other day at confluence level of 50% to 70%.

### Electrophysiological recordings and quality assessment

A549 cells (passage 6 to 8) were seeded on 6x6 mm coverslips in 6 well plates and cultured as described for 24h to 48h before electrophysiological recordings. Patch-clamp measurements were performed using the Axopatch-200B amplifier (Molecular Devices, USA) equipped with an inverted microscope (IM35, Zeiss, Germany) and Scientifica PatchStar micromanipulator. Patch-clamp pipettes were pulled from borosilicate glass capillaries (Assistant Mikro-Haematokrit capillary tubes, Hecht, Fisher Scientific) with a final resistance of 1.8 to 2.5 MΩ.

The external bath solution consisted of (in mM) 5.4 KCl, 137 NaCl, 5.6 d(+)-Glucose.H_2_O, 2.2 NaHCO_3_, 1.1 MgCl_2_.6H_2_O, 0.4 NaH_2_PO_4_.H2O, 10 HEPES/Na^+^, 1.8 CaCl_2_.2H_2_O, buffered with 2M NaOH to pH: 7.4. The intracellular solution contained (in mM) 4.3 ATP/K^+^, 110 KCl, 11 EGTA/Mg^2+^, buffered with KOH to pH: 7.4.

Resting membrane potentials were measured immediately after establishing the whole-cell configuration under current-clamp for 10 s and the entire trace averaged using Clampfit 10.3 software (Molecular Devices, USA). The resting membrane potential was low pass filtered at 50 Hz and traces digitized at 1 kHz using the Digidata 1322 A interface (Molecular Devices, USA) and Clampex 9.2 software (Molecular Devices, USA). Whole-cell currents were measured under voltage-clamp with a pulse protocol consisting of an initial- and re-pulse of -80 mV for 100 ms and 800 ms long test pulses from -40 mV to +40 mV (increment 10 mV). Voltage-ramp protocols for determining the reversal potential and model verification were performed starting from -100 mV to +60 mV in 20 s. The applied deactivation protocol consisted of an initial- and re-pulse of -40 mV for 150 ms, a depolarization pulse at 40 mV over 5 s for activation followed by seven 5 s long deactivation pulses from -40 mV to -100 mV (increment 10 mV). Currents were digitized with a sampling rate of 20 kHz and filtered at 2 kHz (Bessel). The holding potential was set to -100 mV between all recordings. Measurements were performed at room temperature between 22°C and 24°C.

To ensure a stable cell condition and reliable results, the experiments considered for data analysis were limited to a test time no longer than 20 minutes after removing cells from the incubator. The quality criteria for recordings are met showing a seal resistance at least greater than 1 GΩ, access resistance below 20 MΩ and a membrane capacitance greater than 20 pF. Cell capacitance was estimated according to a standard protocol from the patch-clamp system using the membrane test. In a data pre-processing step the capacitive peak of the current curves at the beginning of the test pulse of whole-cell recordings considered for data analysis and simulation were eliminated.

### Computational modeling

The A549 model was implemented using the simulation environment MATLAB (R2019b, Mathworks Inc.). Macroscopic currents of single ion channels were modeled by hidden Markov models (HMMs) and the number of ion channels were optimized by fitting the macroscopic current to the measured whole-cell currents by a particle swarm optimization (PSO) algorithm (swarm size: 500, number of iterations: 5000, function tolerance: 1.10^−6^) using the Global Optimization Toolbox (Mathworks Inc.). In order to obtain a more precise optimization and, if possible, to ensure a global solution, we additionally used the *fmincon* solver as hybrid function after PSO optimization. The constraints for optimization were gradually restricted further and the solver was run again until the function value (*fval*) finally reached a constant value (*fval* = 1.5412.10^−17^) over all simulation runs, indicating a stable solution of the estimated ion channel numbers. The individual Markov models and corresponding rate constants of ion channels used in the A549 whole-cell current model are denoted in S1 Fig and S1 Table. In accordance with the applied voltage-step protocol in patch-clamp measurements, a corresponding pulse protocol was implemented for simulations. To consider the holding potential between the single measurements and to bring the channels into a defined initial state, we added an additional initial pulse of -100 mV for 100 ms in the pulse protocol for simulations (see S1A Fig). All hidden Markov models were simulated with a sampling frequency of 2000 kHz (step size d*t* = 5.10^−7^).

The matrix below exemplarily shows the state model of the Kv7.1 channel for simulating the expected open probability of the channels for model parametrization. The vector $P_{k}={\left[P_{C_{1},k}\;P_{C_{2},k}\;P_{O_{1},k}\;P_{O_{2},k}\;P_{I,k}\right]}^{T}$, representing the fraction of channels in each of the states *S* = {*C*_1_, *C*_2_, *O*_1_, *O*_2_, *I*}, was updated for each time step with the *lsim* function (Mathworks Inc.) for all voltage levels over the entire simulation protocol. Based on the expected open probabilities ($P_{O_{1},k}$ and $P_{O_{2},k}$), the number of ion channels ($N_{c_{x}}$) was optimized by fitting the resulting individual macroscopic current to the measured whole-cell current.


$$\left[\begin{array}{c} P_{C_{1},k+1}\\ P_{C_{2},k+1}\\ P_{O_{1},k+1}\\ P_{O_{2},k+1}\\ P_{I,k+1} \end{array}\right]=\left[\begin{array}{ccccc} 1-\alpha ∙dt & \beta ∙dt & 0 & 0 & 0\\ \alpha ∙dt & 1-(a+\beta )∙dt & b∙dt & 0 & 0\\ 0 & a∙dt & 1-(b+c)∙dt & d∙dt & 0\\ 0 & 0 & c∙dt & 1-(d+\eta )∙dt & \lambda ∙dt\\ 0 & 0 & 0 & \eta ∙dt & 1-\lambda ∙dt \end{array}\right]∙\left[\begin{array}{c} P_{C_{1},k}\\ P_{C_{2},k}\\ P_{O_{1},k}\\ P_{O_{2},k}\\ P_{I,k} \end{array}\right]$$

$$P_{O,k}=P_{O_{1},k}+P_{O_{2},k}$$ (8)

$$I_{macro,k}=N_{c_{x}}∙{P_{O,k}∙g}_{x}∙(V-E_{x})x\ldots Kv7.1$$ (9)

The expected single channel currents for simulation and optimization of the macroscopic currents are illustrated in S1 Fig.

To model the stochastic opening and closing of the single ion channels in order to simulate the current of the sample cells, the *hmmgenerate* function (Mathworks Inc.) was used to generate a random sequence of states, depicting the single channel activity based on the Markov model. The transition probability matrix T for the Kv7.1 Markov model is defined as follows:

$$T=\left[\begin{array}{cccccc} & C1 & C2 & O1 & O2 & I\\ C1 & 1-\alpha ∙dt & \alpha ∙dt & 0 & 0 & 0\\ C2 & \beta ∙dt & 1-(a+\beta )∙dt & a∙dt & 0 & 0\\ O1 & 0 & b∙dt & 1-(b+c)∙dt & c∙dt & 0\\ O2 & 0 & 0 & d∙dt & 1-(d+\eta )∙dt & \eta ∙dt\\ I & 0 & 0 & 0 & \lambda ∙dt & 1-\lambda ∙dt \end{array}\right]$$

States corresponding to the channel being open (states 3 and 4) are summarized over all individual channels at each time step, constituting the number of channels used for calculating the macroscopic current according to Eq 3.

The source code for simulation of HMMs and model evaluation is provided in S2 Text.

### Statistical analysis

Measurements of *n* = 16 of originally *n* = 50 individual A549 cells fulfilled the quality standards of our lab and were considered for data analysis. The cells were subdivided in two groups representing cells in the G0 phase (*n* = 11) and G1 phase (*n* = 5). Model optimization is based on the averaged measured whole-cell currents of the two cell groups. Mean values and standard deviations ($\bar{x}\pm \sigma$) of corresponding steady state currents at all voltage-levels are shown in Fig 3C and 3D. Normal distribution of measured resting potentials, ramp potentials and reversal potentials were tested using the Shapiro Wilk and the Kolmogorow-Smirnow tests. Statistical significance of measured resting potentials between cells in G0 phase and G1 phase was tested by using a two-tailed Student-t test. Statistical significance of reversal potentials from voltage-ramp measurements and current-voltage curves between the two groups was tested with the Mann-Whitney-U test. A p-value below 0.05 was considered significant (see Fig 4). All statistical analyses were performed using MATLAB (MathWorks Inc.) and results are summarized in S2 Table.

## Supporting information

S1 Text Literature review of ion channels in the A549 cell line. (DOCX)

S2 Text Source code A549 in-silico model. (DOCX)

S1 Table Parameters of hidden Markov models used in the A549 whole-cell current model. Rate constants for transitions between the states of all hidden Markov models. For calcium dependent transitions the calculated steady state calcium concentration *Ca_i* = 4.68 μM is used.
(DOCX)

S2 Table Patch-clamp results and statistical analysis. Resting potentials of current-clamp measurements, reversal potentials from voltage-ramp measurements (ramp potential) and reversal potentials derived from current-voltage curves (reversal potential) of the individual cells with negative resting potential in G0 and positive resting potential in G1 phase.
(DOCX)

S3 Table Comparison of measured and calculated membrane potentials in the different cell cycle phases. (DOCX)

S1 Fig Kinetic schemes and expected single channel currents of implemented ion channels. (A) Voltage-step protocol of patch-clamp measurements and below the corresponding protocol for model simulation. Kinetic scheme and expected single channel currents for voltage levels between -40 mV to +40 mV of the ion channels (B) Kv1.3, (C) Kv3.1, (D) TRPV3, (E) Kv3.4, (F) Kv7.1, (G) TRPC6, (H) TASK-1, (I) CRACM1, (J) CLC-2, (K) KCa1.1 and (L) KCa3.1.
(TIF)

S2 Fig Time evolution of the local calcium concentration. Simulated changes of the local calcium concentration provoked by CRAC channels at holding potential of -100 mV over 10 s. Starting point c[Ca^2+^]_i_ = 0.0647 μM, steady state c[Ca^2+^]_i_ = 4.6847 μM, *e*_trans_ = 21.8976∙10^−3^ μMpA^-1^ms^-1^L^-1^, *e*_diff_ = 3∙10^−3^ ms^-1^.
(TIF)

S3 Fig Macroscopic currents of single ion channels in G0 and G1 phase. Macroscopic currents of single ion channels, estimated by model optimization in respect to whole-cell current for A: G0 phase and B: G1 phase at +40 mV.
(TIF)

S4 Fig Comparison of macroscopic currents of voltage-gated potassium channels in G0 and G1 phase. Comparison of macroscopic currents of voltage-gated potassium channels Kv1.3, Kv3.1, Kv3.4 and Kv7.1 in G0 and G1 phase at voltage levels from -40 mV to +40 mV.
(TIF)

S5 Fig Comparison of macroscopic currents of potassium channels in G0 and G1 phase. Comparison of macroscopic currents of potassium channels TASK-1, KCa1.1 and KCa3.1 in G0 and G1 phase at voltage levels from -40 mV to +40 mV.
(TIF)

S6 Fig Comparison of macroscopic currents of calcium channels in G0 and G1 phase. Comparison of macroscopic currents of calcium channels TRPC6, TRPV3 and CRAC in G0 and G1 phase at voltage levels from -40 mV to +40 mV.
(TIF)

S7 Fig Comparison of the macroscopic CLC-2 chloride current in G0 and G1 phase. Comparison of the macroscopic CLC-2 chloride current in G0 and G1 phase at voltage levels from -40 mV to +40 mV.
(TIF)

S8 Fig Simulated whole-cell current of sample cells for G0 phase. Simulated whole-cell current of 100 sample cells at (A) -40 mV, (B) -30 mV, (C) -20 mV, (D) -10 mV, (E) 0 mV, (F) +10 mV, (G) +20 mV, (H) +30 mV, (I) +40 mV for G0 phase. Black lines show the averaged measured whole-cell cell currents, background indicates the corresponding standard deviations at all voltage levels.
(TIF)

S9 Fig Simulated whole-cell current of sample cells for G1 phase. Simulated whole-cell current of 100 sample cells at (A) -40 mV, (B) -30 mV, (C) -20 mV, (D) -10 mV, (E) 0 mV, (F) +10 mV, (G) +20 mV, (H) +30 mV, (I) +40 mV for G1 phase. Black curves represent the averaged whole-cell currents, grey background indicates the corresponding standard deviations at all voltage levels.
(TIF)

S10 Fig Fitting results of the different optimization algorithms applied. Fitting results (n = 100 simulation runs) of (A, B) lsqlin, (C, D) SA and (E, F) PSO for averaged whole-cell currents of cells in G0 and G1 phase.
(TIF)

S11 Fig Simulated deactivation curves. Simulation of the deactivation protocols for (A) G0 (*n* = 5, *RMSE*_G0_ = 0.0754) and (B) G1 (*n* = 3, *RMSE*_G1_ = 0.0673) phase. Deviations can be explained by the small sample size and apparent leakage currents for strongly negative deactivation pulses below -80 mV.
(TIF)

S12 Fig Simulated membrane potential in different cell cycle phases. Simulated membrane potential (starting point at 5 mV over 10 s (d*t* = 5.10^−7^)) for (A) G0 phase *V*_m_ = -10.398 mV, (B) G1 phase *V*_m_ = -1.258 mV, (C) S phase *V*_m_ = -13.2 and mV (D) G2/M phase *V*_m_ = -5.263 mV.
(TIF)

## References

[1] Siegel RL, Miller KD, Jemal A. Cancer statistics, 2018. CA Cancer J Clin. 2018;68: 7–30. doi: 10.3322/caac.21442 29313949

[2] Oser MG, Niederst MJ, Sequist LV, Engelman JA. Transformation from non-small-cell lung cancer to small-cell lung cancer: molecular drivers and cells of origin. Lancet Oncol. 2015;16: e165–e172. doi: 10.1016/S1470-2045(14)71180-5 25846096 PMC4470698

[3] Ma X, Yu H. Global burden of cancer. Yale J Biol Med. 2006;79: 85–94. 17940618 PMC1994799

[4] Cooper WA, Lam DCL, O’Toole SA, Minna JD. Molecular biology of lung cancer. J Thorac Dis. 2013;5: S479–S490. doi: 10.3978/j.issn.2072-1439.2013.08.03 24163741 PMC3804875

[5] The Cancer Genome Atlas Research Network. Comprehensive molecular profiling of lung adenocarcinoma. Nature. 2014;511: 543–550. doi: 10.1038/nature13385 25079552 PMC4231481

[6] Bai Y, Liu X, Qi X, Liu X, Peng F, Li H, et al. PDIA6 modulates apoptosis and autophagy of non-small cell lung cancer cells via the MAP4K1/JNK signaling pathway. EBioMedicine. 2019 Apr;42:311–325. doi: 10.1016/j.ebiom.2019.03.045 Epub 2019 Mar 25. ; PMCID: PMC6491656. 30922965 PMC6491656

[7] Manegold C, Thatcher N. Survival improvement in thoracic cancer: progress from the last decade and beyond. Lung Cancer. 2007;57 Suppl 2: S3–5. doi: 10.1016/S0169-5002(07)70420-8 17686443

[8] Leanza L, Managò A, Zoratti M, Gulbins E, Szabo I. Pharmacological targeting of ion channels for cancer therapy: In vivo evidences. Biochim Biophys Acta. 2016;1863: 1385–1397. doi: 10.1016/j.bbamcr.2015.11.032 Epub 2015 Nov 30. . 26658642

[9] Lastraioli E, Iorio J, Arcangeli A. Ion channel expression as promising cancer biomarker. Biochim Biophys Acta. 2015;1848: 2685–2702. doi: 10.1016/j.bbamem.2014.12.016 25542783

[10] Brackenbury WJ. Chapter 6—Ion Channels in Cancer. In: Ion Channels in Health and Disease. Academic Press; 2016. pp. 131–163. doi: 10.1016/B978-0-12-802002-9.00006–6

[11] Litan A, Langhans SA. Cancer as a channelopathy: ion channels and pumps in tumor development and progression. Front Cell Neurosci. 2015;9: 86. doi: 10.3389/fncel.2015.00086 25852478 PMC4362317

[12] Schönherr R. Clinical relevance of ion channels for diagnosis and therapy of cancer. J Membr Biol. 2005;205: 175–184. doi: 10.1007/s00232-005-0782-3 16362505

[13] Li M, Xiong ZG. Ion channels as targets for cancer therapy. Int J Physiol Pathophysiol Pharmacol. 2011;3: 156–166. Epub 2011 Jun 27. ; PMCID: PMC3134009. 21760973 PMC3134009

[14] Kunzelmann K. Ion channels and cancer. J Membr Biol. 2005;205: 159–173. doi: 10.1007/s00232-005-0781-4 16362504

[15] Fiske JL, Fomin VP, Brown ML, Duncan RL, Sikes RA. Voltage-sensitive ion channels and cancer. Cancer Metastasis Rev. 2006;25: 493–500. doi: 10.1007/s10555-006-9017-z 17111226

[16] Yang M, Brackenbury WJ. Membrane potential and cancer progression. Front Physiol. 2013;4. doi: 10.3389/fphys.2013.00185 23882223 PMC3713347

[17] Blackiston DJ, McLaughlin KA, Levin M. Bioelectric controls of cell proliferation: ion channels, membrane voltage and the cell cycle. Cell Cycle. 2009;8: 3527–3536. doi: 10.4161/cc.8.21.9888 19823012 PMC2862582

[18] Levin M. Molecular bioelectricity in developmental biology: new tools and recent discoveries: control of cell behavior and pattern formation by transmembrane potential gradients. Bioessays. 2012;34: 205–217. doi: 10.1002/bies.201100136 22237730 PMC3430077

[19] Rao VR, Perez-Neut M, Kaja S, Gentile S. Voltage-Gated Ion Channels in Cancer Cell Proliferation. Cancers (Basel). 2015;7: 849–875. doi: 10.3390/cancers7020813 26010603 PMC4491688

[20] O’Grady SM, Lee SY. Molecular diversity and function of voltage-gated (Kv) potassium channels in epithelial cells. Int J Biochem Cell Biol. 2005;37: 1578–1594. doi: 10.1016/j.biocel.2005.04.002 15882958

[21] Binggeli R, Weinstein RC. Membrane potentials and sodium channels: hypotheses for growth regulation and cancer formation based on changes in sodium channels and gap junctions. J Theor Biol. 1986;123: 377–401. doi: 10.1016/s0022-5193(86)80209-0 2443763

[22] Pardo LA, Stühmer W. The roles of K(+) channels in cancer. Nat Rev Cancer. 2014;14: 39–48. doi: 10.1038/nrc3635 24336491

[23] Pardo LA, Contreras-Jurado C, Zientkowska M, Alves F, Stühmer W. Role of voltage-gated potassium channels in cancer. J Membr Biol. 2005;205: 115–124. doi: 10.1007/s00232-005-0776-1 16362499

[24] Becchetti A. Ion channels and transporters in cancer. 1. Ion channels and cell proliferation in cancer. Am J Physiol Cell Physiol. 2011;301: C255–C265. doi: 10.1152/ajpcell.00047.2011 21430288

[25] Fraser SP, Grimes JA, Djamgoz MB. Effects of voltage-gated ion channel modulators on rat prostatic cancer cell proliferation: comparison of strongly and weakly metastatic cell lines. Prostate. 2000;44: 61–76. doi: 10.1002/1097-0045(20000615)44:1&lt;61::aid-pros9&gt;3.0.co;2-3 10861759

[26] Pardo LA, del Camino D, Sánchez A, Alves F, Brüggemann A, Beckh S, et al. Oncogenic potential of EAG K(+) channels. EMBO J. 1999;18: 5540–5547. doi: 10.1093/emboj/18.20.5540 10523298 PMC1171622

[27] Urrego D, Tomczak AP, Zahed F, Stühmer W, Pardo LA. Potassium channels in cell cycle and cell proliferation. Philos Trans R Soc Lond B Biol Sci. 2014;369. doi: 10.1098/rstb.2013.0094 24493742 PMC3917348

[28] Woodfork KA, Wonderlin WF, Peterson VA, Strobl JS. Inhibition of ATP-sensitive potassium channels causes reversible cell-cycle arrest of human breast cancer cells in tissue culture. J Cell Physiol. 1995;162: 163–171. doi: 10.1002/jcp.1041620202 7822427

[29] Weaver AK, Liu X, Sontheimer H. Role for calcium-activated potassium channels (BK) in growth control of human malignant glioma cells. J Neurosci Res. 2004;78: 224–234. doi: 10.1002/jnr.20240 15378515 PMC2561220

[30] Fraser SP, Pardo LA. Ion channels: functional expression and therapeutic potential in cancer. Colloquium on Ion Channels and Cancer. EMBO Rep. 2008;9: 512–515. doi: 10.1038/embor.2008.75 18451877 PMC2427390

[31] Lehen’kyi V, Shapovalov G, Skryma R, Prevarskaya N. Ion channnels and transporters in cancer. 5. Ion channels in control of cancer and cell apoptosis. Am J Physiol, Cell Physiol. 2011;301: C1281–1289. doi: 10.1152/ajpcell.00249.2011 21940667

[32] Downie BR, Sánchez A, Knötgen H, Contreras-Jurado C, Gymnopoulos M, Weber C, et al. Eag1 expression interferes with hypoxia homeostasis and induces angiogenesis in tumors. J Biol Chem. 2008;283: 36234–36240. doi: 10.1074/jbc.M801830200 18927085 PMC2606018

[33] Crociani O, Zanieri F, Pillozzi S, Lastraioli E, Stefanini M, Fiore A, et al. hERG1 channels modulate integrin signaling to trigger angiogenesis and tumor progression in colorectal cancer. Sci Rep. 2013;3: 3308. doi: 10.1038/srep03308 24270902 PMC3839040

[34] Prevarskaya N, Skryma R, Shuba Y. Ion channels and the hallmarks of cancer. Trends Mol Med. 2010;16: 107–121. doi: 10.1016/j.molmed.2010.01.005 20167536

[35] Fraser SP, Diss JKJ, Chioni A-M, Mycielska ME, Pan H, Yamaci RF, et al. Voltage-Gated Sodium Channel Expression and Potentiation of Human Breast Cancer Metastasis. Clin Cancer Res. 2005;11: 5381–5389. doi: 10.1158/1078-0432.CCR-05-0327 16061851

[36] Hodgkin AL, Huxley AF. A quantitative description of membrane current and its application to conduction and excitation in nerve. J Physiol. 1952;117: 500–544. doi: 10.1113/jphysiol.1952.sp004764 12991237 PMC1392413

[37] ten Tusscher KH, Noble D, Noble PJ, Panfilov AV. A model for human ventricular tissue. Am J Physiol Heart Circ Physiol. 2004;286: H1573–1589. doi: 10.1152/ajpheart.00794.2003 14656705

[38] Luo CH, Rudy Y. A model of the ventricular cardiac action potential. Depolarization, repolarization, and their interaction. Circ Res. 1991;68: 1501–1526. doi: 10.1161/01.res.68.6.1501 1709839

[39] Hou P, Zhang R, Liu Y, Feng J, Wang W, Wu Y, et al. Physiological role of Kv1.3 channel in T lymphocyte cell investigated quantitatively by kinetic modeling. PLoS ONE. 2014;9: e89975. doi: 10.1371/journal.pone.0089975 24594979 PMC3940720

[40] Schmeitz C, Hernandez-Vargas EA, Fliegert R, Guse AH, Meyer-Hermann M. A mathematical model of T lymphocyte calcium dynamics derived from single transmembrane protein properties. Front Immunol. 2013;4: 277. doi: 10.3389/fimmu.2013.00277 24065966 PMC3776162

[41] Eichinger P, Herrmann AM, Ruck T, Herty M, Gola L, Kovac S, et al. Human T cells in silico: Modelling dynamic intracellular calcium and its influence on cellular electrophysiology. J Immunol Methods. 2018;461: 78–84. doi: 10.1016/j.jim.2018.06.020 30158076

[42] Ehling P, Meuth P, Eichinger P, Herrmann AM, Bittner S, Pawlowski M, et al. Human T cells in silico: Modelling their electrophysiological behaviour in health and disease. J Theor Biol. 2016;404: 236–250. doi: 10.1016/j.jtbi.2016.06.001 27288542

[43] Giard DJ, Aaronson SA, Todaro GJ, Arnstein P, Kersey JH, Dosik H, et al. In vitro cultivation of human tumors: establishment of cell lines derived from a series of solid tumors. J Natl Cancer Inst. 1973;51: 1417–1423. doi: 10.1093/jnci/51.5.1417 4357758

[44] Foster KA, Oster CG, Mayer MM, Avery ML, Audus KL. Characterization of the A549 cell line as a type II pulmonary epithelial cell model for drug metabolism. Exp Cell Res. 1998;243: 359–366. doi: 10.1006/excr.1998.4172 9743595

[45] Swain RJ, Kemp SJ, Goldstraw P, Tetley TD, Stevens MM. Assessment of cell line models of primary human cells by Raman spectral phenotyping. Biophys J. 2010;98: 1703–1711. doi: 10.1016/j.bpj.2009.12.4289 20409492 PMC2856139

[46] Armstrong JF, Faccenda E, Harding SD, Pawson AJ, Southan C, Sharman JL, et al. The IUPHAR/BPS Guide to PHARMACOLOGY in 2020: extending immunopharmacology content and introducing the IUPHAR/MMV Guide to MALARIA PHARMACOLOGY. Nucleic Acids Res. 2020;48: D1006–D1021. doi: 10.1093/nar/gkz951 31691834 PMC7145572

[47] Pusch M, Magrassi R, Wollnik B, Conti F. Activation and inactivation of homomeric KvLQT1 potassium channels. Biophys J. 1998;75: 785–792. doi: 10.1016/S0006-3495(98)77568-X 9675180 PMC1299753

[48] Destexhe A, Huguenard JR. Modeling Voltage-Dependent Channels. In: Computational Modeling Methods for Neuroscientists. The MIT Press; 2009. doi: 10.7551/mitpress/9780262013277.003.0006

[49] Wang W, Luo J, Hou P, Yang Y, Xiao F, Yuchi M, et al. Native gating behavior of ion channels in neurons with null-deviation modeling. PLoS ONE. 2013;8: e77105. doi: 10.1371/journal.pone.0077105 24204745 PMC3808363

[50] Fineberg JD, Ritter DM, Covarrubias M. Modeling-independent elucidation of inactivation pathways in recombinant and native A-type Kv channels. J Gen Physiol. 2012;140: 513–527. doi: 10.1085/jgp.201210869 23109714 PMC3483116

[51] de Santiago JA, Nehrke K, Arreola J. Quantitative analysis of the voltage-dependent gating of mouse parotid ClC-2 chloride channel. J Gen Physiol. 2005;126: 591–603. doi: 10.1085/jgp.200509310 16286506 PMC2266594

[52] Wang W, Xiao F, Zeng X, Yao J, Yuchi M, Ding J. Optimal estimation of ion-channel kinetics from macroscopic currents. PLoS ONE. 2012;7: e35208. doi: 10.1371/journal.pone.0035208 22536358 PMC3335051

[53] Limberg SH, Netter MF, Rolfes C, Rinné S, Schlichthörl G, Zuzarte M, et al. TASK-1 channels may modulate action potential duration of human atrial cardiomyocytes. Cell Physiol Biochem. 2011;28: 613–624. doi: 10.1159/000335757 22178873 PMC3709183

[54] Bailey MA, Grabe M, Devor DC. Characterization of the PCMBS-dependent modification of KCa3.1 channel gating. J Gen Physiol. 2010;136: 367–387. doi: 10.1085/jgp.201010430 20837673 PMC2947057

[55] Fomina AF, Fanger CM, Kozak JA, Cahalan MD. Single Channel Properties and Regulated Expression of Ca2+ Release-Activated Ca2+ (CRAC) Channels in Human T Cells. J Cell Biol. 2000;150: 1435–1444. doi: 10.1083/jcb.150.6.1435 10995447 PMC2150694

[56] Padar S, van Breemen C, Thomas DW, Uchizono JA, Livesey JC, Rahimian R. Differential regulation of calcium homeostasis in adenocarcinoma cell line A549 and its Taxol-resistant subclone. Br J Pharmacol. 2004;142: 305–316. doi: 10.1038/sj.bjp.0705755 15066902 PMC1574945

[57] Grissmer S, Nguyen AN, Cahalan MD. Calcium-activated potassium channels in resting and activated human T lymphocytes. Expression levels, calcium dependence, ion selectivity, and pharmacology. J Gen Physiol. 1993;102: 601–630. doi: 10.1085/jgp.102.4.601 7505804 PMC2229173

[58] Ferreira R, Schlichter LC. Selective Activation of KCa3.1 and CRAC Channels by P2Y2 Receptors Promotes Ca2+ Signaling, Store Refilling and Migration of Rat Microglial Cells. PLoS One. 2013;8. doi: 10.1371/journal.pone.0062345 23620825 PMC3631179

[59] Christophersen P, Wulff H. Pharmacological gating modulation of small- and intermediate-conductance Ca2+-activated K+ channels (KCa2.x and KCa3.1). Channels (Austin). 2015;9: 336–343. doi: 10.1080/19336950.2015.1071748 26217968 PMC4850045

[60] Beeler GW, Reuter H. Reconstruction of the action potential of ventricular myocardial fibres. J Physiol. 1977;268: 177–210. doi: 10.1113/jphysiol.1977.sp011853 874889 PMC1283659

[61] Ekeberg O, Wallén P, Lansner A, Tråvén H, Brodin L, Grillner S. A computer based model for realistic simulations of neural networks. I. The single neuron and synaptic interaction. Biol Cybern. 1991;65: 81–90. doi: 10.1007/BF00202382 1912005

[62] Wang K, Zhao Y, Chen D, Fan B, Lu Y, Chen L, et al. Specific membrane capacitance, cytoplasm conductivity and instantaneous Young’s modulus of single tumour cells. Sci Data. 2017;4: 170015. doi: 10.1038/sdata.2017.15 28195578 PMC5308201

[63] Gudlur A, Hogan PG. The STIM-Orai Pathway: Orai, the Pore-Forming Subunit of the CRAC Channel. Adv Exp Med Biol. 2017;993: 39–57. doi: 10.1007/978-3-319-57732-6_3 28900908 PMC5764705

[64] Chang Xiao-Wen, Han Qing. Solving Box-Constrained Integer Least Squares Problems. IEEE Trans Wireless Commun. 2008;7: 277–287. doi: 10.1109/TWC.2008.060497

[65] Roth B. Exposure to sparsely and densely ionizing irradiation results in an immediate activation of K+ channels in A549 cells and in human peripheral blood lymphocytes. PhD thesis. Technische Universität Darmstadt. 2014.

[66] Roth B, Gibhardt CS, Becker P, Gebhardt M, Knoop J, Fournier C, et al. Low-dose photon irradiation alters cell differentiation via activation of hIK channels. Pflugers Arch. 2015;467: 1835–1849. doi: 10.1007/s00424-014-1601-4 25277267

[67] Leithner K, Hirschmugl B, Li Y, Tang B, Papp R, Nagaraj C, et al. TASK-1 Regulates Apoptosis and Proliferation in a Subset of Non-Small Cell Lung Cancers. PLoS One. 2016;11. doi: 10.1371/journal.pone.0157453 27294516 PMC4905626

[68] Kerschbaum HH, Cahalan MD. Single-Channel Recording of a Store-Operated Ca2+ Channel in Jurkat T Lymphocytes. Science. 1999;283: 836–839. doi: 10.1126/science.283.5403.836 9933165

[69] Lei CL, Clerx M, Whittaker DG, Gavaghan DJ, de Boer TP, Mirams GR. Accounting for variability in ion current recordings using a mathematical model of artefacts in voltage-clamp experiments. Philos Trans R Soc A Math Phys Eng Sci. 2020;378(2173):20190348. doi: 10.1098/rsta.2019.0348 Epub 2020 May 25. ; 32448060 PMC7287334

[70] Lewis RS, Cahalan MD. Potassium and calcium channels in lymphocytes. Annu Rev Immunol. 1995;13: 623–653. doi: 10.1146/annurev.iy.13.040195.003203 7612237

[71] Schråter K-H, Ruppersberg JP, Wunder F, Rettig J, Stocker M, Pongs O. Cloning and functional expression of a TEA-sensitive A-type potassium channel from rat brain. FEBS Letters. 1991;278: 211–216. doi: 10.1016/0014-5793(91)80119-n 1840526

[72] Werry D, Eldstrom J, Wang Z, Fedida D. Single-channel basis for the slow activation of the repolarizing cardiac potassium current, IKs. PNAS. 2013;110: E996–E1005. doi: 10.1073/pnas.1214875110 23431135 PMC3600500

[73] Papreck JR, Martin EA, Lazzarini P, Kang D, Kim D. Modulation of K2P3.1 (TASK-1), K2P9.1 (TASK-3), and TASK-1/3 heteromer by reactive oxygen species. Pflugers Arch. 2012;464: 471–480. doi: 10.1007/s00424-012-1159-y 23007462 PMC3478902

[74] Zweifach A, Lewis RS. Mitogen-regulated Ca2+ current of T lymphocytes is activated by depletion of intracellular Ca2+ stores. Proc Natl Acad Sci U S A. 1993;90: 6295–6299. doi: 10.1073/pnas.90.13.6295 8392195 PMC46915

[75] Vazquez G, Wedel BJ, Aziz O, Trebak M, Putney JW. The mammalian TRPC cation channels. Biochim Biophys Acta. 2004;1742: 21–36. doi: 10.1016/j.bbamcr.2004.08.015 15590053

[76] Xu H, Ramsey IS, Kotecha SA, Moran MM, Chong JA, Lawson D, et al. TRPV3 is a calcium-permeable temperature-sensitive cation channel. Nature. 2002;418: 181. doi: 10.1038/nature00882 12077604

[77] Blair NT, Carvacho I, Chaudhuri D, Clapham DE, DeCaen P, Delling M, et al. Transient Receptor Potential channels (version 2019.4) in the IUPHAR/BPS Guide to Pharmacology Database. IUPHAR/BPS Guide to Pharmacology CITE. 2019;2019. doi: 10.2218/gtopdb/F78/2019.4

[78] Weinreich F, Jentsch TJ. Pores Formed by Single Subunits in Mixed Dimers of Different CLC Chloride Channels. J Biol Chem. 2001;276: 2347–2353. doi: 10.1074/jbc.M005733200 11035003

[79] Ridge FPG, Duszyk M, French AS. A large conductance, Ca2+-activated K+ channel in a human lung epithelial cell line (A549). Biochim Biophys Acta. 1997;1327: 249–258. doi: 10.1016/s0005-2736(97)00073-4 . 9271267

[80] Ouadid-Ahidouch H, Roudbaraki M, Delcourt P, Ahidouch A, Joury N, Prevarskaya N. Functional and molecular identification of intermediate-conductance Ca(2+)-activated K(+) channels in breast cancer cells: association with cell cycle progression. Am J Physiol Cell Physiol. 2004;287: C125–134. doi: 10.1152/ajpcell.00488.2003 14985237

[81] Girault A, Privé A, Trinh NTN, Bardou O, Ferraro P, Joubert P, et al. Identification of KvLQT1 K+ channels as new regulators of non-small cell lung cancer cell proliferation and migration. Int J Oncol. 2014;44: 838–848. doi: 10.3892/ijo.2013.2228 24366043

[82] Jang SH, Choi SY, Ryu PD, Lee SY. Anti-proliferative effect of Kv1.3 blockers in A549 human lung adenocarcinoma in vitro and in vivo. Eur J Pharmacol. 2011;651: 26–32. doi: 10.1016/j.ejphar.2010.10.066 21087602

[83] Li X, Zhang Q, Fan K, Li B, Li H, Qi H, et al. Overexpression of TRPV3 Correlates with Tumor Progression in Non-Small Cell Lung Cancer. Int J Mol Sci. 2016;17: 437. doi: 10.3390/ijms17040437 27023518 PMC4848893

[84] Yang L-L, Liu B-C, Lu X-Y, Yan Y, Zhai Y-J, Bao Q, et al. Inhibition of TRPC6 reduces non-small cell lung cancer cell proliferation and invasion. Oncotarget. 2017;8: 5123–5134. doi: 10.18632/oncotarget.14034 28030826 PMC5341750

[85] Bulk E, Ay A-S, Hammadi M, Ouadid-Ahidouch H, Schelhaas S, Hascher A, et al. Epigenetic dysregulation of KCa3.1 channels induces poor prognosis in lung cancer. Int J Cancer. 2015;137: 1306–1317. doi: 10.1002/ijc.29490 25704182

[86] Jiang H-N, Zeng B, Zhang Y, Daskoulidou N, Fan H, Qu J-M, et al. Involvement of TRPC channels in lung cancer cell differentiation and the correlation analysis in human non-small cell lung cancer. PLoS ONE. 2013;8: e67637. doi: 10.1371/journal.pone.0067637 23840757 PMC3695899

[87] Suo A, Childers A, D’Silva A, Petersen LF, Otsuka S, Dean M, et al. Cav3.1 overexpression is associated with negative characteristics and prognosis in non-small cell lung cancer. Oncotarget. 2018;9: 8573–8583. doi: 10.18632/oncotarget.24194 29492218 PMC5823575

[88] Sterratt D, Graham B, Gillies A, Willshaw D. Principles of Computational Modelling in Neuroscience. In: Principles of Computational Modelling in Neuroscience. Cambridge University Press, New York. Jun 2011. ISBN 978-0-521-87795-4

[89] Kowalewski JM, Uhlén P, Kitano H, Brismar H. Modeling the impact of store-operated Ca2+ entry on intracellular Ca2+ oscillations. Math Biosci. 2006;204: 232–249. doi: 10.1016/j.mbs.2006.03.001 16620876

[90] Marhl M, Gosak M, Perc M, Jane Dixon C, Green AK. Spatio-temporal modelling explains the effect of reduced plasma membrane Ca2+ efflux on intracellular Ca2+ oscillations in hepatocytes. J Theor Biol. 2008;252: 419–426. doi: 10.1016/j.jtbi.2007.11.006 18160078

[91] McIvor E, Coombes S, Thul R. Three-dimensional spatio-temporal modelling of store operated Ca2+ entry: Insights into ER refilling and the spatial signature of Ca2+ signals. Cell Calcium. 2018;73: 11–24. doi: 10.1016/j.ceca.2018.03.006 29880194

[92] Bose T, Cieślar-Pobuda A, Wiechec E. Role of ion channels in regulating Ca^2+^ homeostasis during the interplay between immune and cancer cells. Cell Death Dis. 2015;6: e1648. doi: 10.1038/cddis.2015.23 25695601 PMC4669790

[93] Numberger M, Draguhn A. Patch-Clamp-Technik. Spektrum, Akad. Verl.: Heidelberg, Berlin, Oxford, 1996.

