# Supplementary material for: A549 in-silico 1.0: A first computational model to simulate cell cycle dependent ion current modulation in the human lung adenocarcinoma
Source: PLoS Comput Biol. 2021 Jun 22;17(6):e1009091. doi: 10.1371/journal.pcbi.1009091 (PMC8219159; doi:10.1371/journal.pcbi.1009091)
Supplement: S1 Text — (DOCX) [file pcbi.1009091.s001.docx]

**Supporting Information**

A549 in-silico 1.0: A first computational model to simulate cell cycle dependent ion current modulation in the human lung adenocarcinoma

**S1 Text: Literature review of ion channels in the A549 cell line**

This review provides a summary of reported ion channels in the human lung cancer cell line A549 forming the basis for the A549 in-silico model. We discuss the role and function of the single ion channels and their impact on whole-cell electrophysiological and bioelectric properties, and their relevance on cell proliferation and tumor progression in the A549 cells.

**Potassium channels:**

Potassium conductance plays a distinct role in the cell cycle, governing for instance the membrane potential, affecting cell volume and changing the driving force for calcium, a crucial mediator of intracellular signals involved in the control of proliferation.[1,2] In particular, potassium ions are the main charge carrier in A549 cells and hence the cells exhibit a large number of various potassium channels.

One of these functionally expressed potassium channels is the voltage-gated, shaker-related **Kv1.3** channel. Inhibition of Kv1.3 by selective blocker Margatoxin (MgTX) and silencing of the channel by shRNA-Kv1.3 plasmids showed an anti-proliferative effect in A549 cells by impeding G1-S transition and entailing suppressed tumor growth in vivo.[3] In addition, whole-cell current recordings reveal about 60% reduction of the instant current density and depolarization of the membrane potential in presence of MgTX.[4]

Similarly, inhibition of **Kv1.1** channels with dendrotoxin-𝛋 induces reduction of proliferation by blocking G1-S transition in cell cycle progression, resulting in decreased tumor growth in nude mice.[5] Expression of Kv1.1 was localized in the nuclear fraction of A549 cells by Western blot analysis, but was not reported in the cell membrane.[6] RT-PCR analysis further confirmed a stable expression of **Kv1.2, Kv2.1** and **Kv2.2** in A549 cells, whereas Kv1.2 is only localized in the membrane and Kv2.2 is found in the nuclear and cytosolic fraction.[5,6] However, functional expression of the channels in the cell membrane of A549 cells has not yet been verified.

In regard to the Kv3 subfamily, protein expression of **Kv3.1**, a delayed rectifier voltage-gated potassium channel, and two a-type potassium channels **Kv3.3** and **Kv3.4** could be verified by Western blot analysis.[7] These channels are known to be oxygen sensitive and act as an oxidation sensor, whereby hypoxia, in general associated with metastasis and proliferation, leads to a reversible block and membrane depolarization.[7,8] Blocking of Kv3.1 and Kv3.4 with BDS-II, a Kv3 subfamily-specific blocker, showed inhibition of cell migration and invasion of A549 cells, which is essential for metastasis.[7]

Functional expression of **Kv7.1** (KvLQT1) in A549 cells was examined in a survey of Girault et al. [9] and correlates with wound healing, motility and progression. Blocking of these channels with clofilium and chromanol reduced wound healing rates up to 31%, depending on the blocker concentration, indicating a decrease of cell motility. In addition, inhibition of Kv7.1 significantly interferes with cell cycle progression by decreasing the proportion of cells in the S and G2/M phases, resulting in a reduction of cell growth.[9] Related analysis shows elevated KvLQT1 levels in 65% of human adenocarcinoma samples, suggesting a therapeutic potential of KvLQT1 blockade in vivo.[9]

**Kv9.3** (KCNS3 [10]) is part of the electrically silent potassium channel subfamily that does not exhibit a potassium current of its own, but modulates in combination the electrophysiological and pharmacological properties of Kv2.1 and Kv2.2, respectively. These channels are substantial for excitable cells, whereby Kv9.3 is also largely expressed in non-excitable lung tissue.[11,12] Lee et al. [11] investigated the role of Kv9.3 channel itself on tumor progression in HCT15 (colon carcinoma) and A549 cells. The knockdown of Kv9.3 by siRNA decreased viability of the A549 cells by 29% due to an increase of cells in the G0/G1-phase and corresponding decrease of S phase cells. This was caused by changes in expression of regulatory proteins related to G1-S transition, resulting in a reduction of proliferation. Moreover, the knockdown of Kv9.3 reduced tumor growth in in vivo experiments (statistical significance noted on the 5th week), affirming the involvement of Kv9.3 in A549 cell proliferation.[11]

**Kv10.1** (EAG1) and **Kv11.1** (hERG1) channels are overexpressed in a number of different tumor types and clearly implicated in cell proliferation and tumor progression.[13–17] In A549 cells, mRNA expression of Kv10.1 and Kv11.1 was reported by Girault et al. [9] and Chen et al. [18]. Compared to other cancer cell lines (e.g. HT-29, MCF-7), A549 cells exhibit only a low Kv11.1 expression. Treatment with erythromycin, a hERG1 blocker, therefore showed less sensitivity and effect on cell proliferation compared to cell lines highly expressing Kv11.1.[18] In addition, neither application of astemizole (EAG and ERG inhibitor) nor ergtoxin (a specific ERG inhibitor) lead to significant changes in wound-healing rates.[9] However, compared to other cancer cell types, the specific role of EAG and hERG is not entirely declared in A549 cells.

In addition to solely voltage-gated potassium channels, ligand-gated potassium channels are also present in A549 cells. The KCNN4 gene encodes for the voltage-independent intermediate-conductance Ca-activated K^+^ channel **KCa3.1** (hIK, SK4), which is known to operate in conjunction with Kv1.3 [13]. KCa3.1 is upregulated in several tumors, such as prostate and breast cancer, correlating with tumor grade, metastasis state, migration as well as cell cycle progression, proliferation and apoptosis.[19–21] Regarding NSCLC, high expression levels of KCa3.1 are related to aggressive behavior and correlate with poor prognosis.[21]. Similarly, A549 cells show strong expression of hIK channels, as determined by Roth et al. [22].

hIK channels are not by default active. The activity is strongly related to the cell cycle and differs significantly during the phases, with which the block of current can vary between 28 to 95 %.[22] Blocking of hIK channels with TRAM-24 in A549 cells resulted in decreased proliferation, migration and also tumor growth in vivo, emphasizing the important role of KCa3.1 activity in cell cycle progression.[21,22] Inhibition of the channels generally depolarizes the membrane potential, whereas activation leads to a hyperpolarization, determined to promote G1/S transition in MCF-7 cells.[23] Hence, inhibition of hIK channels in A549 is in agreement with experimental observations which increases the number of cells in the G0/G1 phase and prevent cell cycle progression.[22]

In addition, A549 show functional expression of **KCa1.1** (BK), ascertained by Ridge et al. [24] and Jovanvovic´ et al. [25]. In comparison to solely calcium gated hIKs [26], BKs show additional voltage-sensitivity and become activated at membrane depolarization.[24]

G-protein-gated inwardly rectifying potassium channels **(GIRK1-4)** are important for maintenance of the resting membrane potential and therefor essential for proliferation.[27,28] GIRK1 expression is reported in various cancer types [29–31] and positively correlated with tumor progression in non-small cell lung tumors [32] and breast cancer [33–36]. Plummer et al. [37] identified mRNA expression of GIRK 1-4 in A549 cells, however, at least no protein expression of GIRK1 channels could be detected [37]. Hence, further research is needed to prove the functional expression of GIRK channels in A549 cells.

A survey of Leithner et al. [38] regarding non-small cell lung cancer cells confirms a strong functional expression of **TASK-1** (K2P3.1) and low expression levels of **TASK-3** (K2P9.1, KCNK9) channels in A549 cells. These two-pore domain potassium channels depict a non-inactivating background potassium current, important for K^+^ efflux and subsequently, in combination with Na^+^/K^+^-ATPase, for controlling the resting membrane potential.[38,39] Overexpression of TASK-3 has been observed in breast, lung, colon and prostate cancer, influencing proliferation and apoptosis as well as migration and invasion.[40–43] Similarly, functional expression of TASK-1 was reported in various cancer types (e.g. medulloblastoma [44], neuroblastoma [45], osteosarcoma [46] and in breast cancer cell line MCF-7 [43]). Investigation of TASK-1 protein expression in human NSCLC and normal lung tissue samples revealed variable, but comparable levels between samples of NSCLC and the corresponding normal lung tissue.[38] In A549 cells, inhibition of TASK-1 current reduced the non-inactivating current and led to significant depolarization of the membrane potential accompanied with reduced proliferation, mitosis rates and enhanced apoptosis.[38]

**Sodium channels:**

Sodium current carried by voltage-gated sodium channels (VGSCs, Nav) is strongly associated with metastatic behavior in various cancer types (for review see [47]). In comparison to strongly invasive NSCLC lung cancer cell lines, non-invasive A549 cells possess Nav mRNA expression, albeit do not exhibit functional channels and lack of Nav current.[48,49]

Two voltage-insensitive channel types are indeed reported in A549, including inwardly-rectifying amiloride-sensitive epithelial sodium channels (**ENaCs**), investigated by Lazrak et al. [50], and acid-sensing (proton-gated) ion channels (**ASICs**), which are highly active at low pH-levels [51]. ENaCs and ASICs are suggested to be involved in cell migration and proliferation of different cancer types such as melanoma, colon, glioma and breast cancer cells.[52–54] The ASIC-like current in A549 cells, as ascertained by Wu et al. [51], is mainly driven by ASIC1 channels, whereas ASIC2 and ASIC3 are also functionally expressed. Subsequent experiments with transfected A549 cells overexpressing ASIC1a demonstrated enhanced proliferation and migration induced by extracellular acidosis, indicating the involvement of ASICs in those processes. Since ASIC1 channels are also permeable to calcium [55], extracellular acidosis led to an increase in intracellular calcium concentration, which is probably responsible for the effects on proliferation and migration.[51]

**Calcium channels:**

Calcium signalling is in general of particular importance in numerous processes such as proliferation, differentiation, motility, apoptosis, secretion or activation of various enzymes.[56–58] Calcium entry in non-excitable cells occurs via voltage-gated (Cav, VGCCs) but mainly by non-voltage activated channels, including store-operated Ca^2+^ channels (SOC) and transient receptor potential channels (TRP).[56] Analysis of gene expression levels revealed overexpression of VGCCs in different tumor cells, reviewed by Buchanan et al. [57], Phan et al. [58] and Wang et al. [59]. The expression levels of several Cav channels differ within different forms of lung cancer. For instance, Cav1.3 appears to be downregulated in lung adenocarcinoma and squamous cell lung carcinoma, but upregulated in lung carcinoid tumors compared to normal lung tissue.[58]

The gene *CACNA1G* encodes for the T-type calcium channel **Cav3.1**, which overexpression is generally associated with negative characteristics and prognosis in NSCLCs.[60] Cav3.1 expression was reported in the nucleus of certain cell types [61]. In A549 cells, anti-proliferative effects and apoptotic activities of putative antagonists (e.g. BK10040, KYS05090) were described by Choi et al. [62] and Rim et al. [63], suggesting the functional expression of Cav3.1 channels. In addition, as ascertained by Rim et al. [64], T-type channel blockade with KYS05047 induces cell cycle arrest in G1 phase and leads to an inhibition of tumor growth in vivo by decreasing intracellular calcium levels. Thus, KYS05090 and KYS05047 might serve as potential in vivo targets to limit further tumor progression.[63] In addition to localization in the cell membrane, Cav3.1 expression was also reported in the nucleus membrane of certain cell types [61]. Patch clamp experiments in A549 revealed characteristic currents only in two out of 25 cells [60], probably indicating a cell cycle dependent expression or predominant expression in the nucleus and only weak Cav3.1 expression in the plasma membrane of A549 cells.

**CRAC** (calcium-release activated calcium) channels are non-exclusive, but highly calcium selective SOCs, that enable calcium influx into the cell by depletion of the Ca-stores of the endoplasmatic reticulum.[65] STIM1 (stromal interaction molecule) probably acts as a calcium sensor [66] which activates the channel by interaction with the pore-forming subunit ORAI1/CRACM1. Knockdown of STIM1 and ORAI1/CRACM1 can inhibit proliferation of endothelial cells, as a result of reduced calcium influx [67]. A study of Hou et al. [68] confirms expression of STIM1 and ORAI1/CRACM1 on RNA and protein level in A549 cells. Transfected A549 cells, overexpressing ORAI1/CRACM1 component of CRAC, attenuates Ca influx resulting in inhibition of proliferation due to G0/G1 cell cycle arrest.[68]

**Chloride channels:**

Chloride channels regulated by voltage (ClC), cyclic adenosine monophosphate (CFTR) and calcium are important players in transepithelial secretion, pH and cell volume regulation, proliferation as well as stabilization of the membrane potential.[13] ClCs are involved in cell cycle progression and are responsible for depolarization, which is necessary for M phase initiation and cell shrinkage prior to division. The inhibition of ClC can thus lengthen the cell cycle and provoke cell cycle arrest in G2-phase.[13] In particular overexpression of CLC-3 has been proved to play a crucial role in cell migration and invasion, hence, increasing aggressiveness and metastasis of certain malignant tumours including for example cervical carcinoma, breast cancer and glioma.[13,20,69] Compared to normal lung tissue, CLC-3 and CLC-7 gene expression has shown to be notably upregulated in lung adenocarcinoma tissue.[70] By contrast, A549 cells barely express CLC-3 [71] and characteristic chloride current is assumed to be mainly driven by CFTR (cystic fibrosis transmembrane conductance regulator) and the voltage-gated chloride channel CLC-2.[72,73]

**Non-selective ion channels:**

TRP channels, activated by diverse factors ranging from temperature to changes in pH-value, are non-selective cation channels, similarly important for calcium influx in non-excitable cells.[56] In lung cancer, channels out of the TRPC, TRPV and TRPM subfamilies are expressed [56], whereby expression of TRPC 1, 3, and 6 correlate with differentiation grades in NSCLC.[74] Concerning A549 cells, Jiang et al. [74] reported decreased cell mitosis by inhibition of TRPC (**TRPC1 and TRPC3/6**). Moreover, blocking of **TRPC 1, 4 and 6** significantly inhibited proliferation, whereas overexpression of **TRPC1 and 6** increased proliferation.[74] Tajeddine and Gailly [75] confirmed the involvement of **TRPC1** in cell proliferation, where knockdown induced cell cycle arrest in the G0/G1 phase and correspondingly led to inhibition of proliferation. A recent investigation of Yang et al. [76] revealed high expression levels of **TRPC6** during S-G2/M transition, compared to cells in the G1 phase. In addition, inhibition of TRPC6 results in reduced invasion and cell proliferation by cell cycle arrest at the S-G2/M phase.[76]

Research regarding TRPV channels determined overexpression of **TRPV3** (transient receptor potential vanilloid 3) channels in NSCLC. Inhibition of TRPV3 decreases intracellular calcium and seems to induce cell cycle arrest at G1-S transition, resulting in a reduced proliferation capacity and suppressed lung cancer cell growth, indicating a functional expression of TRPV3 channels in A549 cells. [77]

**Supporting References**

1. Ouadid-Ahidouch H, Ahidouch A. K+ channels and cell cycle progression in tumor cells. Front Physiol. 2013;4. doi:10.3389/fphys.2013.00220

2. Urrego D, Tomczak AP, Zahed F, Stühmer W, Pardo LA. Potassium channels in cell cycle and cell proliferation. Philos Trans R Soc Lond B Biol Sci. 2014;369. doi:10.1098/rstb.2013.0094

3. Jang SH, Choi SY, Ryu PD, Lee SY. Anti-proliferative effect of Kv1.3 blockers in A549 human lung adenocarcinoma in vitro and in vivo. Eur J Pharmacol. 2011;651: 26–32. doi:10.1016/j.ejphar.2010.10.066

4. Roth B. Exposure to sparsely and densely ionizing irradiation results in an immediate activation of K+ channels in A549 cells and in human peripheral blood lymphocytes. PhD thesis. Technische Universität Darmstadt. 2014.

5. Jang SH, Ryu PD, Lee SY. Dendrotoxin-κ suppresses tumor growth induced by human lung adenocarcinoma A549 cells in nude mice. J Vet Sci. 2011;12: 35–40. doi:10.4142/jvs.2011.12.1.35

6. Jang SH, Byun JK, Jeon W-I, Choi SY, Park J, Lee BH, et al. Nuclear Localization and Functional Characteristics of Voltage-gated Potassium Channel Kv1.3. J Biol Chem. 2015;290: 12547–12557. doi:10.1074/jbc.M114.561324

7. Song MS, Park SM, Park JS, Byun JH, Jin HJ, Seo SH, et al. Kv3.1 and Kv3.4, Are Involved in Cancer Cell Migration and Invasion. Int J Mol Sci. 2018;19. doi:10.3390/ijms19041061

8. Patel AJ, Honoré E. Molecular physiology of oxygen-sensitive potassium channels. Eur Respir J. 2001;18: 221–227.

9. Girault A, Privé A, Trinh NTN, Bardou O, Ferraro P, Joubert P, et al. Identification of KvLQT1 K+ channels as new regulators of non-small cell lung cancer cell proliferation and migration. Int J Oncol. 2014;44: 838–848. doi:10.3892/ijo.2013.2228

10. Gutman GA, Chandy KG, Grissmer S, Lazdunski M, Mckinnon D, Pardo LA, et al. International Union of Pharmacology. LIII. Nomenclature and Molecular Relationships of Voltage-Gated Potassium Channels. Pharmacol Rev. 2005;57: 473–508. doi:10.1124/pr.57.4.10

11. Lee J-H, Park J-W, Byun JK, Kim HK, Ryu PD, Lee SY, et al. Silencing of voltage-gated potassium channel KV9.3 inhibits proliferation in human colon and lung carcinoma cells. Oncotarget. 2015;6: 8132–8143.

12. Patel AJ, Lazdunski M, Honoré E. Kv2.1/Kv9.3, a novel ATP-dependent delayed-rectifier K+ channel in oxygen-sensitive pulmonary artery myocytes. EMBO J. 1997;16: 6615–6625. doi:10.1093/emboj/16.22.6615

13. Rao VR, Perez-Neut M, Kaja S, Gentile S. Voltage-Gated Ion Channels in Cancer Cell Proliferation. Cancers (Basel). 2015;7: 849–875. doi:10.3390/cancers7020813

14. Pardo LA, Contreras-Jurado C, Zientkowska M, Alves F, Stühmer W. Role of voltage-gated potassium channels in cancer. J Membr Biol. 2005;205: 115–124. doi:10.1007/s00232-005-0776-1

15. Pardo LA, del Camino D, Sánchez A, Alves F, Brüggemann A, Beckh S, et al. Oncogenic potential of EAG K(+) channels. EMBO J. 1999;18: 5540–5547. doi:10.1093/emboj/18.20.5540

16. Arcangeli A. Expression and role of hERG channels in cancer cells. Novartis Found Symp. 2005;266: 225–232; discussion 232-234.

17. Asher V, Sowter H, Shaw R, Bali A, Khan R. Eag and HERG potassium channels as novel therapeutic targets in cancer. World J Surg Oncol. 2010;8: 113. doi:10.1186/1477-7819-8-113

18. Chen S-Z, Jiang M, Zhen Y. HERG K+ channel expression-related chemosensitivity in cancer cells and its modulation by erythromycin. Cancer Chemother Pharmacol. 2005;56: 212–220. doi:10.1007/s00280-004-0960-5

19. Yang M, Brackenbury WJ. Membrane potential and cancer progression. Front Physiol. 2013;4. doi:10.3389/fphys.2013.00185

20. Lastraioli E, Iorio J, Arcangeli A. Ion channel expression as promising cancer biomarker. Biochim Biophys Acta. 2015;1848: 2685–2702. doi:10.1016/j.bbamem.2014.12.016

21. Bulk E, Ay A-S, Hammadi M, Ouadid‐Ahidouch H, Schelhaas S, Hascher A, et al. Epigenetic dysregulation of KCa3.1 channels induces poor prognosis in lung cancer. Int J Cancer. 2015;137: 1306–1317. doi:10.1002/ijc.29490

22. Roth B, Gibhardt CS, Becker P, Gebhardt M, Knoop J, Fournier C, et al. Low-dose photon irradiation alters cell differentiation via activation of hIK channels. Pflugers Arch. 2015;467: 1835–1849. doi:10.1007/s00424-014-1601-4

23. Ouadid-Ahidouch H, Roudbaraki M, Delcourt P, Ahidouch A, Joury N, Prevarskaya N. Functional and molecular identification of intermediate-conductance Ca(2+)-activated K(+) channels in breast cancer cells: association with cell cycle progression. Am J Physiol Cell Physiol. 2004;287: C125-134. doi:10.1152/ajpcell.00488.2003

24. Ridge FPG, Duszyk M, French AS. A large conductance, Ca2+-activated K+ channel in a human lung epithelial cell line (A549). Biochim Biophys Acta. 1997;1327: 249–258. doi:10.1016/S0005-2736(97)00073-4

25. Jovanović S, Crawford RM, Ranki HJ, Jovanović A. Large Conductance Ca2+-Activated K+ Channels Sense Acute Changes in Oxygen Tension in Alveolar Epithelial Cells. Am J Respir Cell Mol Biol. 2003;28: 363–372.

26. Sforna L, Megaro A, Pessia M, Franciolini F, Catacuzzeno L. Structure, Gating and Basic Functions of the Ca2+-activated K Channel of Intermediate Conductance. Curr Neuropharmacol. 2018;16: 608–617. doi:10.2174/1570159X15666170830122402

27. Brackenbury WJ. Chapter 6 - Ion Channels in Cancer. In: Ion Channels in Health and Disease. Academic Press; 2016. pp. 131–163. doi:10.1016/B978-0-12-802002-9.00006-6

28. Slesinger PA (Ed.), Wickman K (Ed.). Structure to Function of G Protein-Gated Inwardly Rectifying (GIRK) Channels. Vol 123, 1^st^ edition. Academic Press; 2015. eBook ISBN: 9780128026311

29. Brevet M, Ahidouch A, Sevestre H, Merviel P, El Hiani Y, Robbe M, et al. Expression of K+ channels in normal and cancerous human breast. Histol Histopathol. 2008;23: 965–972. doi:10.14670/HH-23.965

30. Brevet M, Fucks D, Chatelain D, Regimbeau J-M, Delcenserie R, Sevestre H, et al. Deregulation of 2 Potassium Channels in Pancreas Adenocarcinomas. Pancreas. 2009;38: 649–654. doi:10.1097/MPA.0b013e3181a56ebf

31. Stringer BK, Cooper AG, Shepard SB. Overexpression of the G-protein inwardly rectifying potassium channel 1 (GIRK1) in primary breast carcinomas correlates with axillary lymph node metastasis. Cancer Res. 2001;61: 582–588.

32. Takanami I, Inoue Y, Gika M. G-protein inwardly rectifying potassium channel 1 (GIRK 1) gene expression correlates with tumor progression in non-small cell lung cancer. BMC Cancer. 2004;4: 79. doi:10.1186/1471-2407-4-79

33. Kammerer S, Sokolowski A, Hackl H, Jahn S, Asslaber M, Symmans F, et al. Overexpression of G protein-activated inward rectifier potassium channel 1 (GIRK1) is associated with lymph node metastasis and poor prognosis in breast cancer. Ann Oncol. 2014;25: i13–i14. doi:10.1093/annonc/mdu066.21

34. Rezania S, Kammerer S, Li C, Steinecker-Frohnwieser B, Gorischek A, DeVaney TTJ, et al. Overexpression of KCNJ3 gene splice variants affects vital parameters of the malignant breast cancer cell line MCF-7 in an opposing manner. BMC Cancer. 2016;16: 628. doi:10.1186/s12885-016-2664-8

35. Kammerer S, Sokolowski A, Hackl H, Platzer D, Jahn SW, El-Heliebi A, et al. KCNJ3 is a new independent prognostic marker for estrogen receptor positive breast cancer patients. Oncotarget. 2016;7: 84705–84717. doi:10.18632/oncotarget.13224

36. Schratter G, Scheruebel S, Langthaler S, Ester K, Pelzmann B, Ghaffari-Tabrizi-Wizsy N, et al. GIRK1 triggers multiple cancer-related pathways in the benign mammary epithelial cell line MCF10A. Sci Rep. 2019;9: 1–18. doi:10.1038/s41598-019-55683-w

37. Plummer HK, Dhar MS, Cekanova M, Schuller HM. Expression of G-protein inwardly rectifying potassium channels (GIRKs) in lung cancer cell lines. BMC Cancer. 2005;5: 104. doi:10.1186/1471-2407-5-104

38. Leithner K, Hirschmugl B, Li Y, Tang B, Papp R, Nagaraj C, et al. TASK-1 Regulates Apoptosis and Proliferation in a Subset of Non-Small Cell Lung Cancers. PLoS One. 2016;11. doi:10.1371/journal.pone.0157453

39. Lesage F, Lazdunski M. Molecular and functional properties of two-pore-domain potassium channels. Am J Physiol Renal Physiol. 2000;279: F793-801. doi:10.1152/ajprenal.2000.279.5.F793

40. Mu D, Chen L, Zhang X, See L-H, Koch CM, Yen C, et al. Genomic amplification and oncogenic properties of the KCNK9 potassium channel gene. Cancer Cell. 2003;3: 297–302.

41. Innamaa A, Jackson L, Asher V, Van Shalkwyk G, Warren A, Hay D, et al. Expression and prognostic significance of the oncogenic K2P potassium channel KCNK9 (TASK-3) in ovarian carcinoma. Anticancer Res. 2013;33: 1401–1408.

42. Meuth SG, Herrmann AM, Ip CW, Kanyshkova T, Bittner S, Weishaupt A, et al. The two-pore domain potassium channel TASK3 functionally impacts glioma cell death. J Neurooncol. 2008;87: 263–270. doi:10.1007/s11060-008-9517-5

43. Lee G-W, Park HS, Kim E-J, Cho Y-W, Kim G-T, Mun Y-J, et al. Reduction of breast cancer cell migration via up-regulation of TASK-3 two-pore domain K+ channel. Acta Physiol (Oxf). 2012;204: 513–524. doi:10.1111/j.1748-1716.2011.02359.x

44. Ernest NJ, Logsdon NJ, McFerrin MB, Sontheimer H, Spiller SE. Biophysical properties of human medulloblastoma cells. J Membr Biol. 2010;237: 59–69. doi:10.1007/s00232-010-9306-x

45. Hao X, Li X. The knockdown of TASK-1 channels improved the proliferation of N2A cells. J Mol Neurosci. 2015;55: 314–317. doi:10.1007/s12031-014-0323-6

46. Li X, Dong X, Zheng S, Xiao J. Expression and localization of TASK-1, -2 and -3 channels in MG63 human osteoblast-like cells. Oncol Lett. 2013;5: 865–869. doi:10.3892/ol.2012.1088

47. Brackenbury WJ. Voltage-gated sodium channels and metastatic disease. Channels (Austin). 2012;6: 352–361. doi:10.4161/chan.21910

48. Roger S, Rollin J, Barascu A, Besson P, Raynal P-I, Iochmann S, et al. Voltage-gated sodium channels potentiate the invasive capacities of human non-small-cell lung cancer cell lines. Int J Biochem Cell Biol. 2007;39: 774–786. doi:10.1016/j.biocel.2006.12.007

49. Campbell TM, Main MJ, Fitzgerald EM. Functional expression of the voltage-gated Na^+^-channel Nav1.7 is necessary for EGF-mediated invasion in human non-small cell lung cancer cells. J Cell Sci. 2013;126: 4939–4949. doi:10.1242/jcs.130013

50. Lazrak A, Samanta A, Matalon S. Biophysical properties and molecular characterization of amiloride-sensitive sodium channels in A549 cells. Am J Physiol Lung Cell Mol Physiol. 2000;278: L848-857. doi:10.1152/ajplung.2000.278.4.L848

51. Wu Y, Gao B, Xiong Q-J, Wang Y-C, Huang D-K, Wu W-N. Acid-sensing ion channels contribute to the effect of extracellular acidosis on proliferation and migration of A549 cells. Tumour Biol. 2017;39: 1010428317705750. doi:10.1177/1010428317705750

52. Qadri YJ, Rooj AK, Fuller CM. ENaCs and ASICs as therapeutic targets. Am J Physiol Cell Physiol. 2012;302: C943-965. doi:10.1152/ajpcell.00019.2012

53. Berdiev BK, Xia J, McLean LA, Markert JM, Gillespie GY, Mapstone TB, et al. Acid-sensing Ion Channels in Malignant Gliomas. J Biol Chem. 2003;278: 15023–15034. doi:10.1074/jbc.M300991200

54. Gupta SC, Singh R, Asters M, Liu J, Zhang X, Pabbidi MR, et al. Regulation of breast tumorigenesis through acid sensors. Oncogene. 2016;35: 4102–4111. doi:10.1038/onc.2015.477

55. Gründer S, Pusch M. Biophysical properties of acid-sensing ion channels (ASICs). Neuropharmacology. 2015;94: 9–18. doi:10.1016/j.neuropharm.2014.12.016

56. Déliot N, Constantin B. Plasma membrane calcium channels in cancer: Alterations and consequences for cell proliferation and migration. Biochim Biophys Acta. 2015;1848: 2512–2522. doi:10.1016/j.bbamem.2015.06.009

57. Buchanan PJ, McCloskey KD. CaV channels and cancer: canonical functions indicate benefits of repurposed drugs as cancer therapeutics. Eur Biophys J. 2016;45: 621–633. doi:10.1007/s00249-016-1144-z

58. Phan NN, Wang C-Y, Chen C-F, Sun Z, Lai M-D, Lin Y-C. Voltage-gated calcium channels: Novel targets for cancer therapy. Oncol Lett. 2017;14: 2059–2074. doi:10.3892/ol.2017.6457

59. Wang C-Y, Lai M-D, Phan NN, Sun Z, Lin Y-C. Meta-Analysis of Public Microarray Datasets Reveals Voltage-Gated Calcium Gene Signatures in Clinical Cancer Patients. PLoS ONE. 2015;10: e0125766. doi:10.1371/journal.pone.0125766

60. Suo A, Childers A, D’Silva A, Petersen LF, Otsuka S, Dean M, et al. Cav3.1 overexpression is associated with negative characteristics and prognosis in non-small cell lung cancer. Oncotarget. 2018;9: 8573–8583. doi:10.18632/oncotarget.24194

61. McKay BE, McRory JE, Molineux ML, Hamid J, Snutch TP, Zamponi GW, et al. Ca(V)3 T-type calcium channel isoforms differentially distribute to somatic and dendritic compartments in rat central neurons. Eur J Neurosci. 2006;24: 2581–2594. doi:10.1111/j.1460-9568.2006.05136.x

62. Choi DL, Jang SJ, Cho S, Choi H-E, Rim H-K, Lee K-T, et al. Inhibition of cellular proliferation and induction of apoptosis in human lung adenocarcinoma A549 cells by T-type calcium channel antagonist. Bioorg Med Chem Lett. 2014;24: 1565–1570. doi:10.1016/j.bmcl.2014.01.071

63. Rim H-K, Cho S, Shin D-H, Chung K-S, Cho Y-W, Choi J-H, et al. T-type Ca2+ channel blocker, KYS05090 induces autophagy and apoptosis in A549 cells through inhibiting glucose uptake. Molecules. 2014;19: 9864–9875. doi:10.3390/molecules19079864

64. Rim H-K, Lee H-W, Choi IS, Park JY, Choi HW, Choi J-H, et al. T-type Ca2+ channel blocker, KYS05047 induces G1 phase cell cycle arrest by decreasing intracellular Ca2+ levels in human lung adenocarcinoma A549 cells. Bioorg Med Chem Lett. 2012;22: 7123–7126. doi:10.1016/j.bmcl.2012.09.076

65. Prakriya M. The molecular physiology of CRAC channels. Immunol Rev. 2009;231: 88–98. doi:10.1111/j.1600-065X.2009.00820.x

66. Roos J, DiGregorio PJ, Yeromin AV, Ohlsen K, Lioudyno M, Zhang S, et al. STIM1, an essential and conserved component of store-operated Ca2+ channel function. J Cell Biol. 2005;169: 435–445. doi:10.1083/jcb.200502019

67. Abdullaev Iskandar F., Bisaillon Jonathan M., Potier Marie, Gonzalez Jose C., Motiani Rajender K., Trebak Mohamed. Stim1 and Orai1 Mediate CRAC Currents and Store-Operated Calcium Entry Important for Endothelial Cell Proliferation. Circ Res. 2008;103: 1289–1299. doi:10.1161/01.RES.0000338496.95579.56

68. Hou M-F, Kuo H-C, Li J-H, Wang Y-S, Chang C-C, Chen K-C, et al. Orai1/CRACM1 overexpression suppresses cell proliferation via attenuation of the store-operated calcium influx-mediated signalling pathway in A549 lung cancer cells. Biochim Biophys Acta. 2011;1810: 1278–1284. doi:10.1016/j.bbagen.2011.07.001

69. Guan Y, Xie Y, Zhou H, Shi H, Zhu Y, Zhang X, et al. Overexpression of chloride channel-3 (ClC-3) is associated with human cervical carcinoma development and prognosis. Cancer Cell Int. 2019;19. doi:10.1186/s12935-018-0721-x

70. Ko J-H, Gu W, Lim I, Bang H, Ko EA, Zhou T. Ion Channel Gene Expression in Lung Adenocarcinoma: Potential Role in Prognosis and Diagnosis. PLoS One. 2014;9. doi:10.1371/journal.pone.0086569

71. Qin C, He B, Dai W, Lin Z, Zhang H, Wang X, et al. The impact of a chlorotoxin-modified liposome system on receptor MMP-2 and the receptor-associated protein ClC-3. Biomaterials. 2014;35: 5908–5920. doi:10.1016/j.biomaterials.2014.03.077

72. Canella R, Martini M, Borriello R, Cavicchio C, Muresan XM, Benedusi M, et al. Modulation of Chloride Currents in Human Lung Epithelial Cells Exposed to Exogenous Oxidative Stress. J Cell Physiol. 2017;232: 1817–1825. doi:10.1002/jcp.25705

73. Cuppoletti J, Tewari KP, Sherry AM, Kupert EY, Malinowska DH. ClC-2 Cl− channels in human lung epithelia: activation by arachidonic acid, amidation, and acid-activated omeprazole. Am J Physiol Cell Physiol. 2001;281: C46–C54. doi:10.1152/ajpcell.2001.281.1.C46

74. Jiang H-N, Zeng B, Zhang Y, Daskoulidou N, Fan H, Qu J-M, et al. Involvement of TRPC channels in lung cancer cell differentiation and the correlation analysis in human non-small cell lung cancer. PLoS ONE. 2013;8: e67637. doi:10.1371/journal.pone.0067637

75. Tajeddine N, Gailly P. TRPC1 Protein Channel Is Major Regulator of Epidermal Growth Factor Receptor Signaling. J Biol Chem. 2012;287: 16146–16157. doi:10.1074/jbc.M112.340034

76. Yang L-L, Liu B-C, Lu X-Y, Yan Y, Zhai Y-J, Bao Q, et al. Inhibition of TRPC6 reduces non-small cell lung cancer cell proliferation and invasion. Oncotarget. 2017;8: 5123–5134. doi:10.18632/oncotarget.14034

77. Li X, Zhang Q, Fan K, Li B, Li H, Qi H, et al. Overexpression of TRPV3 Correlates with Tumor Progression in Non-Small Cell Lung Cancer. Int J Mol Sci. 2016;17: 437. doi:10.3390/ijms17040437
