# Supplementary material for: A549 in-silico 1.0: A first computational model to simulate cell cycle dependent ion current modulation in the human lung adenocarcinoma
Source: PLoS Comput Biol. 2021 Jun 22;17(6):e1009091. doi: 10.1371/journal.pcbi.1009091 (PMC8219159; doi:10.1371/journal.pcbi.1009091)
Supplement: S2 Text — (DOCX) [file pcbi.1009091.s002.docx]

**Supporting Information**

A549 in-silico 1.0: A first computational model to simulate cell cycle dependent ion current modulation in the human lung adenocarcinoma

**S2 Text: Source code A549 in-silico model**

Overview

The A549 matlab model consists of 3 program parts for data visualization, optimization of ion channel numbers and model evaluation. All functions can be executed via the **A549 in-silico main file (*A549 in-silico main.m*)**:

- Load patch-clamp data: read in and visualize experimental data
- Optimization: optimization of ion channel numbers for cells in G0 and G1 phase
- Model evaluation: simulation of whole-cell current for different voltage-clamp protocols and calculation of the root mean square error values (RMS) between simulated and measured data

Functions:

**Load patch-clamp data *(load_patch_clamp_data.m):*** loads the pre-processed patch-clamp data (patch_clamp_data_activation.mat, patch_clamp_data_deactivation.mat, patch_clamp_data_ramp.mat) and allows visualization of the averaged whole-cell currents of each experiment and cell group. mG0_1.mat to mG0_11.mat denote the whole-cell current patch-clamp recordings of cells in G0 phase (n=11). mG0_1.mat to mG0_5.mat corresponds to the whole-cell current patch-clamp recordings of cells in G1 phase (n=5).

**Optimization (*optimization.m*):** defines the model parameters and the pulse protocol for simulating the individual hidden Markov models and the constraints for optimization. Hidden Markov models are simulated for the specific test voltages and the expected open probabilities of the ion channels are calculated. Based on the expected open probabilities individual ion channel numbers (NOCs_global) are estimated by particle swarm optimization (PSO).

**Model evaluation *(model_evaluation.m):*** Simulates hidden Markov models of all ion channels for different measurement protocols. Allows hypothesis testing, visualization of the simulated whole-cell current curves and construction of the corresponding current-voltage curves for determination of the reversal potentials.

***Hidden Markov Models (HMM):***

Kv1.3 Markov model: *Kv_1_3.m*

Kv3.1 Markov model: *Kv_3_1.m*

Kv3.4 Markov model: *Kv_3_4.m*

Kv7.1 Markov model: *Kv_7_1.m*

KCa1.1 Markov model: *KCa_1_1.m*

KCa3.1 Markov model: *KCa_3_1.m*

TASK-1 Markov model: *TASK1.m*

TRPC6 Model: *TRPC6.m*

TRPV3 Model: *TRPV3.m*

CLC-2 Markov model: *CLC_2.m*

CRAC Model: *CRAC1.m*, *P_CRAC1.m*

***Simulate pulse protocol:***

*simulate_pulse_protocol.m:* simulates pulse protocols for ion channels Kv1.3, Kv3.1, Kv3.4, Kv7.1, KCa1.1, KCa3.1, TASK-1 and CLC-2.

*simulate_pulse_protocol_TRPC.m:* simulates pulse protocol of TRPC6 channels.

*simulate_pulse_protocol_TRPV.m:* simulates pulse protocol of TRPV3 channels.

Source Code:

The source code of the A549 in-silico model 1.0 is available for download via the link: <https://www.tugraz.at/en/institutes/hce/research-working-groups/research-data/a549-in-silico/>
