# Supplementary material for: A549 in-silico 1.0: A first computational model to simulate cell cycle dependent ion current modulation in the human lung adenocarcinoma
Source: PLoS Comput Biol. 2021 Jun 22;17(6):e1009091. doi: 10.1371/journal.pcbi.1009091 (PMC8219159; doi:10.1371/journal.pcbi.1009091)
Supplement: S1 Table — Rate constants for transitions between the states of all hidden Markov models. For calcium dependent transitions the calculated steady state calcium concentration Ca_i = 4.68 μM is used. (DOCX) [file pcbi.1009091.s003.docx]

**S1 Table. Parameters of hidden Markov models used in the A549 whole-cell current model.** Rate constants for transitions between the states of all hidden Markov models. For calcium dependent transitions the calculated steady state calcium concentration *Ca_i* = 4.68 µM is used.

| **Kv1.3 kinetic model** [1] | | **TASK-1 kinetic model** [2] | |
| --- | --- | --- | --- |
| $\alpha=0.448\cdot exp(\frac{V}{27.530})$ ms^-1^ | $\beta=0.43\cdot exp(\frac{-V}{17.528})$ ms^-1^ | $\alpha=0.0133\cdot exp(-0.106\frac{VF}{RT})$ ms^-1^ | $\beta=0.0176\cdot exp(0.105\frac{VF}{RT})$ ms^-1^ |
| $a=280.035$ ms^-1^ | $b=1.648$ms^-1^ | $a=0.108\cdot exp(-0.095\frac{VF}{RT})$ ms^-1^ | $b=0.0097\cdot exp(0.307\frac{VF}{RT})$ ms^-1^ |
| $\eta=0.003\cdot exp(\frac{V}{174.961})$ ms^-1^ | $\lambda=0.00008\cdot exp(\frac{-V}{1016.33})$ ms^-1^ | **KCa1.1 kinetic model** [3] | |
| **Kv3.1 kinetic model** [4] | | $c0=1.8225\cdot exp(-\frac{V}{d})$ ms^-1^ | $a0=0.001\cdot exp(\frac{V}{b})$ ms^-1^ |
| $\alpha=0.9260\cdot exp(\frac{V}{35.8932})$ ms^-1^ | $\beta=0.1941\cdot exp(\frac{-V}{279.7353})$ ms^-1^ | $c1=1.215\cdot exp(-\frac{V}{d})$ ms^-1^ | $a1=0.006\cdot exp(\frac{V}{b})$ ms^-1^ |
| $a=40.5586$ ms^-1^ | $b=389.27$ms^-1^ | $c2=0.855\cdot exp(-\frac{V}{d})$ ms^-1^ | $a2=0.038\cdot exp(\frac{V}{b})$ ms^-1^ |
| **Kv3.4 kinetic model** [5] | | $c3=0.49\cdot exp(-\frac{V}{d})$ ms^-1^ | $a3=0.196\cdot exp(\frac{V}{b})$ ms^-1^ |
| $\alpha=3.352\cdot exp(\frac{0.06VF}{RT})$ ms^-1^ | $\beta=3.230\cdot exp(\frac{-0.80VF}{RT})$ ms^-1^ | $c4=0.11\cdot exp(-\frac{V}{d})$ ms^-1^ | $a4=0.396\cdot exp(\frac{V}{b})$ ms^-1^ |
| $a=0.434\cdot exp(\frac{0.52VF}{RT})$ ms^-1^ | $b=0.070\cdot exp(\frac{-0.37VF}{RT})$ ms^-1^ | *d* = 200 mV | *b* = 36 mV |
| $\eta=0.055$ ms^-1^ | $\lambda=0.00080$ ms^-1^ | *kc*= 13.5 $\cdot$*Ca_rate* ms^-1^ | *ko* = 1.5$\cdot$*Ca_rate* ms^-1^ |
| **Kv7.1 kinetic model** [6] | | *Ca* = *Ca_i* $\cdot$*Ca_rate* ms^-1^ | *Ca_rate* = 1 µM^-1^ms^-1^ |
| $\alpha=4.6\cdot exp(0.47\frac{VF}{RT})$ ms^-1^ | $\beta=33\cdot exp(-0.35\frac{VF}{RT})$ ms^-1^ | **KCa3.1 kinetic model** [7] | |
| $a=24\cdot exp(0.006\frac{VF}{RT})$ ms^-1^ | $b=19\cdot exp(-0.007\frac{VF}{RT})$ ms^-1^ | $\alpha1=27\cdot Ca\_i$ ms^-1^ | $\beta1=34$ms^-1^ |
| $c=4.6\cdot exp(0.8\frac{VF}{RT})$ ms^-1^ | $d=1.4\cdot exp(-0.7\frac{VF}{RT})$ ms^-1^ | $\alpha2=5425\cdot Ca\_i$ ms^-1^ | $\beta2=190$ ms^-1^ |
| $\eta=142$ ms^-1^ | $\lambda=52$ ms^-1^ | $a=34\cdot Ca\_i$ ms^-1^ | *Ca_i* = 4.68 µM |
| **CLC-2 kinetic model** [8] | | *b* = 20 ms^-1^ |  |
| $\alpha1=0.0041\cdot exp(\frac{-0.57VF}{RT})$ ms^-1^ | $\beta1=0.1003\cdot exp(\frac{0.18VF}{RT})$ ms^-1^ | **CRACM1 kinetic model** [9] | |
| $\alpha2=0.0064\cdot exp(\frac{-0.2VF}{RT})$ ms^-1^ | $\beta2=0.0106\cdot\exp\left( \frac{0.32VF}{RT} \right)m$s^-1^ | $a=41\cdot exp(\frac{V}{110})$ ms^-1^ | $b=19\cdot exp(\frac{V}{48})$ ms^-1^ |
| $\lambda=0.0017\cdot\exp\left( \frac{-0.3VF}{RT} \right)+0.0088\cdot\exp\left( \frac{0.14VF}{RT} \right) m$s^-1^ | $\mu=0.0049\cdot exp(\frac{0.18VF}{RT})$ ms^-1^ |  |  |

**Supporting References**

1. Hou P, Zhang R, Liu Y, Feng J, Wang W, Wu Y, et al. Physiological role of Kv1.3 channel in T lymphocyte cell investigated quantitatively by kinetic modeling. PLoS ONE. 2014;9: e89975. doi:10.1371/journal.pone.0089975

2. Limberg SH, Netter MF, Rolfes C, Rinné S, Schlichthörl G, Zuzarte M, et al. TASK-1 channels may modulate action potential duration of human atrial cardiomyocytes. Cell Physiol Biochem. 2011;28: 613–624. doi:10.1159/000335757

3. Wang W, Xiao F, Zeng X, Yao J, Yuchi M, Ding J. Optimal estimation of ion-channel kinetics from macroscopic currents. PLoS ONE. 2012;7: e35208. doi:10.1371/journal.pone.0035208

4. Wang W, Luo J, Hou P, Yang Y, Xiao F, Yuchi M, et al. Native gating behavior of ion channels in neurons with null-deviation modeling. PLoS ONE. 2013;8: e77105. doi:10.1371/journal.pone.0077105

5. Fineberg JD, Ritter DM, Covarrubias M. Modeling-independent elucidation of inactivation pathways in recombinant and native A-type Kv channels. J Gen Physiol. 2012;140: 513–527. doi:10.1085/jgp.201210869

6. Pusch M, Magrassi R, Wollnik B, Conti F. Activation and inactivation of homomeric KvLQT1 potassium channels. Biophys J. 1998;75: 785–792. doi:10.1016/S0006-3495(98)77568-X

7. Bailey MA, Grabe M, Devor DC. Characterization of the PCMBS-dependent modification of KCa3.1 channel gating. J Gen Physiol. 2010;136: 367–387. doi:10.1085/jgp.201010430

8. de Santiago JA, Nehrke K, Arreola J. Quantitative analysis of the voltage-dependent gating of mouse parotid ClC-2 chloride channel. J Gen Physiol. 2005;126: 591–603. doi:10.1085/jgp.200509310

9. Fomina AF, Fanger CM, Kozak JA, Cahalan MD. Single Channel Properties and Regulated Expression of Ca2+ Release-Activated Ca2+ (Crac) Channels in Human T Cells. J Cell Biol. 2000;150: 1435–1444. doi:10.1083/jcb.150.6.1435
