# Supplementary material for: A549 in-silico 1.0: A first computational model to simulate cell cycle dependent ion current modulation in the human lung adenocarcinoma
Source: PLoS Comput Biol. 2021 Jun 22;17(6):e1009091. doi: 10.1371/journal.pcbi.1009091 (PMC8219159; doi:10.1371/journal.pcbi.1009091)
Supplement: S2 Table — Resting potentials of current-clamp measurements, reversal potentials from voltage-ramp measurements (ramp potential) and reversal potentials derived from current-voltage curves (reversal potential) of the individual cells with negative resting potential in G0 and positive resting potential in G1 phase. (DOCX) [file pcbi.1009091.s004.docx]

**S2 Table. Patch-clamp results and statistical analysis.** Resting potentials of current-clamp measurements, reversal potentials from voltage-ramp measurements (ramp potential) and reversal potentials derived from current-voltage curves (reversal potential) of the individual cells with negative resting potential in G0 and positive resting potential in G1 phase.

| **G0 phase** | **Measured** | | | **G1 phase** | **Measured** | | |
| --- | --- | --- | --- | --- | --- | --- | --- |
| Cell ID | Resting potential | Ramp potential | Reversal potential | Cell ID | Resting potential | Ramp potential | Reversal potential |
| G0_1 | -28 | -3.3 | -4 | G1_1 | +7.5 | -0.93 | +0.9 |
| G0_2 | -20 | -3.4 | -1.4 | G1_2 | +6.5 | +8.4 | +9.9 |
| G0_3 | -15 | -20.8 | -15.4 | G1_3 | +14 | +3.6 | 0 |
| G0_4 | -17 | -2.3 | -8 | G1_4 | +6 | -2.8 | -4.3 |
| G0_5 | -20 | x | -4.8 | G1_5 | +16 | +6.3 | +3.3 |
| G0_6 | -2 | -2 | -4 |  | | | |
| G0_7 | -15 | -15.7 | -11.5 |  |  |  |  |
| G0_8 | -9 | -6.4 | -7.5 |  |  |  |  |
| G0_9 | -8 | x | -10.1 |  |  |  |  |
| G0_10 | -35 | -13.7 | -21.1 |  |  |  |  |
| G0_11 | -40 | -35 | -38 |  |  |  |  |
| Median | -17.0 | -6.41 | -8.0 | Median | +7.5 | +3.6 | +0.9 |
| Mean | -19.0 | -11.4 | -11.4 | Mean | +10.0 | +2.9 | +1.9 |
| Standard deviation | ±10.98 | ±10.51 | ±10.01 | Standard deviation | ±4.16 | ±4.25 | ±4.7 |
| Normal distribution | | | | | | | |
| Shapiro Wilk test | normal | not normal | not normal | Shapiro Wilk test | normal | not normal | not normal |
| Kolmogorov Smirnow test | normal | normal | normal | Kolmogorov Smirnow test | normal | normal | normal |
| Statistical significance | | | | | | | |
| Student-t test | significant  p=0.000051  t=5.3529 | x | x | Student-t test | significant  p=0.000051  t=5.3529 | x | x |
| Mann Whitney U test | x | significant  p=0.00758 | significant  p=0.00652 | Mann Whitney U test | x | significant  p=0.00758 | significant  p=0.00652 |
