# Supplementary material for: A549 in-silico 1.0: A first computational model to simulate cell cycle dependent ion current modulation in the human lung adenocarcinoma
Source: PLoS Comput Biol. 2021 Jun 22;17(6):e1009091. doi: 10.1371/journal.pcbi.1009091 (PMC8219159; doi:10.1371/journal.pcbi.1009091)
Supplement: S3 Table — (DOCX) [file pcbi.1009091.s005.docx]

**S3 Table. Comparison of measured and calculated membrane potentials in the different cell cycle phases**

|  | **Measured resting potential** | **Reversal potential derived from**  **current-voltage curves** | **Reversal potential**  **derived from the measured ramp protocol** | **Calculated membrane potential** | **Simulated membrane potential** |
| --- | --- | --- | --- | --- | --- |
|  | ***V*_rest_ [mV]** | ***V*_rev_ [mV]** | ***V*_rev_ [mV]** | ***V*_m_ [mV]** | ***V*_m_ [mV]** |
| **G0** | **-19.0** | **-11.4** | **-11.4** | **-10.483** | **-10.398** |
| **G1** | **+10.0** | **+1.9** | **+2.9** | **-1.337** | **-1.258** |
| **S** | **-** | **-** | **-** | **-13.275** | **-13.20** |
| **G2/M** | **-** | **-** | **-** | **-5.497** | **-5.263** |
