# Supplementary figures and images for: A549 in-silico 1.0: A first computational model to simulate cell cycle dependent ion current modulation in the human lung adenocarcinoma
Source: PLoS Comput Biol. 2021 Jun 22;17(6):e1009091. doi: 10.1371/journal.pcbi.1009091 (PMC8219159; doi:10.1371/journal.pcbi.1009091)

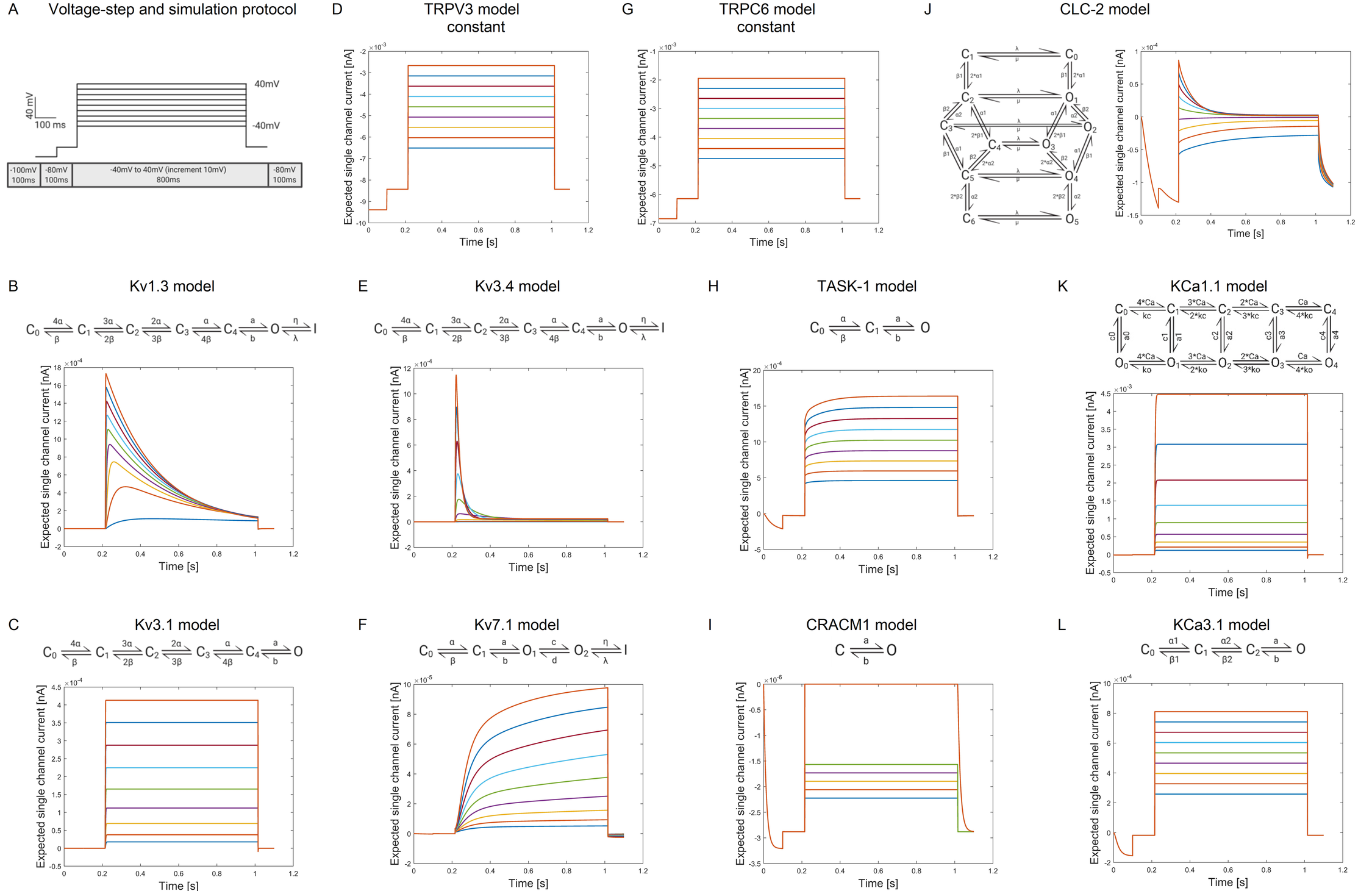

Supplement: S1 Fig — (A) Voltage-step protocol of patch-clamp measurements and below the corresponding protocol for model simulation. Kinetic scheme and expected single channel currents for voltage levels between -40 mV to +40 mV of the ion channels (B) Kv1.3, (C) Kv3.1, (D) TRPV3, (E) Kv3.4, (F) Kv7.1, (G) TRPC6, (H) TASK-1, (I) CRACM1, (J) CLC-2, (K) KCa1.1 and (L) KCa3.1. (TIF) [file pcbi.1009091.s006.tif]

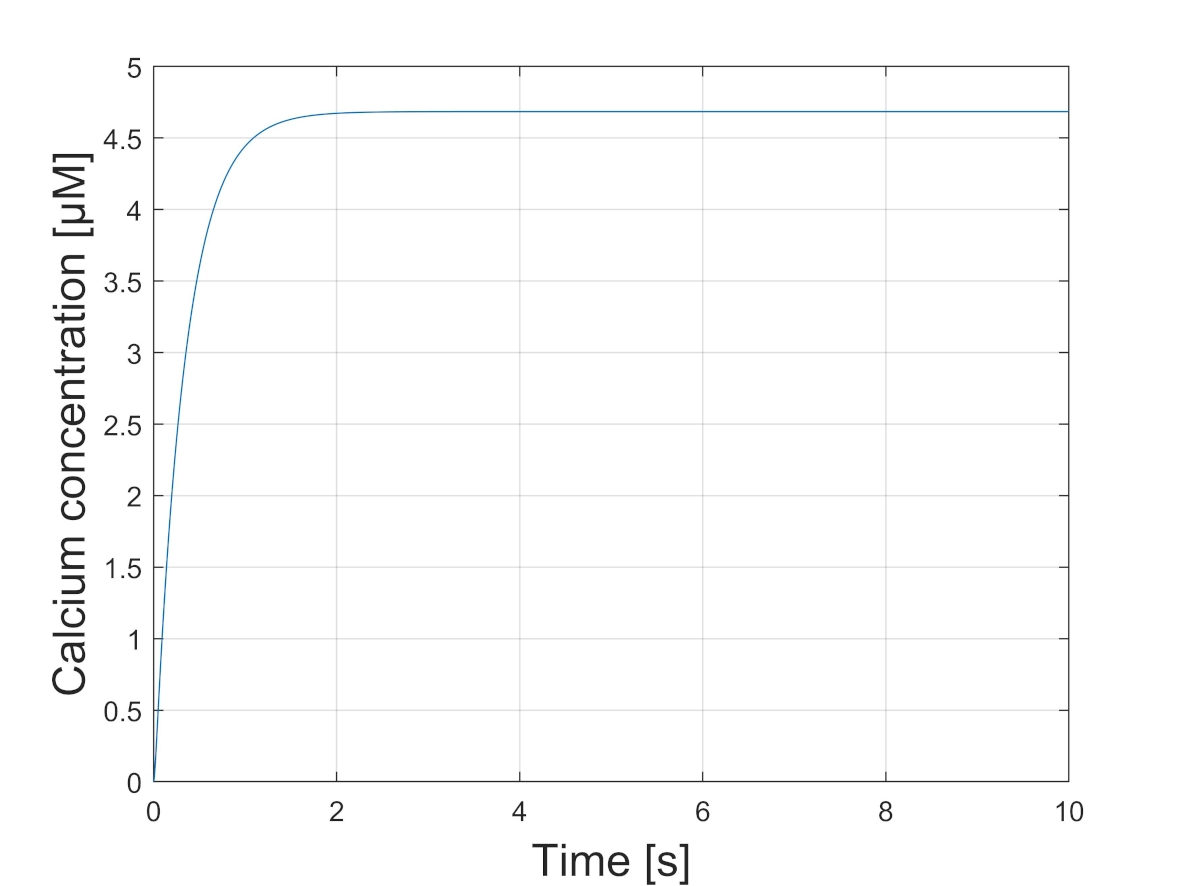

Supplement: S2 Fig — Simulated changes of the local calcium concentration provoked by CRAC channels at holding potential of -100 mV over 10 s. Starting point c[Ca2+]i = 0.0647 μM, steady state c[Ca2+]i = 4.6847 μM, etrans = 21.8976∙10−3 μMpA-1ms-1L-1, ediff = 3∙10−3 ms-1. (TIF) [file pcbi.1009091.s007.tif]

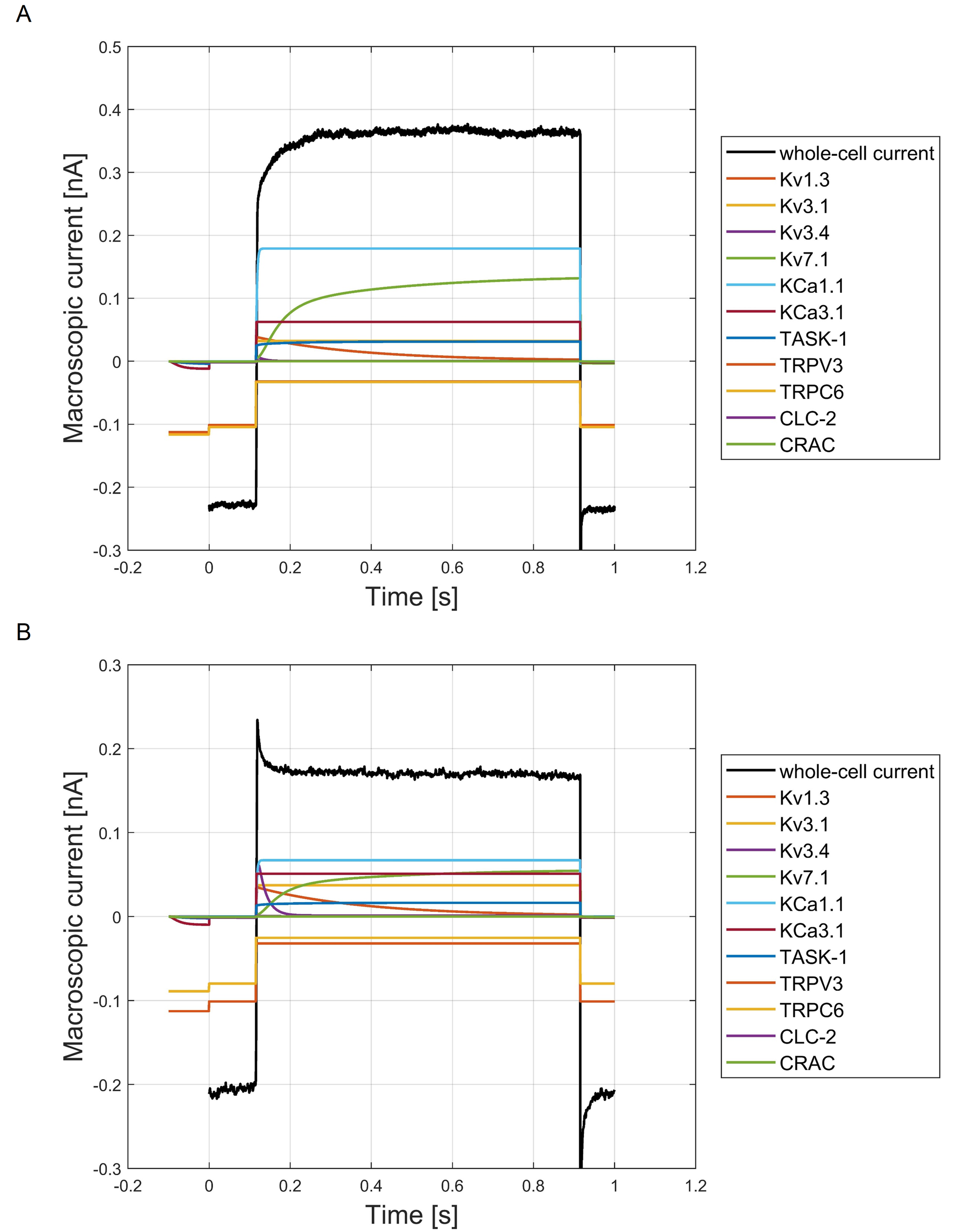

Supplement: S3 Fig — Macroscopic currents of single ion channels, estimated by model optimization in respect to whole-cell current for A: G0 phase and B: G1 phase at +40 mV. (TIF) [file pcbi.1009091.s008.tif]

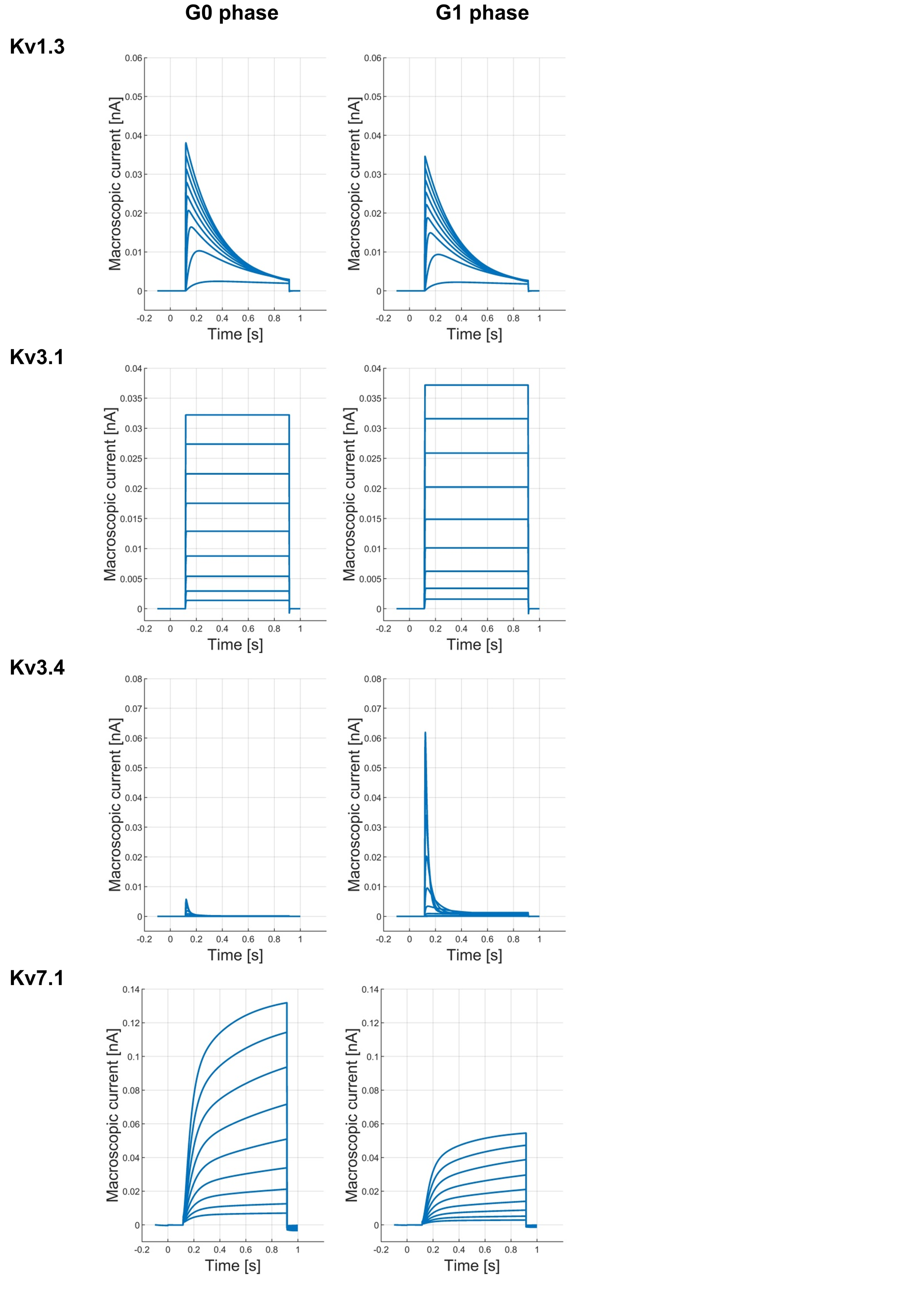

Supplement: S4 Fig — Comparison of macroscopic currents of voltage-gated potassium channels Kv1.3, Kv3.1, Kv3.4 and Kv7.1 in G0 and G1 phase at voltage levels from -40 mV to +40 mV. (TIF) [file pcbi.1009091.s009.tif]

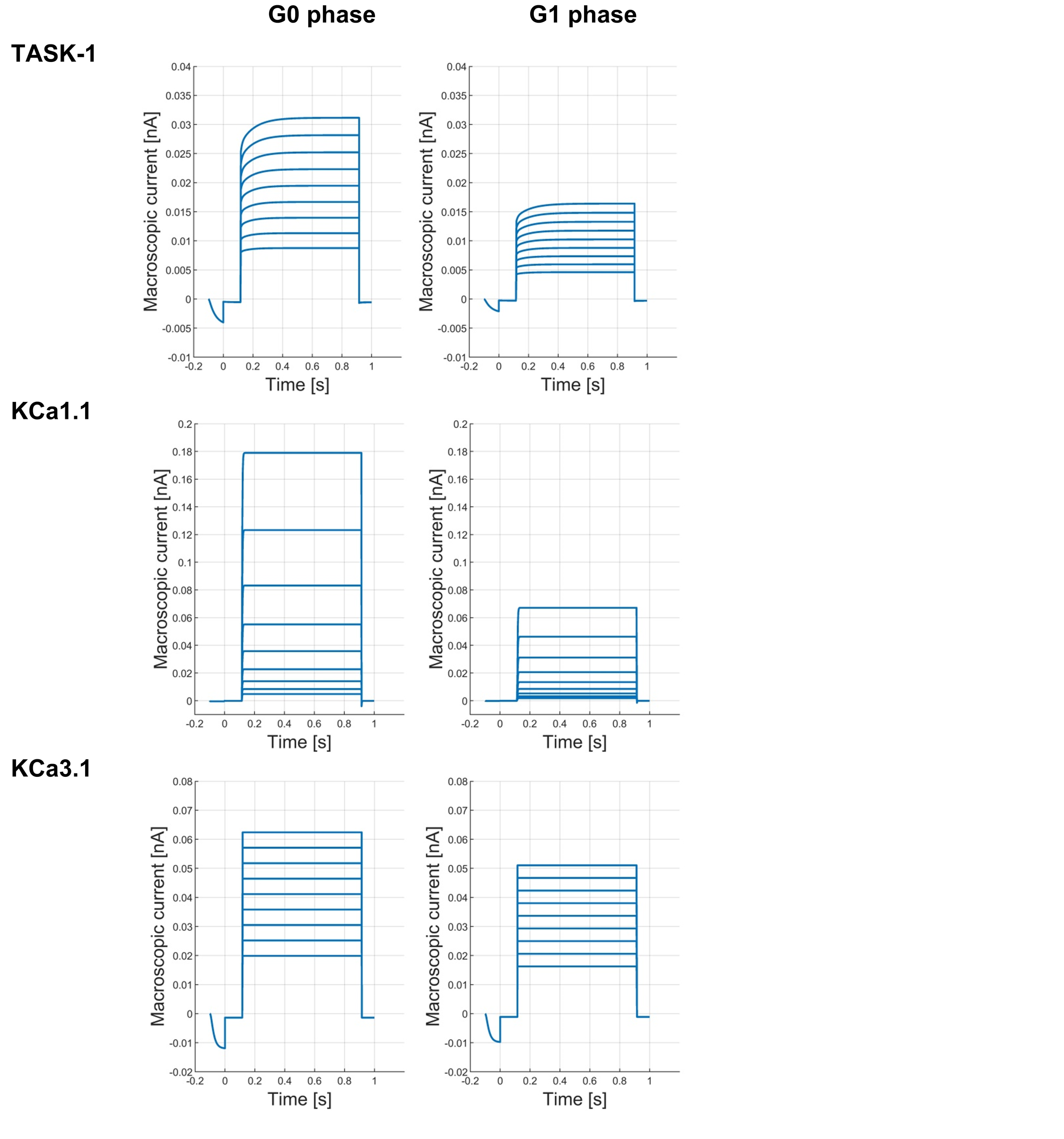

Supplement: S5 Fig — Comparison of macroscopic currents of potassium channels TASK-1, KCa1.1 and KCa3.1 in G0 and G1 phase at voltage levels from -40 mV to +40 mV. (TIF) [file pcbi.1009091.s010.tif]

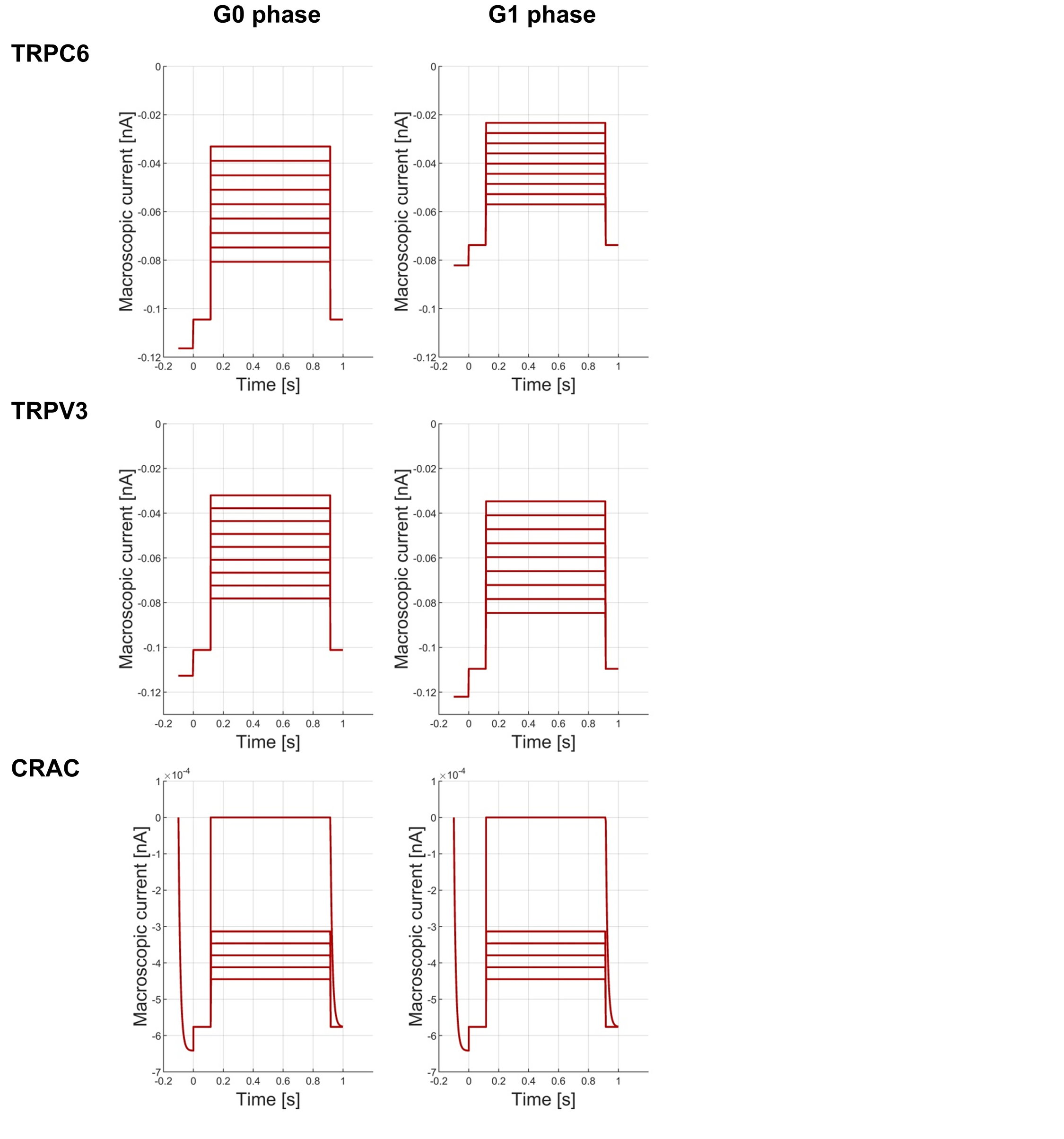

Supplement: S6 Fig — Comparison of macroscopic currents of calcium channels TRPC6, TRPV3 and CRAC in G0 and G1 phase at voltage levels from -40 mV to +40 mV. (TIF) [file pcbi.1009091.s011.tif]

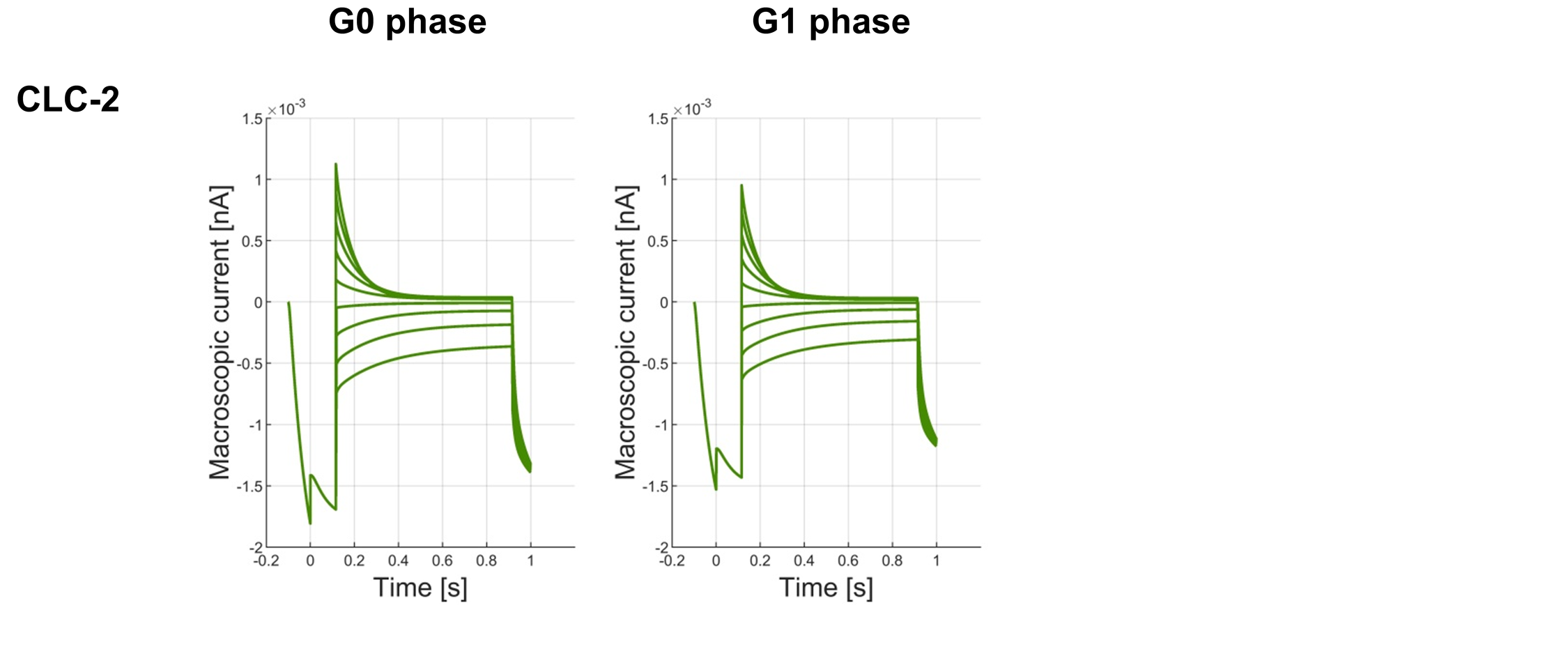

Supplement: S7 Fig — Comparison of the macroscopic CLC-2 chloride current in G0 and G1 phase at voltage levels from -40 mV to +40 mV. (TIF) [file pcbi.1009091.s012.tif]

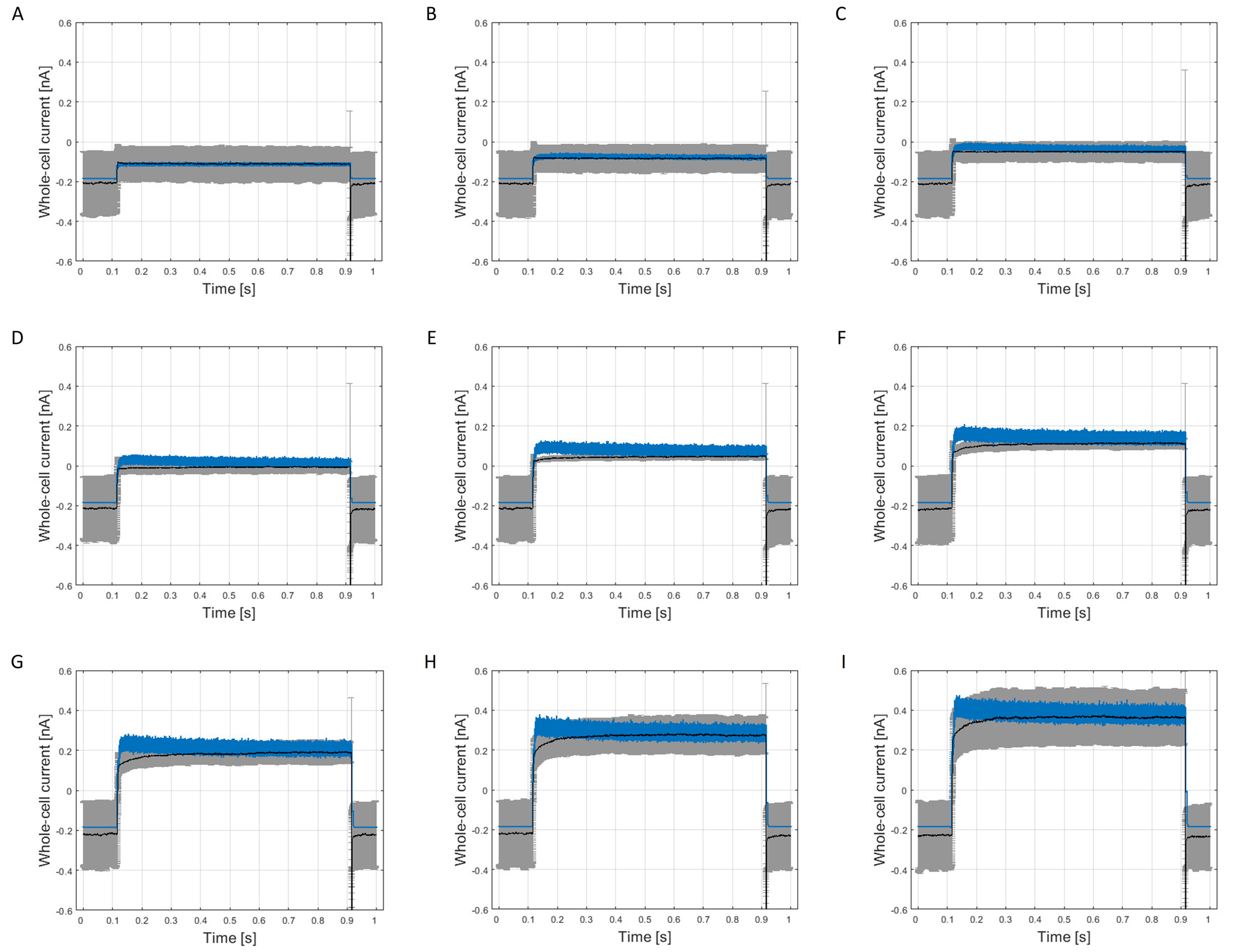

Supplement: S8 Fig — Simulated whole-cell current of 100 sample cells at (A) -40 mV, (B) -30 mV, (C) -20 mV, (D) -10 mV, (E) 0 mV, (F) +10 mV, (G) +20 mV, (H) +30 mV, (I) +40 mV for G0 phase. Black lines show the averaged measured whole-cell cell currents, background indicates the corresponding standard deviations at all voltage levels. (TIF) [file pcbi.1009091.s013.tif]

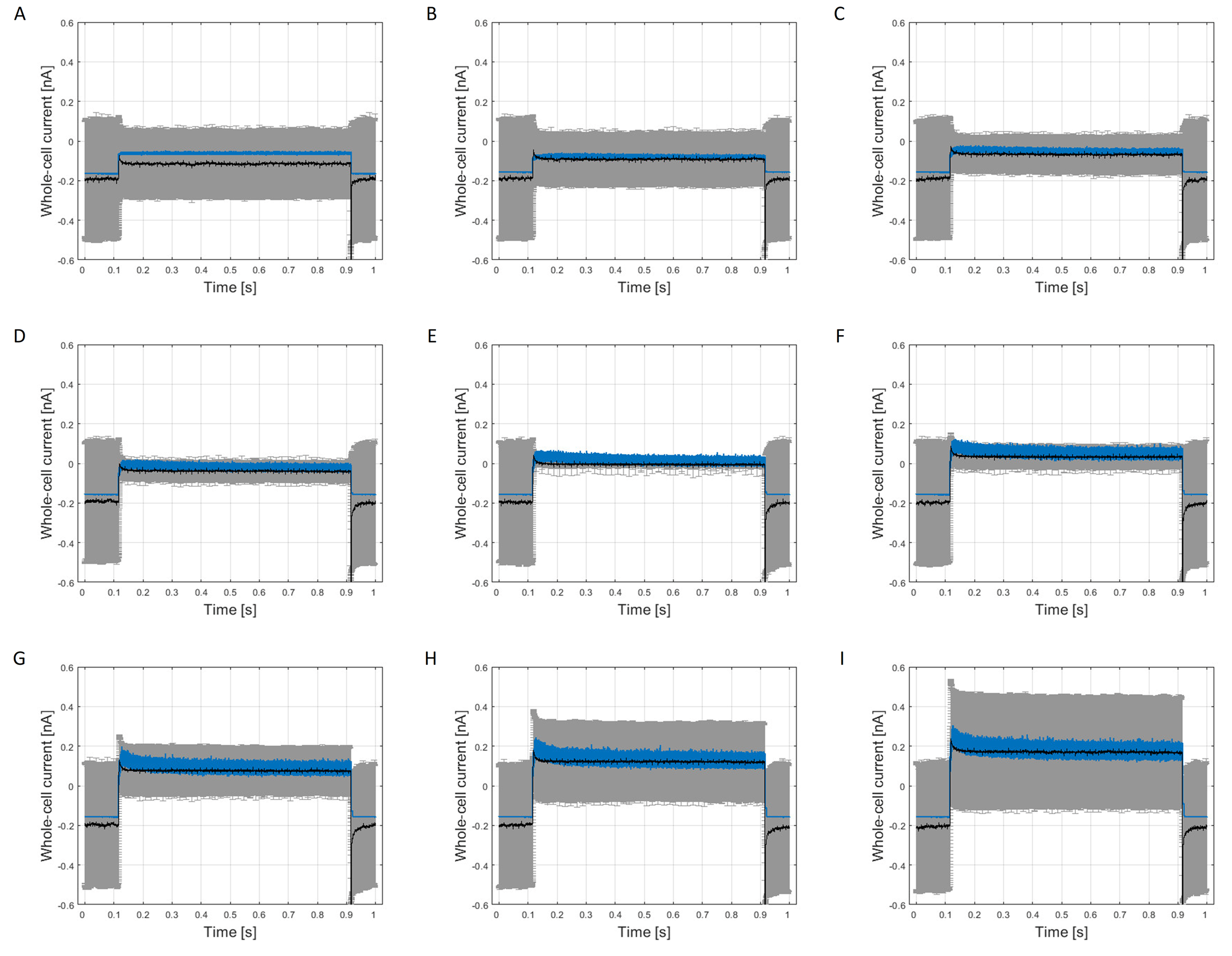

Supplement: S9 Fig — Simulated whole-cell current of 100 sample cells at (A) -40 mV, (B) -30 mV, (C) -20 mV, (D) -10 mV, (E) 0 mV, (F) +10 mV, (G) +20 mV, (H) +30 mV, (I) +40 mV for G1 phase. Black curves represent the averaged whole-cell currents, grey background indicates the corresponding standard deviations at all voltage levels. (TIF) [file pcbi.1009091.s014.tif]

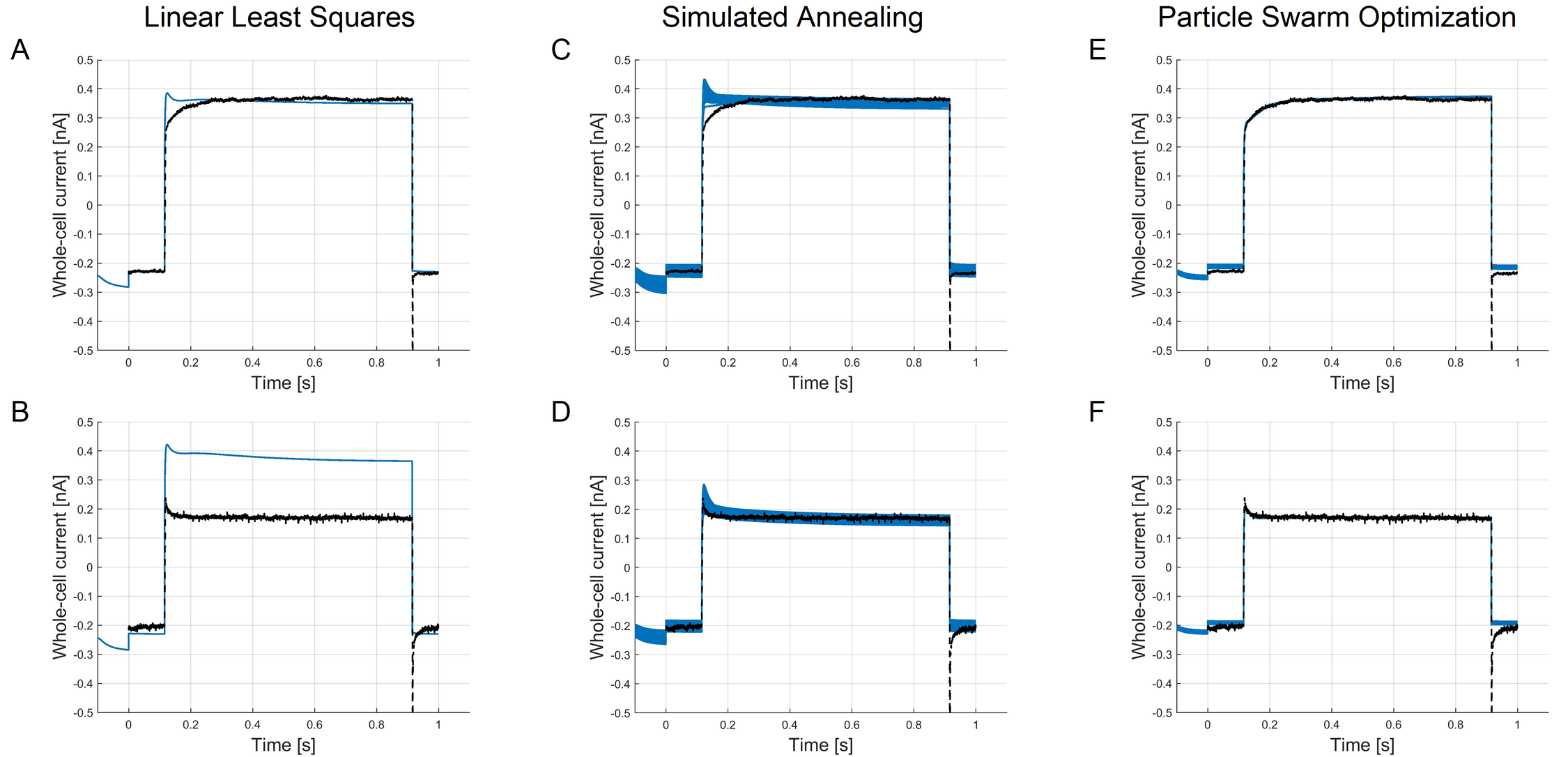

Supplement: S10 Fig — Fitting results (n = 100 simulation runs) of (A, B) lsqlin, (C, D) SA and (E, F) PSO for averaged whole-cell currents of cells in G0 and G1 phase. (TIF) [file pcbi.1009091.s015.tif]

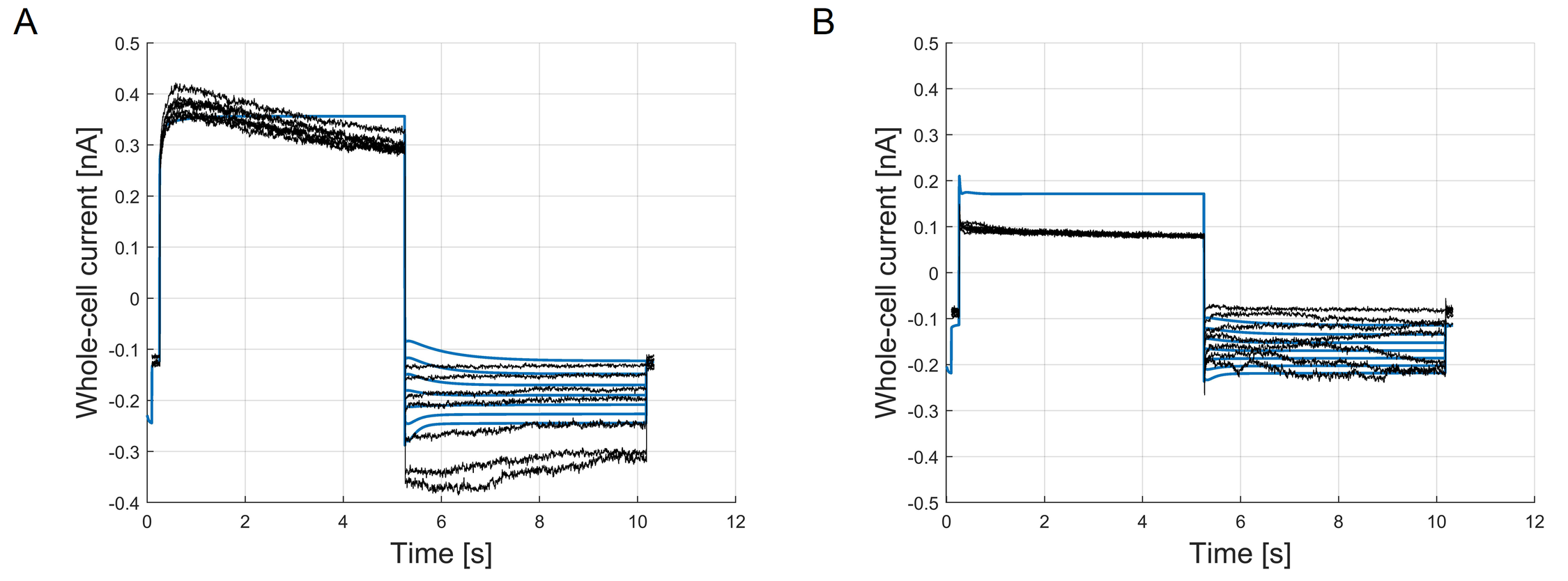

Supplement: S11 Fig — Simulation of the deactivation protocols for (A) G0 (n = 5, RMSEG0 = 0.0754) and (B) G1 (n = 3, RMSEG1 = 0.0673) phase. Deviations can be explained by the small sample size and apparent leakage currents for strongly negative deactivation pulses below -80 mV. (TIF) [file pcbi.1009091.s016.tif]

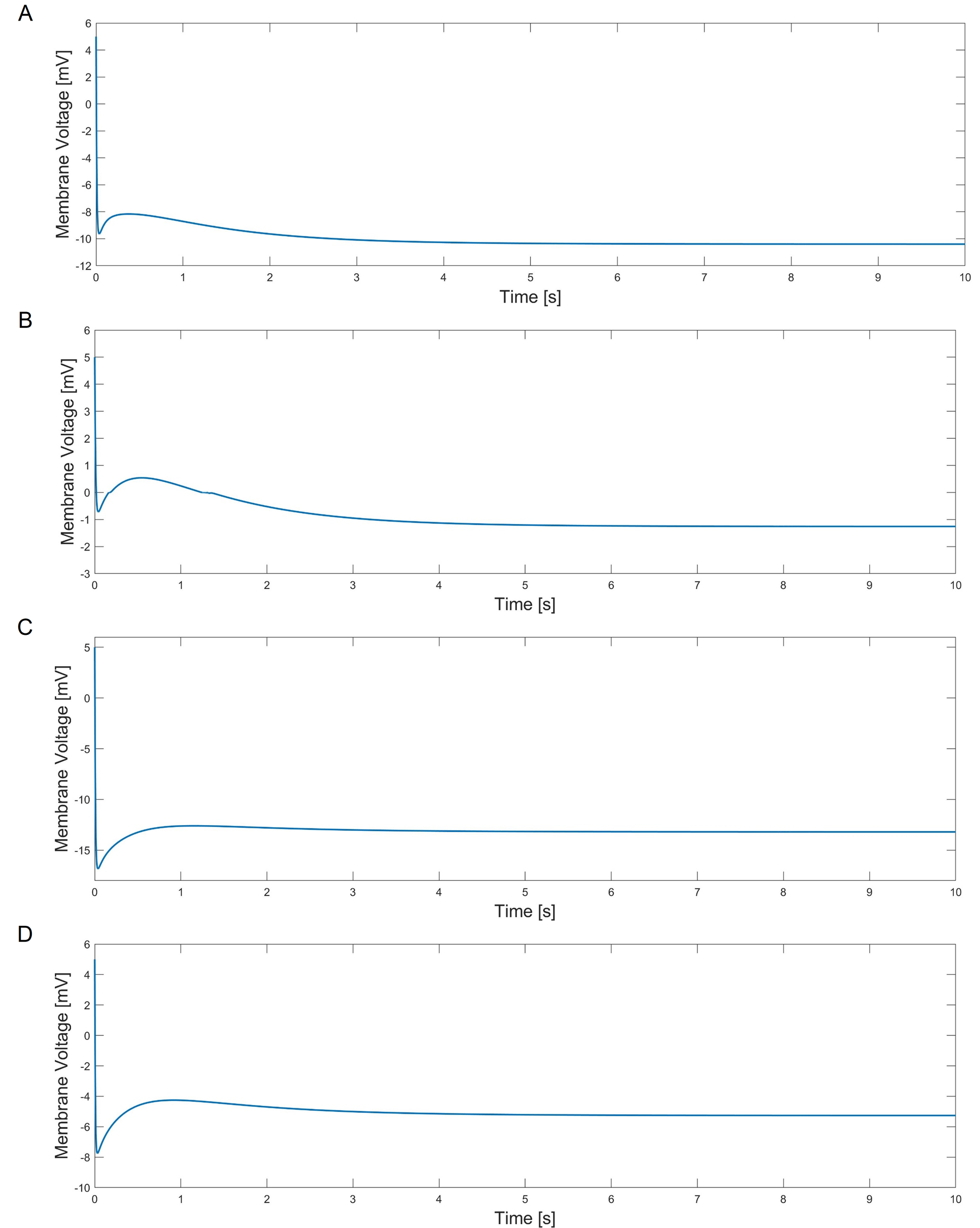

Supplement: S12 Fig — Simulated membrane potential (starting point at 5 mV over 10 s (dt = 5.10−7)) for (A) G0 phase Vm = -10.398 mV, (B) G1 phase Vm = -1.258 mV, (C) S phase Vm = -13.2 and mV (D) G2/M phase Vm = -5.263 mV. (TIF) [file pcbi.1009091.s017.tif]
